# Solution and Active Site Speciation Drive Selectivity for Electrocatalytic Reactive Carbon Capture in Diethanolamine over Ni–N–C Catalysts

**DOI:** 10.1021/jacs.5c11791

**Published:** 2026-01-22

**Authors:** R. Dominic Ross, Yulan Han, Hui-Yun Jeong, Jenna M. Ynzunza, Robert H. Lavroff, Avishek Banerjee, Aditya Prajapati, Carlos G. Morales-Guio, Jesús M. Velázquez, Anastassia N. Alexandrova, Christopher Hahn

**Affiliations:** † Materials Science Division, 4578 Lawrence Livermore National Laboratory, Livermore, California 94550, United States; ‡ Laboratory for Energy Applications for the Future (LEAF), Lawrence Livermore National Laboratory, Livermore, California 94550, United States; § Department of Chemistry and Biochemistry, 8783 University of California, Los Angeles, Los Angeles, California 90095, United States; ∥ Department of Chemistry, 8789 University of California, Davis, Davis, California 95616, United States; ⊥ Department of Chemical and Biomolecular Engineering, University of California, Los Angeles, Los Angeles, California 90095, United States

## Abstract

Direct conversion of captured forms of carbon, or reactive carbon capture (RCC), presents an opportunity to reduce the energy intensity and cost of direct CO_2_ utilization from dilute sources. While amine-based sorbents effectively capture CO_2_, their use for RCC presents numerous challenges with typical pure metal catalysts used for electrochemical CO_2_ reduction (CO_2_R). Here, using both theory and experiments, we find that Ni–N–C single atom catalysts are effective for RCC conversion to CO using a diethanolamine sorbent, in contrast to pure metal catalysts. Computational analysis reveals that RCC can proceed directly through direct reduction of the sorbent-CO_2_ adduct or indirectly by C–N bond breaking facilitating CO_2_ adsorption and subsequent reduction. We find that the latter mechanism is most prevalent at low overpotentials where we experimentally observe RCC selectivity. We also find experimentally that the rate of CO production for RCC with Ni–N–C catalysts can exceed pure bicarbonate solutions at intermediate sorbent concentration (0.1–0.5 M DEA) under dilute (10–25%) streams of CO_2_ at low overpotentials. The coordination environment of Ni sites and the solution speciation influence their RCC activity, with changes in protonation to coordinating N/C atoms resulting in changing the RCC mechanism and consequent activity. *In situ* X-ray absorption spectroscopy and computational analysis reveal restructuring under RCC conditions due to hydrogen coadsorption with DEA that limits the stability of Ni–N–C catalysts. This work highlights the importance of carefully controlling the catalyst and solution environment to achieve active and stable RCC electrocatalysis.

## Graphical abstract

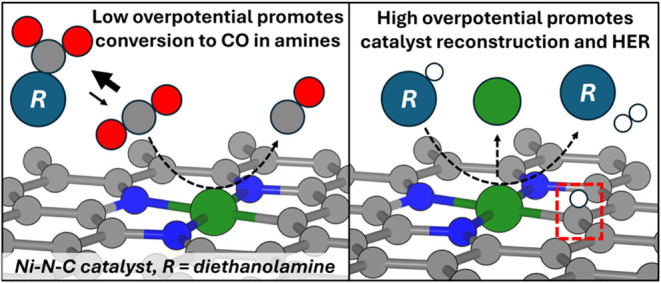

## Introduction

Processes powered by renewable electricity to capture and convert carbon dioxide (CO_2_) from the atmosphere are urgently needed to mitigate the impacts of climate change. Benchmark sorbents for carbon capture are typically amines such as monoethanolamine (MEA) or diethanolamine (DEA), on account of their high affinity for CO_2_.^1^ The capture of CO_2_ by amines generates CO_2_-amine adducts (i.e., carbamates). For each negatively charged carbamate, an equivalent amount of the protonated amine is generated (Scheme 1). The highly thermodynamically stable carbamate also results in an energy-intensive regeneration step needed to achieve CO_2_-lean amine and pure CO_2_ from the capture solution.^2,3^ Reactive carbon capture (RCC) integrates capture and conversion by directly reacting CO_2_ in the capture media, subverting the need for successive capture, sorbent regeneration, and CO_2_ conversion.^4–6^ This process presents the opportunity to reduce the cost and energy intensity of achieving carbon-neutral industrial processes^6–8^ by continuously valorizing CO_2_ from industrial point sources (i.e., flue gas) such as cement production^9^ and power generation.^10^ The concentrations of CO_2_ present in these streams are typically too dilute for efficient electrocatalytic conversion directly,^11^ and often contain contaminants that poison typical catalysts for direct CO_2_ electroreduction.^12^

**Scheme 1:**
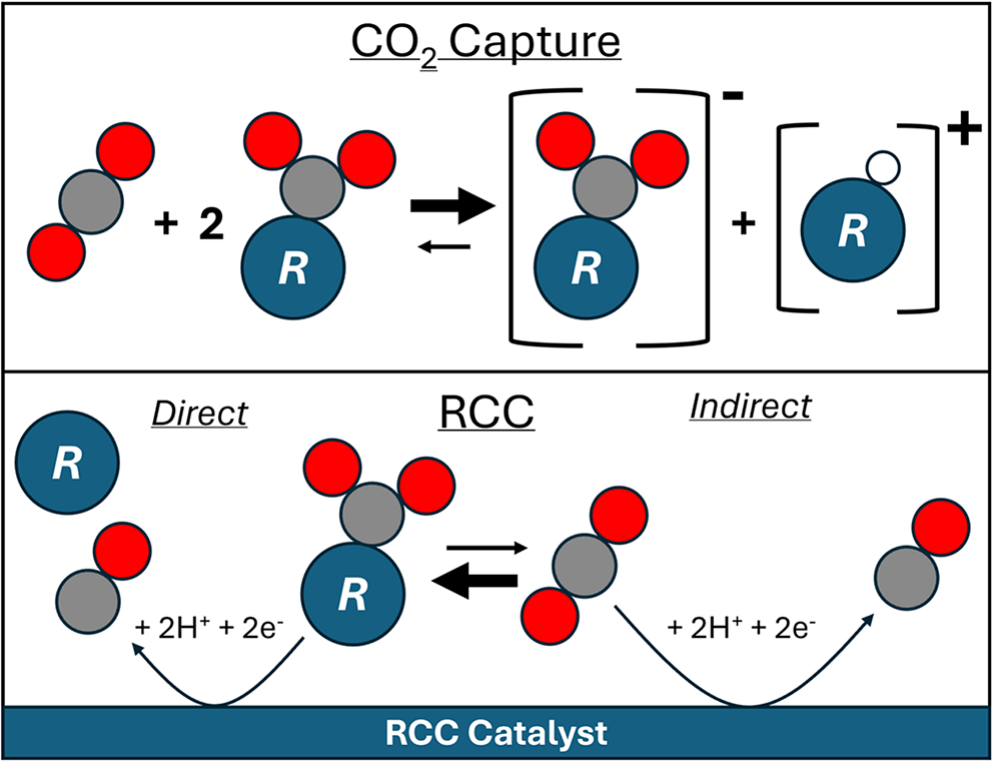
CO_2_ Capture Process with Amines (Top, R = Primary or Secondary Amine) and Reactive Carbon Capture (RCC, Bottom)^a^

a RCC can proceed through the direct mechanism (left) where the CO_2_-amine adduct (carbamate) binds to the catalyst surface and is reduced, or an indirect mechanism (right) where CO_2_ adsorbates are produced which can subsequently be reduced. Gray = carbon, red = oxygen, white = hydrogen.

Pure metals that are typically exemplar electrochemical CO_2_ reduction (CO_2_R) catalysts exhibit negligible selectivity for RCC in amines, especially in the absence of an additional incoming CO_2_ source.^13–19^ The primary causes are the promotion of HER by protonated amines,^13,15,16^ the capture of free CO_2_ near the interface by lean amines,^16^ the difficulty in directly reducing thermodynamically stable carbamates,^14^ and the corrosion of metal surfaces.^15,20^ To achieve effective RCC with amines, some strategies to suppress HER by changing the catalyst microenvironment have been explored, such as the use of cationic (ammonium-based) surfactants,^17^ larger alkali metal cations,^18,21,22^ nonaqueous solvents,^23,24^ molecular catalysts (in nonaqueous solvents),^25^ and sterically hindered amines.^24,26,27^ However, no single approach has achieved both high rates of reaction and high selectivity for carbon based products using amines as a sorbent.

Metal–nitrogen doped carbon (M–N-C) single atom catalysts (SACs) have been utilized widely in CO_2_ reduction, achieving nearly >90% selectivity for CO at high current density.^28–31^ This is largely due to effective suppression of HER by weakened H* adsorption on coordinated metal centers. Similarly, Ni–N–C SACs have been demonstrated to have some selectivity toward carbon-based products under RCC conditions.^27,32–34^ More broadly, SACs also have different active site structures (i.e., NiN_*x*_C_4–*x*_) depending on the exact synthesis conditions.^35,36^ The significant variability in active site ensembles complicates the analysis of these catalysts for RCC. While these catalysts are stable for CO_2_R, their stability under RCC conditions remains largely unknown. There are some examples of achieving relatively high conversion rates over Ni–N–C catalysts with amine based sorbents, although higher rates of conversion to CO with moderate selectivity typically require the use of proton exchange membrane (PEM) or a bipolar membrane (BPM) to liberate and directly react free CO_2_ from the capture solution.^32,34^ More recently, the use of diamines that can form zwitterionic carbamates at high CO_2_ concentrations has been demonstrated as a route to direct reduction of amine-captured CO_2_.^27^ The direct reduction route of these amines was demonstrated by computation and the ability to obtain high rates of CO production in an anion exchange membrane (AEM) based electrolyzer. These works suggest promise for improving carbon utilization by using selective RCC systems, particularly where carbamates can be directly reduced, which warrants further exploration of the underlying active species under different operating conditions in order to ensure long-term operation. Performance of amine-based RCC systems is highly variable depending on the partial pressure of CO_2_ in the capture solution.^19^ RCC is often demonstrated under CO_2_ saturated conditions, which evaluates resiliency to operating in amines, but overlooks evaluating the prevalence of the RCC pathway, in which carbamates can be adsorbed to the catalyst surface and subsequently converted to carbon-based products (Scheme 1), as opposed to reducing free CO_2_ in the presence of amines. Understanding the surface intermediates formed from amine-based RCC on Ni–N–C catalysts in terms of both energetics (i.e., activity and selectivity) as well as their impact on structural evolution (i.e., stability) will be crucial to further development of these systems. Additionally, dynamic speciation of the electrolyte due to applied current/voltage and different CO_2_ partial pressures will also impact these mechanistic pathways, demanding an in depth understanding of how the catalyst structure and performance change under different operating conditions.

In this study, we demonstrate that Ni–N–C catalysts can partially suppress HER in diethanolamine (DEA), producing orders of magnitude higher CO selectivity than that observed over pure metal catalysts and enhancing the rate for direct conversion of dilute streams of CO_2_. Using theory, we evaluate different RCC pathways, and find that they are heavily influenced by the initial defect structure of the Ni–N–C catalyst and the applied potential. Importantly, applied potential influences the protonation environment on coordinating carbons around Ni sites, influencing the viability of RCC and the stability of Ni sites. By varying the CO_2_ and DEA concentrations during experimental testing and using vapor–liquid equilibrium (VLE) modeling, we find further evidence that while free CO_2_ (i.e., CO_2_ not chemisorbed to an amine when it is reduced) can contribute to CO production over Ni–N–C catalysts, there is a rate enhancement driven by RCC. This observation is congruent with theory suggesting RCC should be preferred to conventional CO_2_R and HER across a broad potential range. Through theoretical simulations and *in situ* experimental X-ray absorption spectroscopy measurements, we also find that evolving Ni site speciation during RCC plays a role in deactivation of the catalyst at highly negative potentials, providing previously unexplored insights into the role of catalyst dynamics during RCC in amines.

## Results and Discussion

### Evaluating Dependence of RCC Pathways on Ni–N–C Coordination

To model reaction pathways of RCC on Ni–N–C catalysts, we focused on four-coordinated Ni sites—representing the most stable configuration—and examined a series of NiN_*x*_C_4–*x*_ environments (*x* = 0, 1, 2, 3, 4) (Figures 1a,b and S1), allowing for a systematic evaluation of how the local atomic environment influences catalytic behavior. To evaluate the relative importance of each of these sites, we used experimental Ni K-edge extended X-ray absorption fine structure (EXAFS) measurements to obtain an ensemble coordination number of a representative Ni–N–C catalyst (Figure 1c and Tables S1 and S2). Probing the Ni K-edge EXAFS allows for obtaining an average number of nearest neighbors around Ni sites, with some sensitivity to the identity of the nearest neighbors based on different scattering. We found that the average coordination was NiN_3.3_C_0.7_, suggesting prevalent NiN_3_C_1_ and NiN_4_ sites. Thus, we focus much of our detailed analysis on these two types of sites. Nonetheless, we still examined all possible sites theoretically, as minority sites can sometimes dominate catalytic activity. To consider the influence of proton transfer from amines to the catalyst, we assessed the thermodynamic stability of NiN_*x*_C_4–*x*_ catalyst surfaces under varying hydrogen coverages at reducing potential (pH = p*K*_a_(DEA) = 8.9). Our analysis reveals that hydrogen preferentially adsorbs on adjacent carbon sites rather than nitrogen sites (Figures 1d and S2), with the NiN_4_ configuration having preference toward an unhydrogenated surface. As the potential becomes more negative, hydrogen coverage increases, and at sufficiently reducing conditions, the number of adsorbed hydrogen atoms approaches that of the surrounding C atoms.

**Figure 1:**
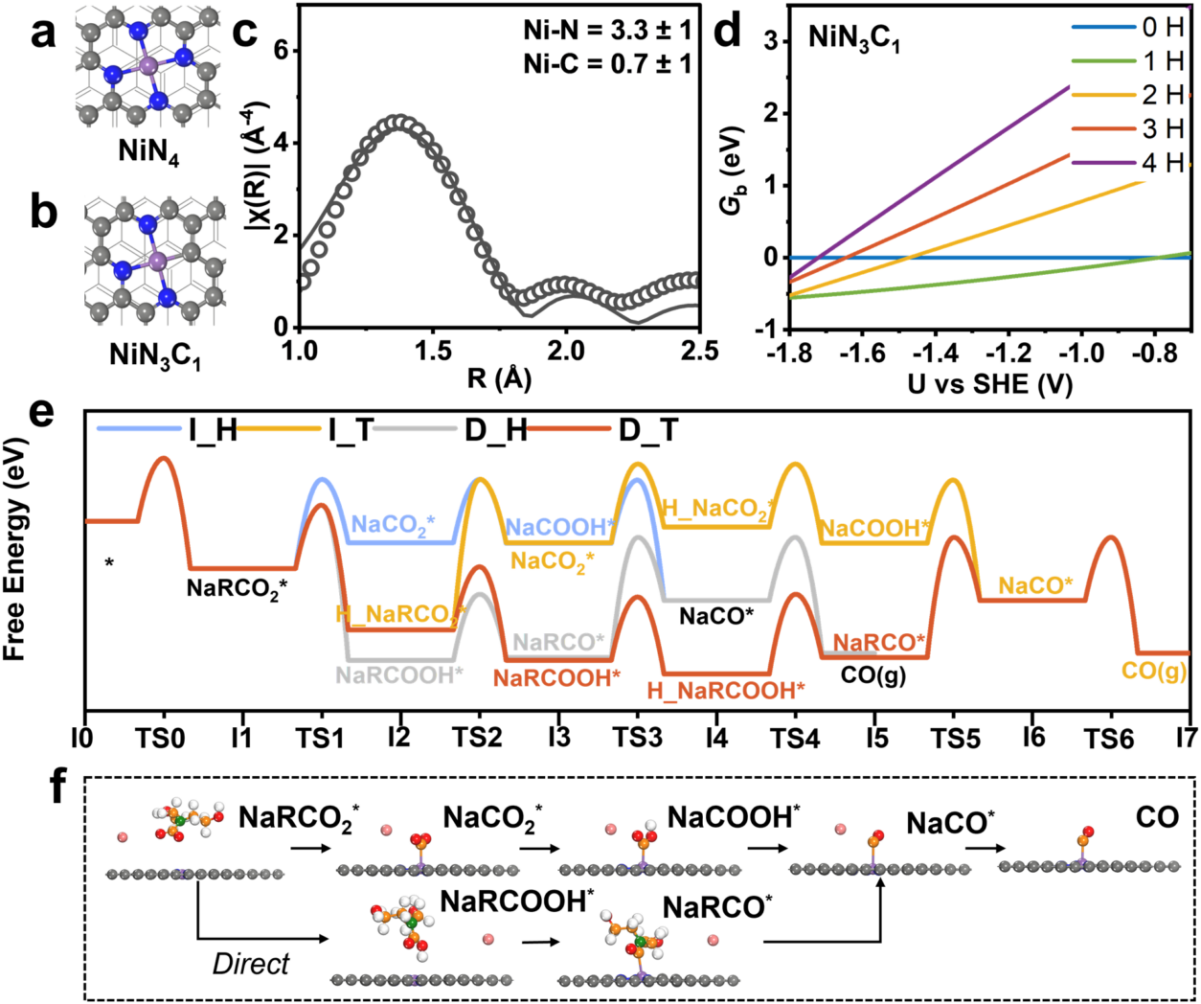
Optimized structures of (a) NiN_4_ and (b) NiN_3_C_1_ obtained that are likely to make up the majority of Ni–N–C sites. Color code of atoms: Ni (purple), N (blue), C (gray), and H (white). (c) Extended X-ray absorption fine structure (EXAFS) data (circles) and fitting (line) of as-synthesized Ni–N–C powder. (d) Thermodynamic stability of NiN_3_C_1_ sites under varying hydrogen coverages. (e) Reaction energy diagram on NiN_3_C_1_ at 0 H coverage and an applied potential of −1.2 V, illustrating four reaction pathways: I_H–Indirect CO_2_ reduction with protons from the solvent (Heyrovsky-type); I_T–Indirect CO_2_ reduction with a surface-adsorbed proton (Tafel-type); D_H–Direct CO_2_ reduction with protons from the solvent (Heyrovsky-type); D_T–Direct CO_2_ reduction with surface-adsorbed H (Tafel-type). In all cases, R denotes the DEA (diethanolamine) group in the captured intermediate NaRCO_2_. Atom color code: Ni (purple), Na (pink), N of the catalyst (blue), N of DEA (green), C of the catalyst (gray), C of DEA (orange), and H (white). (f) Corresponding structures for key intermediates along the RCC pathway with indirect pathway shown on top and the direct pathway shown on the bottom.

To understand the likelihood of RCC proceeding on NiN_*x*_C_4–*x*_ to begin with, we also evaluated its thermodynamic viability against typical CO_2_R and HER. We broadly define RCC as any adsorption of the carbamate to the catalyst surface, regardless of whether reduction is completed while the carbamate C–N bond is intact. Near the electrode surface, where the HER rapidly consumes protons and creates a locally alkaline environment, CO_2_ liberation becomes further suppressed since protonation—a prerequisite for decomposition—is disfavored. Nonetheless, when amine solutions are saturated with CO_2_ there will be free CO_2_ in equilibrium with DEA/carbamate and bicarbonate. While carbamates and bicarbonate nominally have similar expected thermodynamic direct reduction potentials,^13^ there is a lack of evidence in the literature for direct bicarbonate reduction. Instead, researchers have leveraged pH swing release of CO_2_ from bicarbonate for efficient conversion through interfacial acidification.^37,38^ As the present studies are under neutral to alkaline conditions, we consider the thermodynamic competition between the following reactions and not direct bicarbonate reduction

1 $$\mathrm{RCC}:{\mathrm{Na}}^{+}\left(\mathrm{DEA}‐{{\mathrm{CO}}_{2}}^{-}\right)+3\left(\mathrm{DEA}‐H^{+}+{{\mathrm{ClO}}_{4}}^{-}\right)+2e^{-}\to \mathrm{CO}+H_{2}O+4\mathrm{DEA}+{\mathrm{NaClO}}_{4}+2{{\mathrm{ClO}}_{4}}^{-}$$

2 $${\mathrm{CO}}_{2}R:{\mathrm{CO}}_{2}+2e^{-}+2\left(\mathrm{DEA}‐H^{+}+{{\mathrm{ClO}}_{4}}^{-}\right)\to \mathrm{CO}+H_{2}O+2{{\mathrm{ClO}}_{4}}^{-}+2\mathrm{DEA}$$

3 $$\mathrm{HER}:2\left(\mathrm{DEA}‐H^{+}+{{\mathrm{ClO}}_{4}}^{-}\right)+2e^{-}\to 2\mathrm{DEA}+H_{2}+2{{\mathrm{ClO}}_{4}}^{-}$$

We found that the calculated equilibrium potentials (Figure S3) followed the order HER (−0.53 V) < RCC (−0.78 V) < CO_2_R (−1.11 V). This trend demonstrates that RCC is thermodynamically preferred to CO_2_R. In other words, proceeding through a mechanism of reducing CO_2_ facilitated by carbamate adsorption is thermodynamically preferable. However, HER facilitated by preferential adsorption of DEAH^+^ is competitive with RCC, consistent with past observations of low RCC selectivity. Going forward, we focused primarily on RCC and HER for theoretical consideration of the mechanistic pathways on the NiN_4–*x*_C_*x*_.

To understand the influence of the adsorbed hydrogen, we then investigated RCC reaction pathways on various Ni–N–C catalysts under different hydrogen coverages (Figure 1e) as a means of identifying the most likely mechanistic steps of RCC on Ni–N–C catalysts The RCC mechanism can be classified into two main categories based on the sequence of C–N bond cleavage. The first category involves early C–N bond breaking immediately following NaRCO_2_ adsorption, transitioning from RCO_2_ to CO_2_, corresponding to *indirect CO*_2_ *reduction*, where DEA serves as a shuttle of CO_2_ to the electrode. Note that the electrolyte cation, Na^+^, is included in the model, based on its electrostatic coordination to the negatively charged adduct; it assists the transport to the cathode. The second category involves later C–N bond breaking, occurring from RCO to CO corresponding to *direct RCC*, where CO_2_ being reduced while on the adduct. These pathways are illustrated in Scheme 1. We also considered the origin of hydrogen involved in the hydrogenation steps. In a Heyrovsky-type mechanism, protons are donated directly from the solvent (DEAH^+^), whereas in a Tafel-type mechanism, hydrogen is preadsorbed on the catalyst surface prior to the reaction. These two hydrogenation mechanisms, combined with the two C–N cleavage sequences, result in a total of four possible reaction pathways (Figure 1e,f). This classification provides a framework for rationalizing how the bond-breaking sequence and the hydrogen source diversify the RCC mechanism. To estimate reaction barriers, we employ the *energy span model*,^39^ where reactivity is approximated by the largest energy difference between a transition state and an intermediate along the pathway—a process referred to as the rate-determining process (RDP).^13^ The calculated energy spans across different reaction pathways, catalysts, and hydrogen coverages are summarized in Figure S2.

By incorporating the Boltzmann distribution of hydrogen coverages and the corresponding energy span values, we determined the probability distribution of different reaction pathways for catalysts with each speciation (Figures 2a,b and S4). Our analysis reveals that as the potential becomes more negative, surfaces with higher hydrogen coverages contribute more prominently to the overall reaction for active sites that have direct carbon coordination (Figures 2b and S4). Furthermore, the dominant reaction pathway shifts from *I_H* to *D_H* (i.e., *indirect RCC* to *direct RCC* with protons coming from the DEAH^+^), while *D_T* (*direct RCC* with a preadsorbed proton) also contributes to reaction at intermediate potentials.

**Figure 2:**
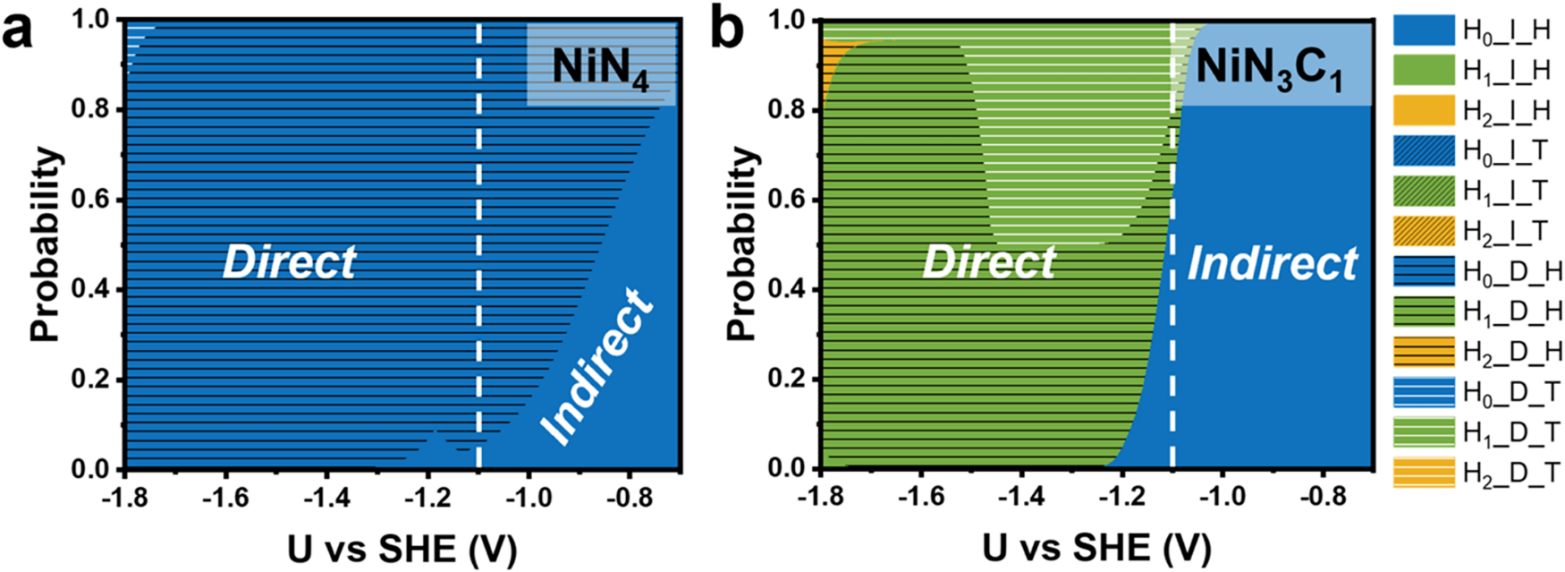
Probability distribution of different RCC pathways on (a) NiN_4_ and (b) NiN_3_C_1_ with varying hydrogen coverages, highlighting their relative contributions to the reaction rate. H*_n_* _I_H represents the I_H pathway on a surface with n adsorbed hydrogen atoms.

To rationalize the potential-dependent activity trends and locate the bottleneck of each reaction pathway, we further analyzed RDP for each pathway under different applied potentials (Figure S5). At lower overpotentials (−1.0 V), the I_H exhibits superior performance, with the rate-determining process primarily involving two key transitions: I1TS2 (RCO_2_* → COOH*) and I1TS1 (RCO_2_* → CO_2_*). However, as the potential becomes more negative (−1.2 V), the energy span for I_H generally increases, shifting the rate-determining process predominantly to I1TS1. This shift occurs because the applied potential preferentially stabilizes RCO_2_ rather than CO_2_. Simultaneously, D_H becomes more favorable, with its RDP transitioning from I2TS3 (RCOOH* → CO*) and I3TS3 (RCO* → CO*) to I2TS2 (RCOOH* → RCO*), as the applied potential significantly enhances the binding energy of CO. Additionally, D_T demonstrates improved performance under more negative potentials, when it shares the same RDP as D_H. These findings provide a basis for tuning key steps to improve catalytic performance.

Next, we calculated the weighted energy spans for NiN*_x_* C_4–*x*_ under different applied potentials (Figure 3a). A lower weighted energy span corresponds to higher catalytic activity. Our results indicate that the activity follows the trend: NiN_1_C_3_ ≈ NiN_2_C_2_ > NiN_3_C_1_, while NiN_4_ and NiC_4_ exhibit poor activity. As the potential decreases, the overall RCC activity increases, though the change in NiN_1_C_3_ remains relatively minor.

**Figure 3:**
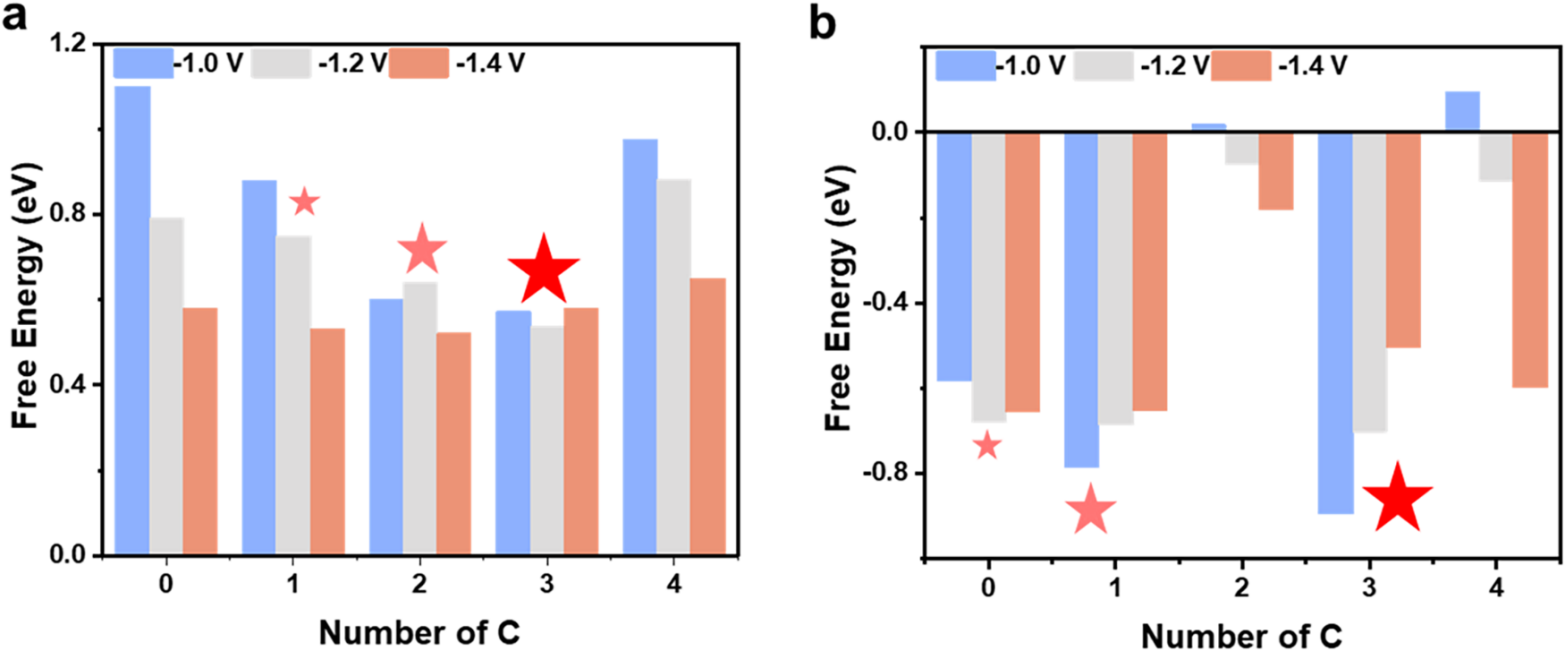
(a) Calculated weighted energetic spans for RCC and (b) the difference in weighted energetic spans between RCC and HER on NiN_*x*_C_4–*x*_ under varying applied voltages. The red stars highlight key data points indicating significant trends in the variations in energetic span.

To assess the viability of RCC in competition with HER, we calculated the energy span of HER on NiN_*x*_C_4–*x*_ catalysts under varying hydrogen coverages and applied potentials (Figure S6), followed by evaluation of the corresponding weighted energy spans (Figure S7). The difference between the weighted energy spans of RCC and HER was then used as a descriptor to quantify selectivity (Figure 3b). A more negative value indicates higher RCC selectivity. Our analysis reveals that NiN_2_C_2_ and NiC_4_ exhibit poor selectivity, while the trend among the other catalysts follows NiN_1_C_3_ > NiN_3_C_1_ > NiN_4_. Notably, out of these three most selective sites, selectivity decreases as the potential becomes more negative except for in the case of NiN_4_. Considering that the Ni–N–C sites are likely to have a nitrogen coordination number close to 4,^40^ we expect NiN_1_C_3_ to be a minority compared to NiN_3_C_1_, and yet could hypothetically dominate the RCC activity.

### Experimental RCC Selectivity Dependence on Potential

To test our predictions about the behavior of Ni–N–C under RCC conditions, we first synthesized Ni–N–C according to a previously established procedure^28^ with slight modifications, using a silica template to obtain a porous Ni–N–C catalyst (see Experimental Section for details). To evaluate the viability of RCC on Ni-SACs against previous examples of pure metals, we utilized a sealed rotating disk electrode (RDE) cell, as described elsewhere,^15,41^ to benchmark against pure metal catalysts measured in the same configuration. Initially, to prove successful synthesis of a catalyst producing a high ratio of CO/H_2_ from CO_2_ electroreduction, we conducted a CO_2_R benchmark on the Ni–N–C on glassy carbon in 1 M NaHCO_3_ (Figure 4a). As expected, there is a high selectivity for CO compared to H_2_, with Faradaic efficiency (FE) at −12 mA cm^–2^ exceeding 80%.

**Figure 4:**
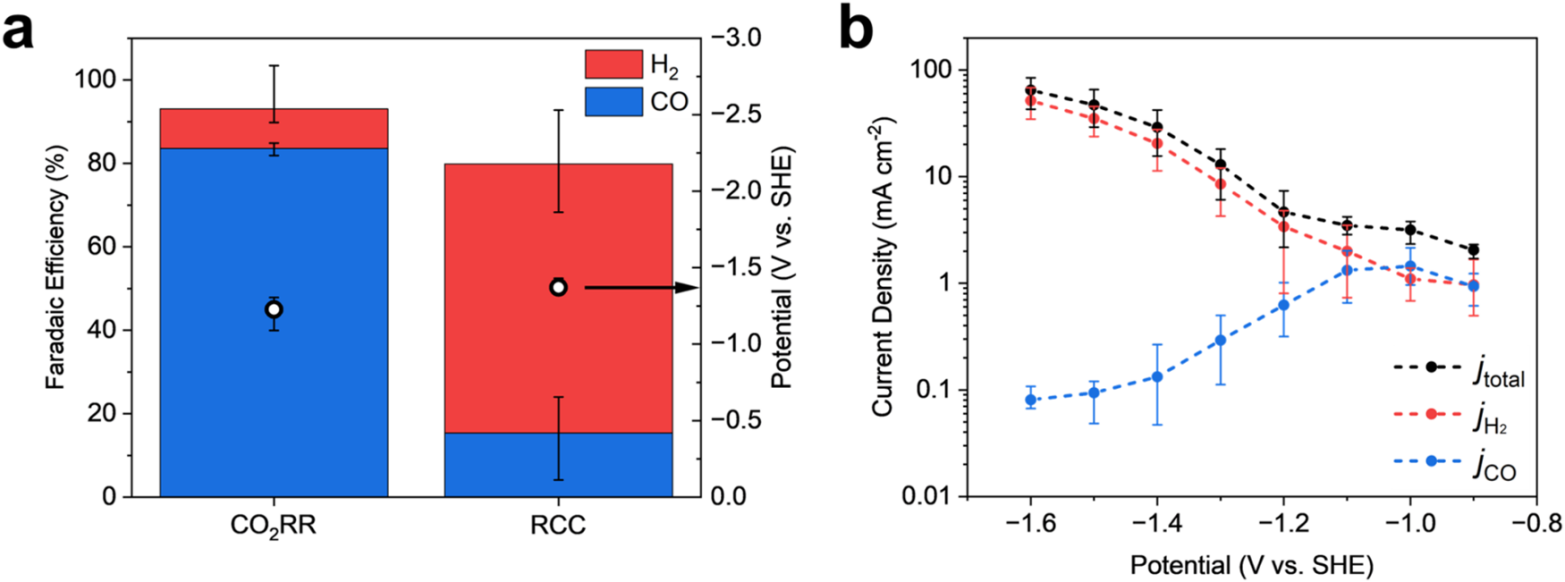
Kinetics of RCC with Ni–N–C catalyst measured in a sealed rotating disk electrode (RDE) cell under rotation at 800 rpm–error bars represent the minimum and maximum values over three parallel tests. (a) Comparison of Faradaic efficiency (bars, left axis) and operating potential (dots, right axis) under constant current for conventional CO_2_R (flowing CO_2_ into 1 M NaHCO_3_ solution) and RCC (flowing Ar into 0.5 M DEA/0.5 M NaClO_4_ solution that was prepurged with CO_2_), and (b) potential dependence of CO selectivity for RCC on Ni–N–C catalyst under RCC conditions (flowing Ar into 0.5 M DEA/0.5 M NaClO_4_ solution that was prepurged with CO_2_).

For a general evaluation of whether there would be detectable CO from RCC, we then measured the same dropcasted RDE in a solution of 0.5 M DEA and 0.5 M NaClO_4_ that was presaturated with CO_2_. During the measurement, the solution was purged with Ar. We observed an average of 15% FE for CO under these conditions (Figure 4a). Each parallel test represents an average over three samplings in a 1 h test in which the CO FE would decrease over the course of the measurement to as low as 1%. However, the observation of CO under RCC conditions at −12 mA cm^–2^ was promising, as metal cylinders of Cu, Sn, Au, and Ag measured at an identical current density in the same cell showed <0.02% FE.^15^

After the initial demonstration of RCC on the Ni–N–C catalyst, we sought to understand how the performance varied across different applied potentials to correlate with our analysis of the mechanisms and energetics. We measured the activity and selectivity dependence of the Ni–N–C catalyst on the RDE while stepping from −0.9 V vs SHE to −1.6 V vs SHE (Figures 4b and S8). Low overpotentials (−0.9 V, −1 V, −1.1 V vs SHE) had moderate CO selectivity, with nearly 1:1 CO:H_2_. At −1.2 V vs SHE there is a noticeable drop in CO selectivity and increase in H_2_ selectivity (Figures 4b and S8). As the potential becomes more and more negative past −1.2 V vs SHE, the total current density continuously increases and HER becomes dominant. At a potential of −1.6 V vs SHE, the CO FE decreases to <1%. The observation of peak selectivity between −0.9 and −1.1 V vs SHE is consistent with a similar measurement of 1 M DEA, which had peak selectivity for CO between approximately −0.96 V and −1.16 V vs SHE (assuming pH = 8 for 1 M DEA).^32^ However, a slightly lower overall FE_CO_ was observed, warranting further investigation into the experimental factors that contribute to differences in RCC selectivity for a given catalyst.

The shift in selectivity correlates with the increasing difference in the weighted energy spans of RCC and HER on NiN_3_C_1_ sites (Figure 3b), indicating a growing preference for HER. This also implies a large contribution of NiN_3_C_1_ sites to RCC activity, as opposed to NiN_4_ sites. Prevalent NiN_3_C_1_ would also explain the higher RCC activity at low overpotentials, since NiN_4_ is predicted to be less active at low overpotentials (Figure 3a). Thus, we would expect a significant onset of the direct mechanism being the primary mechanism on NiN_3_C_1_ around −1.1 V vs SHE, near the peak of the observed CO selectivity. Instead, we observe very little RCC at potentials more negative than −1.1 V vs SHE. We assume that there are mass transport limitations that promote HER in favor of RCC, although this does not rule out the possibility of the small amount of RCC being through the direct mechanism. The deviation from the results of our energy span calculations at larger overpotentials led us to further explore the speciation of the microenvironment that could present these unforeseen limitations at the electrode surface.

We then conducted vapor–liquid equilibrium (VLE) modeling (Figure S9) to better understand the nature of the evolving bulk solution speciation that results in dynamic RCC performance. The VLE models indicate that as CO_2_ loading of the amine decreases, the availability of free CO_2_ rapidly decreases away from the saturation point. Carbamates increase as CO_2_ decreases at lower loadings, but eventually peak and decrease in favor of DEA (without bound CO_2_) at very low CO_2_ loading. At every nominal DEA concentration, the most concentrated species is DEAH^+^, closely followed by bicarbonate. Given that DEAH^+^ and bicarbonate also have similar acidity (with p*K*_a_ values of 8.9 and 10.3, respectively) these are both likely proton donors for RCC and HER, with DEAH^+^ most strongly promoting HER. While bicarbonate may serve as a proton donor or indirect source of carbamate/free CO_2_, we do not expect it to be feasible to directly convert. Instead, at more positive potentials, the moderate CO selectivity likely arises through thermodynamic preference for indirect RCC over HER in addition to a moderate availability of carbamates and free/unbound CO_2_ at the interface–with each being roughly similar concentrations in solution. At more negative potentials, higher current density will also drive a locally alkaline pH that will limit both free CO_2_ and carbamates at the interface. As these species become diffusion limited at the interface with increasing current density, there is still an excess of DEAH^+^, so HER is promoted. Thus, RCC on Ni–N–C catalysts is limited to lower overpotentials by a combination of the intrinsic thermodynamics of RCC along with kinetic limitations imposed by the sorbent.

Experimental evidence also shows that in sequential or long-term testing, the partial pressures of CO_2_ in the headspace also vary when purging with inert gas (Figure S10). Assuming CO_2_R is also in competition with RCC at some potentials, this also leads to ambiguity of the intrinsic behavior of Ni–N–C catalysts under RCC conditions. The slightly more positive potential for RCC at the same current density (Figure 4a) is likely also accounted for by the decreasing CO_2_ partial pressure causing an accompanying pH increase, which is indicative of CO_2_ loading decreasing over time (Figure S10). The presence of dissolved (free) CO_2_ may compete with the indirect RCC (with surface bound CO_2_ adsorbates) and makes assignment of the CO selective potential region ambiguous (Scheme 2). Variability in dissolved CO_2_ may also account for the wider variability of RCC measurements compared to CO_2_R. This motivates further systematic study with control over the concentration of the sorbent and the partial pressure of free CO_2_ to decouple these contributions.

**Scheme 2:**
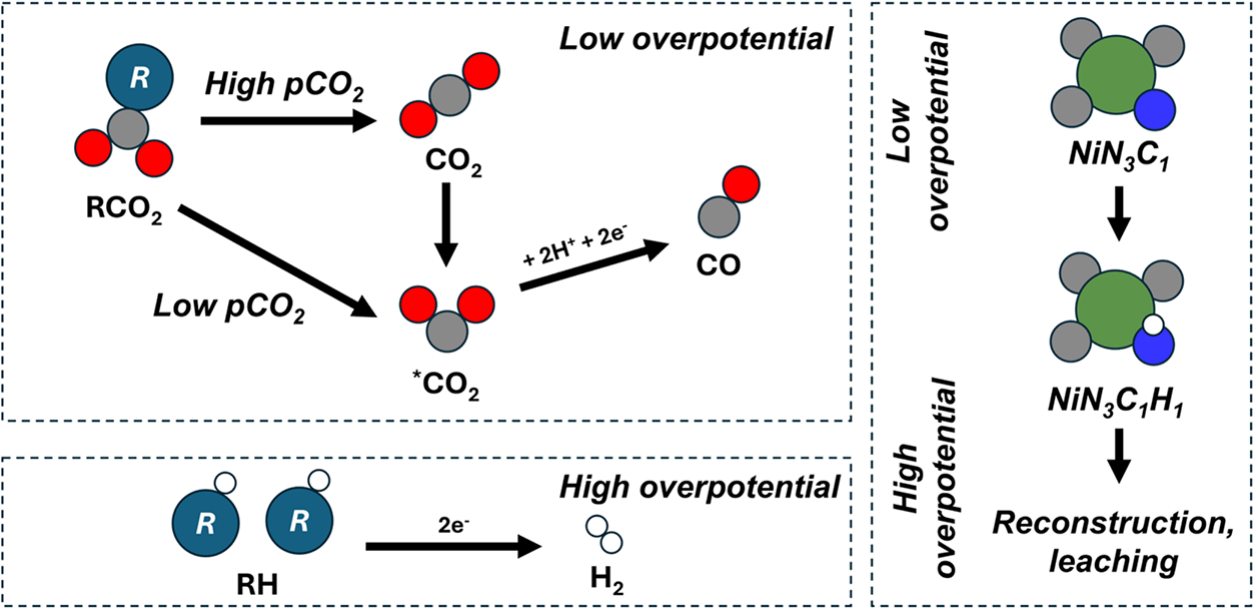
Proposed Competing Mechanisms at Low Overpotential (Top Left), the HER Mechanism at High Overpotential (Bottom Left) and the Primary Speciation of the Ni Site Evolving as Overpotential Increases (Right)^a^

a Gray = carbon, red = oxygen, white = hydrogen, green = nickel, blue = nitrogen.

### RCC Selectivity Dependence on Sorbent and CO_2_ Concentration

To better understand the kinetic limitations on RCC as a result of solution speciation, we then utilized a two-compartment flow cell as a platform to evaluate the ideal conditions for RCC operation with Ni-SACs. The flow cell allowed high flux of captured CO_2_ to the reactive interface, and use of an electrolyte reservoir separate from the cell allowed for quick swapping of electrolyte solutions that were prepurged with CO_2_. In order to understand the role of free CO_2_, we varied partial pressures of CO_2_ during the electrochemical tests (after fully saturating the solutions with CO_2_ beforehand). We conducted typical RCC measurements under pure Ar gas and pure CO_2_ gas, as well as mixed 10% and 25% CO_2_ gas streams that are relevant to CO_2_ point sources such as flue gas. With each of these partial pressures of CO_2_, we measured different concentrations of DEA. We maintained a 1:1 concentration of NaClO_4_:DEA for all solutions, as Na^+^ plays a role in stabilizing surface intermediates and transporting reactants, which may be particularly important for the adsorption of carbamate. To interpret these results, we also used in-line pH monitoring (Figure S11) and the VLE models (Figure S9) to understand the evolving speciation under varied partial pressures of CO_2_. We conducted each test at a constant potential of −1.1 V versus the standard hydrogen electrode (SHE). We used the SHE scale, rather than the reversible hydrogen electrode (RHE) scale due to the gradual increase in pH of tests under dilute CO_2_ streams which leads to difficulty in setting a truly constant RHE potential.

For each CO_2_ stream, increasing the concentration of DEA from 0.1 to 0.5 M led to a higher *j*_CO_ (Figure 5a). The partial current density for HER increased with increasing sorbent concentration (Figure 5b) due to higher concentrations of the corresponding DEAH^+^ (Figure S9) as sorbent concentration was increased from 0.1 to 1 M DEA. We also studied the effect of CO_2_ partial pressure in the incoming gas stream, using different mixtures of Ar and CO_2_. Each increase in CO_2_ partial pressure led to an increase in *j*_CO_ at 0.1 and 0.5 M concentrations of DEA (Figure 5c). HER activity, on the other hand, was relatively flat with 0.1 M NaHCO_3_, 0.1 M DEA and 1 M DEA across different CO_2_ partial pressures (Figure 5d). The HER activity does not strongly depend on the CO_2_ partial pressure, likely since it does not significantly perturb the concentrations of relevant HER proton donors (HCO_3_^–^ and DEAH^+^) in these systems as evidenced by the VLE models (Figure S9).

**Figure 5:**
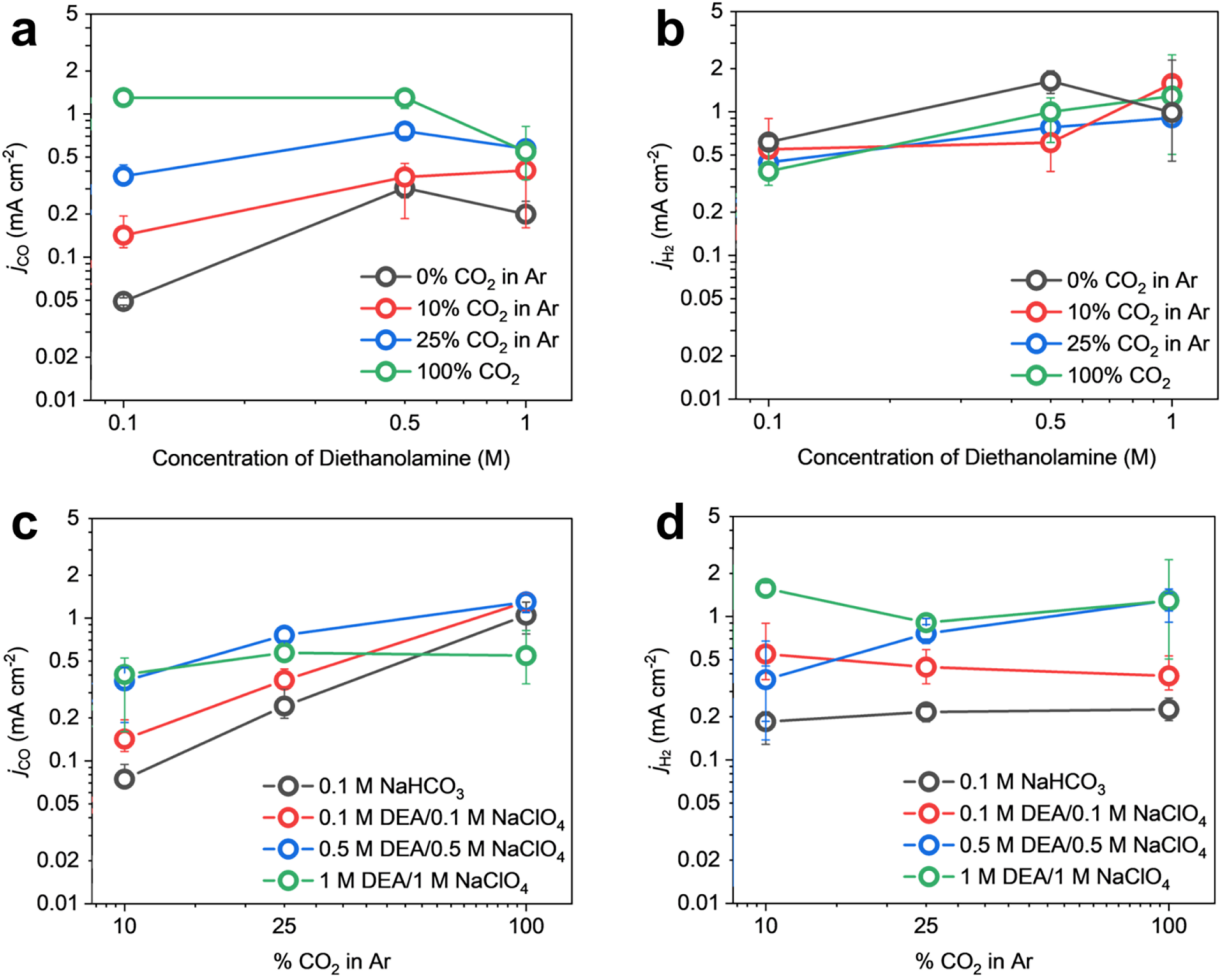
Evaluation of partial current density of CO at a constant potential of −1.1 V vs SHE using Ni–N–C catalyst deposited on carbon paper and measured in a flow cell, varying (a) *j*_CO_ and (b) *j*_H_2__ while varying concentration of the diethanolamine sorbent (with 1:1 concentration of NaClO_4_) and (c) *j*_CO_ and (d) *j*_H_2__ while varying partial pressure of the incoming CO_2_ stream. Positive and negative error bars represent the maximum and minimum value, respectively, from at least two parallel tests.

While there are relatively small differences in starting pH (∼7) between 0.5 M DEA and 1 M DEA (Figure S11), both indicate approximately 1:1 amine:CO_2_ loading as evidenced by the VLE modeling (Figure S9). At the saturation point, there is an increase in the amount of CO_2_ in equilibrium with the capture solution. This explains the exponential activity increase between 0.1 and 0.5 M DEA under 0% CO_2_, since despite their increase in pH over time they also begin at the CO_2_ saturation point. With increased CO_2_ partial pressure, there is a commensurate increase in *j*_CO_. When the concentration of DEA is increased to 1 M, there are either diminishing returns or decreases in *j*_CO_ (Figure 5a). With 100% CO_2_, there is also a noticeable drop in *j*_CO_ between 0.5 and 1 M DEA (Figure 5c). With 1 M DEA, there is very little free DEA compared to CO_2_ and carbamate, which are roughly equal in concentration (Figure S9f). With 0.1 M DEA and 0.5 M DEA, the free DEA concentration is approximately equal to (or greater than) the carbamate and free CO_2_ concentration near the saturation point. The lack of available DEA may impede turnover of CO_2_ to carbamate and subsequent adsorption and conversion of carbamates. This situation is different from previous observations of free DEA at the interface impeding CO selectivity on Ag catalysts, due to Ag catalysts primarily reducing free CO_2_, rather than carbamates at mild overpotentials.^14,16^ Assuming CO_2_ is a less likely adsorbate than carbamate, changes in the rate of CO production at different CO_2_ partial pressures then imply that the CO_2_ mainly serves to maintain favorable concentrations of DEA, carbamates, and free CO_2_ in solution to efficiently deliver carbamates to the interface. Increasing the amount of DEA (at low concentration, 0.1 to 0.5 M DEA) is consistent with increasing activity due to higher carbamate concentration, suggesting RCC is prevalent, as predicted (Figure 3). At higher amine concentration (1 M DEA), the concomitant decrease in activity and free DEA concentration relative to carbamates suggests that Ni–N–C catalysts exhibit different behavior from metal catalysts that do not directly reduce carbamates. The FEs of Ni–N–C catalysts measured at −1.1 V vs SHE under varied CO_2_ partial pressures and DEA concentrations are summarized in Figure S12. Under Ar purging we observed increasing FE with DEA concentration, up to 20% with 1 M DEA. This is a clear improvement over RCC on pure metals. Ag catalysts have been the most studied for HER, and under inert gas purging typically have FEs < 10%,^14,15^ especially without the use of larger cations.^18^ Additionally, the use of 25% CO_2_ concentration can improve the FE of Ni–N–C catalysts up to 45%, whereas Ag catalysts have been observed to be less selective even under more concentrated CO_2_ streams.^16^

Notably, the results under Ar saturation have CO_2_ concentrations that are less than 10% CO_2_ in the headspace that generally decrease throughout each measurement and have consequent changes in *j*_CO_/CO FE. These changes correlate with the drop in headspace CO_2_ except for in the case of the 0.1 M NaHCO_3_ electrolyte (Figure S13). NaHCO_3_ has the lowest headspace CO_2_ concentration (<1%) at the start of the test, and although there is a gradual decrease in headspace CO_2_, the apparent CO FE does not decrease. We also note that 0.1 M NaHCO_3_ consistently had lower activity than DEA containing solutions under dilute streams of CO_2_ (Figure S12). This is despite 0.1 M DEA only having slightly <0.1 M bicarbonate under CO_2_ saturated conditions. The pronounced effect on *j*_CO_/CO FE, especially under Ar-purged conditions, suggests that the minority DEA/carbamate species play a much more pronounced role in the observed RCC activity, as expected. This is likely a consequence of slow ambient CO_2_ release and the intrinsic kinetic difficulty of reducing bicarbonate directly.

While most tests were approximately one hour, we also conducted several longer tests with 1 M DEA to confirm consistency due to unaccounted charge (i.e., < 100% total FE). We found that CO FE stayed consistent at ∼40% under CO_2_-saturation (Figure S14a) due to consistent speciation, as evidenced by the unchanged pH (7.3) and consistent gas environment. Under Ar saturation, the CO FE slowly dropped due to a gradual decrease in headspace CO_2_ (5% to 3%) and increase in pH (7.3 to 7.8). For both cases, the H_2_ FE increased gradually over the course of two hours, with the total FE being ∼60% upon the first GC injection and nearly 90% for the last several injections (Figure S14b). While the trends for *j*_CO_ track the CO FEs (Figure S14c), *j*_H_2__ is relatively consistent in both cases (Figure S14d). This leads us to believe that unaccounted charge mainly results from charging currents, which may be difficult to dissipate due to the combined porosity of the Ni–N–C catalyst and carbon paper substrate. The full results of the three >2 h tests are shown in Figure S15.

### *In Situ* Investigation of Ni–N–C Active Site Structures under Operating Conditions

Although the changing concentrations of species near the interface drive decreases in CO selectivity at more negative potentials/current densities, we also sought to understand the importance of catalyst active site evolution during RCC and the extent to which it led to decreases in selectivity. When going to potentials more negative than −1.1 V vs SHE, large drops in CO selectivity were consistently observed (Figure 6a). After applying potentials where predominantly HER was observed, the selectivity could not be recovered by sweeping the potential back to potentials where the catalyst was previously CO selective. This suggests that there may be irreversible changes to the catalyst structure. To probe the structure around Ni sites (i.e., coordination within the carbon framework and adsorbates) as it evolved during immersion in DEA and under applied potential, we used *in situ* X-ray absorption spectroscopy (XAS). The *in situ* XAS cell mimicked the flow cell setup used for benchmarking, with an additional beveled opening in the backing plate near the cathode to allow incident X-rays through to the catalyst film (Figure S16).

**Figure 6:**
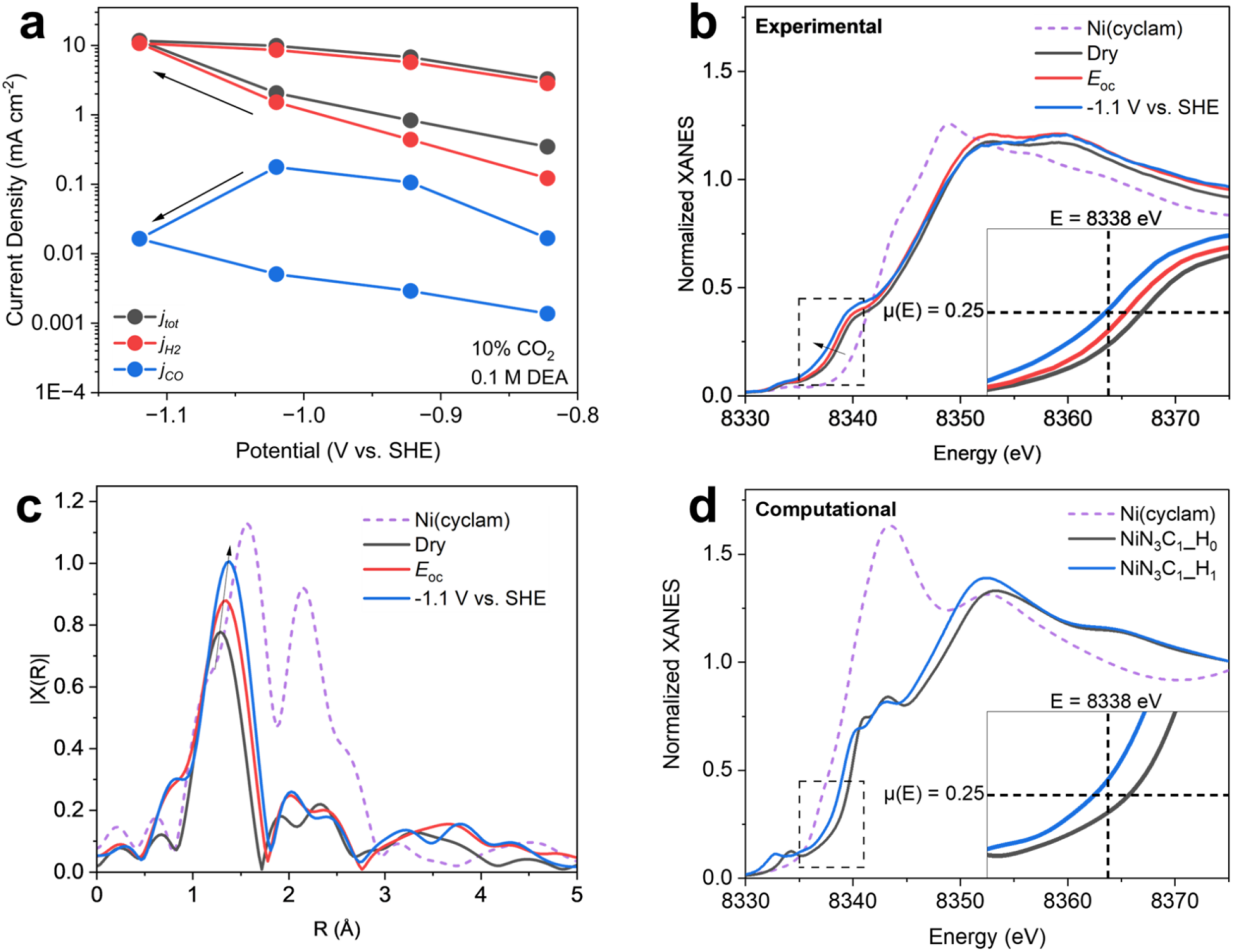
Evidence of the Ni–N–C catalyst restructuring assessment under RCC conditions. (a) Constant potential measurements of Ni–N–C under RCC conditions (0.1 M DEA/0.1 M NaClO_4_ with 10% CO_2_ supply). (b) Ni K-edge XANES of a dry Ni–N–C on carbon paper electrode compared to an electrode immersed at open circuit (*E*_oc_) in CO_2_-purged 1 M DEA/1 M NaClO_4_ and an electrode in the same solution at −1.1 V vs SHE, with (c) corresponding *k* ^*2*^-weighted EXAFS spectra, and (d) simulated XANES spectra of NiN_3_C_1_ with (NiN_3_C_1__H_1_) and without (NiN_3_C_1__H_0_) adsorbed hydrogen on the coordinating carbon, with the simulated spectrum of 4-fold coordinated Ni–N complex Ni­(cyclam) shown for comparison.

The X-ray near edge structure (XANES) spectra shows a shift of the absorption edge of the Ni–N–C samples to lower energy upon immersion of the electrode into a CO_2_-saturated 1 M DEA solution, and a subsequent shift to even lower energy under an applied potential of −1.1 V vs SHE (Figure 6b). Notably, some previous studies of conventional CO_2_R on Ni–N–C catalysts observe similar shifts in the absorption edge under CO_2_R conditions,^42–45^ although typically CO_2_ adsorption under open circuit conditions shows a positive shift in the adsorption edge. We hypothesize that under working conditions where indirect RCC is dominant, the primary adsorbate’s CO_2_ interactions with the catalyst will be similar to conventional CO_2_R, while under open circuit conditions interactions with the sorbent may be more prevalent. The extended X-ray absorption fine structure (EXAFS) of the Ni–N–C catalysts under the same conditions indicates a slight bond lengthening and increase in coordination in the first shell coordination of N/C around Ni (Figure 6c). Since the bond lengths and scattering factors of nitrogen and carbon atoms will be similar, this could indicate restructuring of the N/C coordination around the Ni or adsorption of the sorbent/CO_2_. This observation also contrasts some other EXAFS measurements of Ni–N–C catalysts measured *in situ* for CO_2_R,^42,44,45^ which warrants further investigation to clarify whether these observations represent restructuring around the Ni within the graphitic carbon plane, or out of plane with the sorbent.

To better understand the coordination structure based on the experimental results, we simulated the XANES spectra for likely coordination structures of the Ni–N–C. We simulated the XANES spectra of NiN_3_C_1_ (Figure 6d) and NiN_1_C_3_ (Figure S17) under both dry conditions and working conditions at −1.2 V, where the active surface is characterized by the adsorption of a single hydrogen atom for NiN_3_C_1_. Our experimental results showed that under working conditions, the absorption edge shifts toward lower energy compared to the dry state, indicating a partially lower Ni oxidation state (Figure 6b). For the simulation of NiN_3_C_1_, we observed a similar absorption edge shift (∼0.8 eV) to lower energy upon adsorption of a hydrogen on the coordinating carbon (Figure 6d). A similar trend is observed in NiN_1_C_3_ (Figure S18). The similarity in the absorption edge shift near μ­(*E*) = 0.25 between the experimental and simulated results suggest that protonation (at least partially) drives changes to the local structure of the catalyst active sites and further supports the prevalence of NiN_3_C_1_ sites in the as-synthesized catalyst. More complex features in the XANES, as well as the changes at open circuit, are likely explained by the effects of the primary adsorbates (*vide supra*). These results suggest that protonation changes the local structure of the Ni–N–C active sites under working conditions and thus, protonation at the active site is consequential for RCC activity.

To further investigate the impacts of protonation on NiN_*x*_C_4–*x*_ catalysts and identify possible origins of restructuring, we used theoretical calculations to probe the nature of the restructuring under initial formation of the NiN_*x*_C_4–*x*_ sites and under working RCC conditions. To assess the thermodynamic stability of SACs, we calculated the relative formation energy of NiN_*x*_C_4–*x*_, as shown in Figure 7a. The results indicate that formation energy increases markedly with decreasing nitrogen coordination, suggesting that structures with higher nitrogen coordination are more thermodynamically stable, consistent with experimental observations of prevalent Ni–N_4_ and Ni–N_3_C_1_ sites.

**Figure 7:**
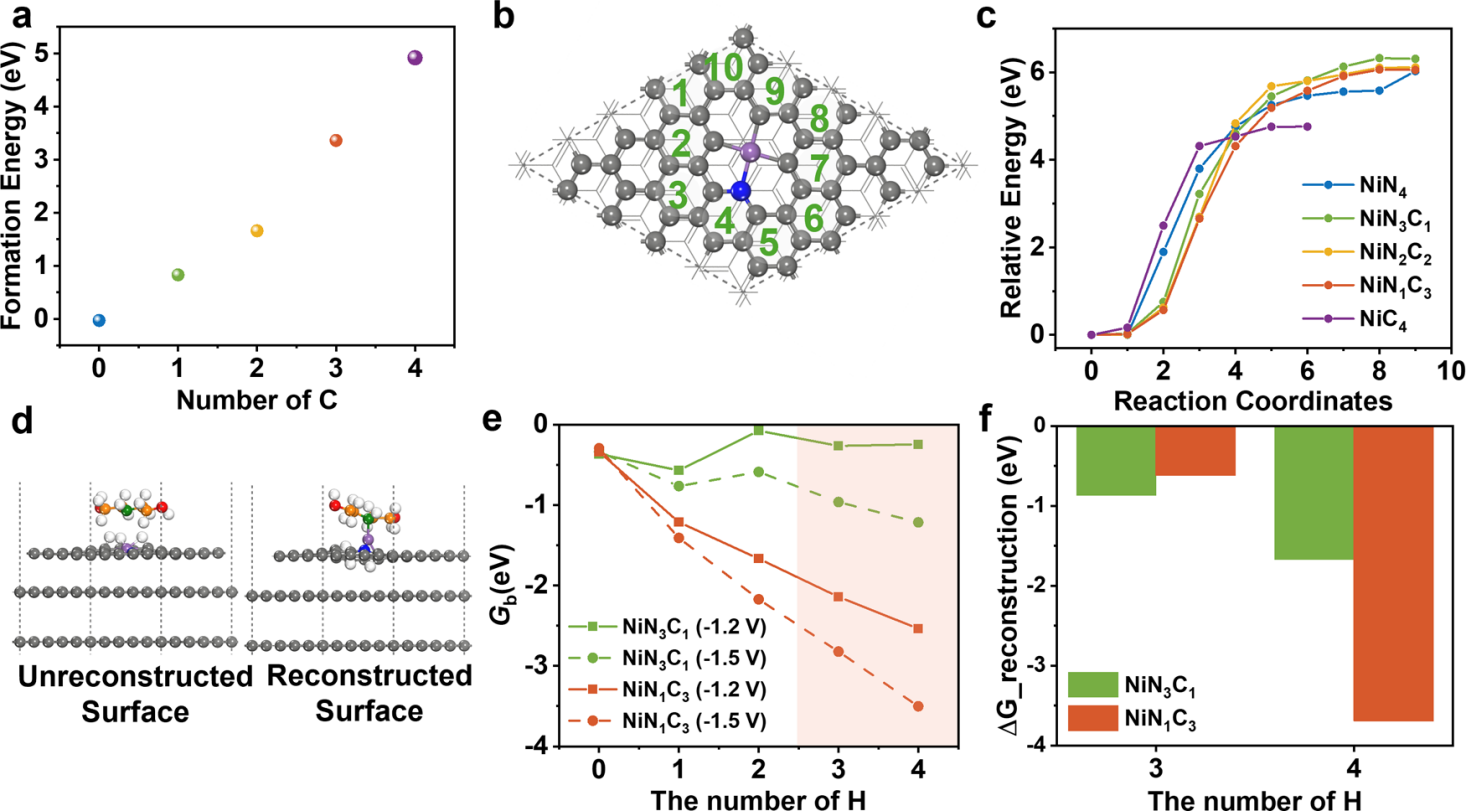
(a) Formation energies of NiN_*x*_C_4–*x*_ structures. (b) Ten potential Ni transfer sites. (c) Energy profile along the reaction pathway for Ni diffusion from original site to an adjacent hollow site. (d) Side view of the optimized DEA adsorption on NiN_1_C_3__H_3_, comparing the unreconstructed and reconstructed structures. (e) Gibbs free energy for coadsorption of DEA and H on NiN_*x*_C_4–*x*_ surfaces under different applied potentials. The binding energy was computed as *G*_b_ = *G*_total(NiN*_x_* C_4–*x*_ + DEA + H)_ – *G*_NiN_x_C_4–x__ – *G*_DEA_ – *G*_H_, representing the overall stability of the DEA + H coadsorbed states. (f) Energy difference between the unreconstructed and reconstructed surfaces at −1.5 V.

To further assess dynamic stability of NiN_*x*_C_4‑x_ catalysts, we analyzed the diffusion barrier of Ni atoms migrating from their original sites to adjacent hollow sites. We designed ten possible migration paths (Figure 7b) and identified the most thermodynamically stable final structures by comparing the relative energies between the initial and final states. These structures were then used to determine the Ni diffusion barrier. Our calculations reveal that, at least without the assistance of ligands from solution, such as the amine, the aggregation of dispersed Ni single atoms is unlikely to occur, as the diffusion barrier exceeds 4 eV for all catalysts. While ligand assistance likely will bring the barrier down, we can use these results to assess the relative stabilities of NiN_*x*_C_4–*x*_ sites. NiC_4_ appears to be less stable compared to other catalysts (Figure 7c), but this structure is an insignificant fraction (*vide supra*). This likely rules out Ni plating as a source of the shift of the XANES absorption edge to the left.

Under operating conditions, metal sites may undergo chemical corrosion due to interactions with DEA that leads to Ni leaching. We examined two adsorption configurations of DEA: (1) Physisorption, where DEA interacts weakly with the surface, and (2) Chemisorption, where DEA forms a chemical bond with Ni, potentially leading to metal leaching (Figure 6d). To probe the driving force for surface restructuring, we evaluated the binding energies of DEA and H on NiN_3_C_1_ and NiN_1_C_3_ under different applied potentials. The red region in Figure 7e marks the potential range where the reconstructed surface becomes more stable. Our results show that as the potential becomes more negative, the increasing hydrogen coverage leads to more negative binding energies of DEA, suggesting a higher tendency for surface reconstruction and leaching. Comparing NiN_1_C_3_ and NiN_3_C_1_, we find that NiN_3_C_1_ is more resistant to Ni leaching, as indicated by its less negative binding energy and smaller energy difference between the reconstructed and unreconstructed surfaces (Figure 7f). This trend suggests that, as the number of carbon atoms increases, the tendency for Ni leaching also increases, highlighting the importance of nitrogen coordination in stabilizing single-atom Ni sites. This is consistent with observations of deactivation at more negative potentials (Figure 6a) and Ni leaching into solution under more negative potentials and over extended periods of time, as evidenced by inductively coupled plasma mass spectrometry (ICP-MS) of the electrolyte after electrolysis (Figure S18). These structural changes likely decrease the number of active sites, therefore deactivating the catalyst. The HER activity at high overpotentials likely then at least partially originates from HER on the graphitic carbon support. We have also summarized the expected changes to the primary Ni–N–C site speciation in Scheme 2.

Considering catalytic activity, selectivity, and stability, we propose that NiN_3_C_1_ sites represent the dominant contribution to RCC performance. Therefore, to further verify the reliability of DFT in describing such systems with complex electronic structures and competing spin states,^46–51^ we performed a benchmark calculation using a quantum embedding approach that performs correlated electronic structure calculations on embedded fragments,^52,53^ including the NiN_3_C_1_H_1_ active site and its graphitic surroundings (Figure S19). More details can be found in the Supporting Information. The DFT-computed spin states are consistent with results obtained from high-level wave function-based methods such as CASPT2 and CCSD­(T), which supports the reliability of DFT for describing the ground state of this system and further validating the accuracy of our computational results.

## Conclusions

This work brings attention to the need for codesign of catalysts, sorbents, and operating conditions to achieve optimal RCC activity. We found that by using a catalyst that can suppress HER, RCC is possible at low overpotentials. Carbon-based products are obtained through generating CO_2_ adsorbates at operating potentials where RCC is kinetically viable. Computational analysis initially predicted RCC through both direct (carbamate reduction) and indirect (C–N bond breaking followed by reduction of adsorbed-CO_2_) pathways. We mainly experimentally observed RCC selectivity at potentials where indirect RCC should be dominant, and experimental evidence also suggests a role of free CO_2_ in equilibrium with the sorbent. To demonstrate this, we experimentally studied different concentrations of CO_2_ constantly fed into the system. Although there is not strong experimental evidence for the direct electroreduction of carbamates (i.e., without an initial C–N bond breaking step) on Ni–N–C catalysts, the presence of a sorbent (DEA) can nonetheless enhance the rate of CO production (compared to a solution only containing sodium bicarbonate) when a dilute CO_2_ source is present. This enhancement is especially pronounced without an external source of CO_2_, where bicarbonate solutions are ineffective as a CO_2_ source to the catalyst, and results in a 19-fold enhancement (evidenced by the partial current density) in the rate of CO production in solutions of 0.5 M DEA. There is also a 3-fold enhancement in the rate at 10 and 25% concentrations of CO_2_, which are most relevant for implementation of RCC from industrial sources. These rates are achievable at lower overpotentials than observed for Ag based catalysts, which have been observed to have a significantly RCC onset for CO at potentials, as well as significantly lower selectivity for CO. Mechanistically, these enhancements are also significant, as they point to the unique ability of Ni–N–C catalysts to proceed through an RCC mechanism, rather than just proceeding through CO_2_R in the presence of amines. While open circuit corrosion of metal catalysts for RCC was previously documented, previous works on Ni–N–C catalysts for RCC have not documented the deactivation of the catalyst resulting from restructuring and leaching. These insights will be important for further developing stable single atom active sites for RCC. To enable enhancements from RCC, the potential must be such that the Ni–N–C sites do not restructure and the supply of carbamates/CO_2_ near the interface is not depleted too rapidly. Our insights into the importance of the RCC microenvironment as well as the corresponding catalyst stability highlight the importance of codesigning catalysts and sorbents, as well as tailoring concentrations of sorbent to the available stream of CO_2_.

## Experimental Section

### Computational Methods

All plane-wave density functional theory (DFT) calculations were performed using the Vienna Ab Initio Simulation Package (VASP),^54,55^ employing the Perdew–Burke–Ernzerhof (PBE) functional within the generalized gradient approximation (GGA) framework.^56^ Projector-augmented-wave pseudopotentials (PAW) were employed with a plane-wave energy cutoff of 400 eV. The model systems were constructed using a *p*(5 × 5) unit cell with three atomic layers. The bottom two layers of the slab were fixed to mimic the bulk, while the top layer and all adsorbates were fully relaxed to represent the interfacial region. A vacuum space of 20 Å was added along the *z*-direction to eliminate interactions between periodic images. For solvation and surface charging calculations, all structures were symmetrized and the simulation cell was extended to 60 Å in the *z*-direction to accommodate the implicit solvation region. Geometry optimizations were considered converged when reaching a gradient smaller than 0.05 eV/Å, with a threshold of 1 × 10^–5^ eV for wave function calculation. Γ-point (1 × 1 × 1) *K*-point mesh was used for all geometry optimizations. Gibbs free energies for adsorbates are obtained from the DFT energies with ZPE and entropy corrections determined from frequency calculations performed for all the considered structures using the Harmonic Oscillator approximation. The grand canonical free energy was evaluated by the grand canonical density functional theory (GCDFT) calculations, which is a surface charging technique. Details can be found in the Supporting Information and our previous article.^15,57,58^ Details of high-level embedding ab initio calculations are given in the SI.

### Catalyst Synthesis

The catalyst synthesis was adapted from literature.^28^ Briefly, 2.5 g of amorphous silica nanoparticles (Thermo Scientific, 99.9%) were suspended in nanopure water, sonicated, and then heated at 1000 °C in a crucible for one hour. Then, 50 mg of nickel chloride hexahydrate was dissolved in 2.5 mL 1-ethyl-3-methylimidazolium (EMIM-DCA) and added to the crucible with the annealed silica. The mixture was then placed back in a furnace and heated at 900–1000 °C for three hours. After cooling, the silica templates were etched away by etching three times with 2 M NaOH at 80 °C for three hours, and then a final etch with 1 M HCl in ethanol. The final product was collected by centrifugation.

### Electrolyte Preparation

Electrolyte solutions for RCC were prepared by 1:1 molar amounts of sodium perchlorate (Sigma-Aldrich, ≥98.0%) and diethanolamine (Sigma-Aldrich, ≥99.0%). All solutions were treated by stirring with Chelex (Sigma-Aldrich, 50–100 mesh) for at least one hour prior to use. Electrolytes were purged with vigorous CO_2_ bubbling for at least 15 min to achieve a stable pH/outlet flow rate of CO_2_ from solution before use, then subsequently purged with the working gas.

### Electrode Preparation

For RDE measurements, 20 μL of the catalyst suspension (10 mg/mL, 0.3 Nafion/catalyst mass ratio in ethanol) was dropcast onto a glassy carbon disk electrode (Pine Research Instrumentation, 5 mm diameter) and allowed to dry naturally in air. For flow cell measurements, pieces of AvCarb MGL370 (Fuel Cell Store) were cut into 4 cm × 4 cm squares and 200 μL of catalyst dispersion (6 mg/mL, 0.3 Nafion/catalyst mass ratio in ethanol) was dropcast onto the squares and allowed to dry naturally. Nafion was added from a corresponding amount of 5 wt % solution (Sigma-Aldrich).

### Electrochemical Measurements

All electrochemical measurements were conducted on a VSP-300 poteniostat. RDE measurements were conducted in a sealed rotating cell, as described previously. Flow cell measurements were conducted in custom 3D printed flow cells (Formlabs, Clear v4 resin). The two-compartment cell was separated using an anion exchange membrane (Fumasep FAS-50, Fuel Cell Store). All measurements used a leakless Ag/AgCl electrode (Innovative Instruments Inc.) and typically used an iridium oxide on carbon paper anode (Dioxide Materials). The anolyte was either a 0.1 or 1 M NaHCO_3_ solution (to correspond to the same catholyte composition for CO_2_R) or 1 M NaClO_4_ (for RCC). The anolyte compartment was static and contained ∼9 mL of electrolyte. For most tests, a pH probe (H1001, Hanna Instruments) was placed in-line in the catholyte flow loop by screwing into a 3D printed holder.

Gaseous products were detected with an online gas chromatogram (SRI 8610C MG#5) equipped with a 12 ft MolSieve 5A column (for H_2_ separation), 6 ft HayeSep D column (for CO/CO_2_ separation), flame ionization detector (FID) for CO/CO_2_ quantification and a thermal conductivity detector (TCD) for H_2_ quantification. RDE measurements were conducted at 800 rpm unless otherwise noted and flow cell measurements were conducted at a liquid flow rate of 40 mL/min with 75 mL of recirculating electrolyte unless otherwise noted.

### X-ray Absorption Spectroscopy

X-ray absorption spectroscopy (XAS) measurements were conducted in fluorescence mode at beamline 4–1 of the Stanford Synchrotron Radiation Lightsource (SSRL). *In situ* XAS measurements were conducted in 3D printed flow cells similar to those used for performance benchmarking, except with a beveled opening on the front faceplate. The catalyst electrodes were sealed in on the front of the working chamber using Kapton tape. Data was processed using Larix/Larch or Athena. Fittings were processed in Artemis To obtain an estimate of the amplitude reduction factor, *S*_0_^2^, we fitted the measure spectra of Ni­(cyclam), a molecular complex assumed to have a first shell Ni–N coordination of 4. By fixing the coordination number at a value of 4, we obtained *S*_0_^2^ = 0.8 ± 0.2, so we carried forward a fixed value of *S*_0_^2^ = 0.85 for the fitting of the Ni–N–C EXAFS. For the Ni–N–C fitting we also fixed the Ni–N/C distance as 1.87 Å (from the optimized Ni–N_4_ structure), the Ni–C coordination number to be the four minus the Ni–N coordination number, and guessed all other parameters.

### ICP-MS

ICP-MS standards were prepared from nickel nitrate hexahydrate and prepared in a matrix of 0.5 M DEA/0.5 NaClO_4_. Aliquots from the electrolyte were diluted 100-fold in a matrix of 0.5 M DEA/0.5 M NaClO_4_.

## Supplementary Material



## Notes

Additional computational details, additional data related to the flow cell testing of the Ni–N–C catalyst, VLE modeling of DEA, photo of assembled cell for XAS, and quantification of Ni leaching from flow cell testing (PDF)
