# Supplementary material for: Solution and Active Site Speciation Drive Selectivity for Electrocatalytic Reactive Carbon Capture in Diethanolamine over Ni–N–C Catalysts
Source: J Am Chem Soc. 2026 Jan 22;148(4):3962–75. doi: 10.1021/jacs.5c11791 (PMC12879936; doi:10.1021/jacs.5c11791)
Supplement: Supplementary file 1 [file ja5c11791_si_001.pdf]

Supporting Information for:

Solution and Active Site Speciation Drive Selectivity for Electrocatalytic Reactive Carbon Capture in Diethanolamine over Ni-N-C Catalysts

R. Dominic Ross,[1,2,‡] Yulan Han,[3,‡] Hui-Yun Jeong,[1,2] Jenna M. Ynzunza,[4] Robert H. Lavroff,[3] Avishek Banerjee,[5] Aditya Prajapati,[1, 2] Carlos G. Morales-Guio,[5] Jesús M. Velázquez,[4] Anastassia N. Alexandrova,[3] Christopher Hahn[1,2*]*

[1]Materials Science Division, Lawrence Livermore National Laboratory, Livermore, CA 94550

[2]Laboratory for Energy Applications for the Future (LEAF), Lawrence Livermore National Laboratory, Livermore, CA 94550

[3]Department of Chemistry and Biochemistry, University of California, Los Angeles, Los Angeles, CA 90095

[4]Department of Chemistry, University of California, Davis, Davis, CA 95616

[5]Department of Chemical and Biomolecular Engineering, University of California, Los Angeles, Los Angeles, CA 90095

[‡]These authors contributed equally.

[*]Corresponding author emails: alexandrova@g.ucla.edu, hahn31@llnl.gov

Supporting Methods

Computational details

Grand canonical density functional theory (GCDFT) calculations

The chemical potential of a coupled proton and electron is determined by the equilibrium with H_2 gas at the hydrogen electrode, expressed as:

$$\mu_{\mathrm{H^+/e^-}} = \frac{1}{2}\mu_{\mathrm{H_2}} - eU - \ln(10)k_{\mathrm{B}}TpH \qquad (1)$$

Here, U is the applied potential vs SHE, and the last term accounts for pH correction at finite temperature.

The grand canonical free energy was evaluated by grand canonical density functional theory (GCDFT) calculations, which is a surface charging technique. Details can be found in our previous work[1–5], and here we summarize the key points.

The net charge of the electrons n_{surface} is obtained as:

$$n_{\mathrm{surface}} = N_{\mathrm{surface}} - N_{\mathrm{surface,neutral}} \qquad (2)$$

Where N_{surface} is the total number of electrons in the charged system and $N_{\mathrm{surface,neutral}}$ corresponds to the neutral reference.

The total DFT energy of the charged system is given by:

$$E_{\mathrm{surface}} = E_{\mathrm{surface,raw}} + \epsilon_{\mathrm{fermishift}} n_{\mathrm{surface}} \qquad (3)$$

Where $E_{\mathrm{surface,raw}}$ is the raw electronic energy of the surface and $\epsilon_{\mathrm{fermishift}} n_{\mathrm{surface}}$ is the term accounting for the difference($\epsilon_{\mathrm{fermishift}}$) is the reference energy of the electron between the

"internal" reference level and vacuum. Then, the grand canonical electronic energy of a surface model, $\Omega_{(\mathrm{U})}$, is obtained as:

$$\Omega_{(\mathrm{U})} = E_{\mathrm{surface}} - n_{\mathrm{surface}}\mu_{\mathrm{electron}} \qquad (4)$$

Where μ_{electron} is the chemical potential of an electron, which is defined as:

$$\mu_{\mathrm{electron}} = qU_{vac} = -eU_{\mathrm{vac}} \qquad (5)$$

Where U_{vac} is the potential of the system with reference to the vacuum level and q is the charge of an electron. The potential of the system with reference to the vacuum can be determined by two components, the Fermi level (ϵ_{F}) with reference to the "internal" zero energy references and Fermi shift which is the difference between the "internal" energy reference and the vacuum level:

$$-eU_{\mathrm{vac}} = \epsilon_{\mathrm{F}} + \epsilon_{\mathrm{fermi\ shift}} \qquad (6)$$

For the metallic systems, the potential-dependent grand canonical energy, $\Omega_{(\mathrm{U})}$, exhibits a quadratic behavior around the potential of zero charge (U_0) in the vacuum scale:

$$\Omega_{(\mathrm{U})} = \Omega_{(\mathrm{U_0})} = \frac{1}{2}C(U - U_0)^2 \qquad (7)$$

Where C is the capacitance of the surface. The potential of the system with respect to the standard hydrogen electrode (SHE) can be converted from U_{vac} as:

$$\mathrm{U_{SHE}} = U_{\mathrm{vac}} - 4.44 \qquad (8)$$

The linearized Poisson Boltzmann implicit solvation model implemented in VASPsol is used to represent the polarizable electrolyte region.[6] The dielectric constant of water, 78.4, and the Debye screening length corresponding to 1 M concentration of electrolytes, 3.0 Å, were used. The surface

slab is symmetrized along the z axis to avoid asymmetric potential in the implicit solvation region. Here the implicit solvent thickness is set to 60 Å for the symmetrized slab.

Quantum embedding approach

Hartree Fock (HF) calculations on the full unit cell were performed with a pob-TZVP-rev2 Gaussian basis[7] and 25 k-points (5 in each lattice vector direction of the slab), using the Crystal code.[8] Crystal was then also used to obtain Boys-localized Wannier functions (WFs) to serve as the basis for the occupied fragment orbitals. Formation of projected atomic orbitals (PAOs) and the embedded fragment density-fitted HF procedure (including projection to make the WFs and PAOs orthogonal in this partially delocalized system) was done in Cryscor[9], with a fitting basis optimized for MP2/cc-pVTZ calculations[10], resulting in FCIDUMP[11] interface files used to treat the active site with post-HF correlated calculations. Only valence WFs were obtained, meaning all post-HF calculations were performed with the frozen-core approximation.

Two embedded fragment sizes were tested, 19-atoms and 25-atoms, shown in **Figure S19,** with the remainder of the periodic, graphitic surroundings frozen in the Hartree-Fock mean field. Restricted MP2 and CCSD orbital-unrelaxed 1-particle density matrices were obtained using pySCF[12] and diagonalized to obtain natural orbital occupation numbers. The highest three “occupied” NOs and lowest three “virtual” NOs are highly fractionally occupied, leading to selection of a (6 electron,6 orbital) active space. CASSCF and CASPT2[13] calculations were subsequently and separately (i.e. state-specifically) performed in the Molpro[14] package for the lowest singlet, triplet, and quintet state. Examination of the CASSCF natural orbital occupations shows the active site ground-state to decisively be a closed-shell singlet, which is consistent with the ligand-field theory prediction of Ni^{2+} in a square planar environment ($3d_{xy}$ being the HOMO and $3d_{x2-y2}$ the LUMO), as well as our unrestricted, plane-wave DFT calculations.

Since closed-shell singlet states tend not to be multiconfigurational, and there is also some precedent for accurate CCSD(T) spin ordering from the porphyrin literature[15], we performed CCSD(T) calculations on this state and restricted-open-shell-CCSD(T) on the triplet and quintet states. These predicted a 1.30 eV singlet-triplet gap for the 19-atom fragment (versus 3.60 eV predicted by CASPT2(6,6) and 2.75 eV by CCSD), while the quintet state did not converge after hundreds of CCSD iterations but was stably hovering at ~13.2 eV above the singlet (8.2 eV for converged CASPT2) and thus deemed irrelevant. For the 25-atom fragment, CCSD(T) predicted a 2.26 eV singlet-triplet gap (versus 2.84 eV predicted by CASPT2(6,6) and 2.60 eV for CCSD). The T1 diagnostic for CCSD predicts that the ground-state may have some multiconfigurational character, with both fragment sizes giving values of ~0.037 for the singlet and ~0.128 for the triplet. An unofficial guideline is that less than 0.02 is considered decisively single-reference for closed-shell (or less than 0.03 for open-shell), and anything above 0.06 is not trustworthy to treat with a single-reference method.[16,17] For an excited-state study of this system, a multireference approach (including active space benchmarking) would certainly be required; however, we deem DFT to be trustworthy for the ground state given its agreement in terms of spin with CASPT2 and CCSD(T). There is some precedent for this from the porphyrin literature as well.[18]

Energy Span (ES) method

The span is defined by Kozuch et al.[19,20] In the Energy Span(ES) model, the reactivity of a given pathway can be approximated by the largest energy difference between a transition state on the pathway in question and an intermediate on this pathway in our work. Transitions states/free energy barriers are required to determine the ES and explicit determination of all potential dependent free energy barriers would be a huge endeavor. All elementary reaction steps are PCET

or proton transfer and hence present a strong similarity. For simplicity, we will assume that all the free energy barriers are equal, and a value of 0.4 eV has been assumed.[5]

XANES simulation

XANES spectra of the relevant structures were calculated using the finite difference method (FDM) and the Hedin–Lundqvist exchange-correlation potential as implemented in the FDMNES ab initio package.[21,22] FDMNES operates in real space and constructs a cluster of a specified radius around the absorbing atom; in our calculations, a cluster radius of 7.0 Å was used. The Ni K-edge (Z = 28) was computed with both dipole and quadrupole transitions included.

Supporting Tables

Table S1. EXAFS fitting parameters of Ni(cyclam) and Ni-N-C.

**Ni(cyclam)**	S_0^2	N	E_0	R (Å)	σ^2
Ni-N	0.8 ± 0.2	4	2 ± 2	2.08 ± 0.04	± 0.004
Ni-Cl		2		2.52 ± 0.04	

Table S2. EXAFS fitting parameters of Ni-N-C.

**Ni-N-C**	S_0^2	N	E_0	R (Å)	σ^2
Ni-N	0.85	3.3 ± 1	-7 ± 2	1.87	0.007 ± 0.002
Ni-C		0.7 ± 1			

Supporting Figures

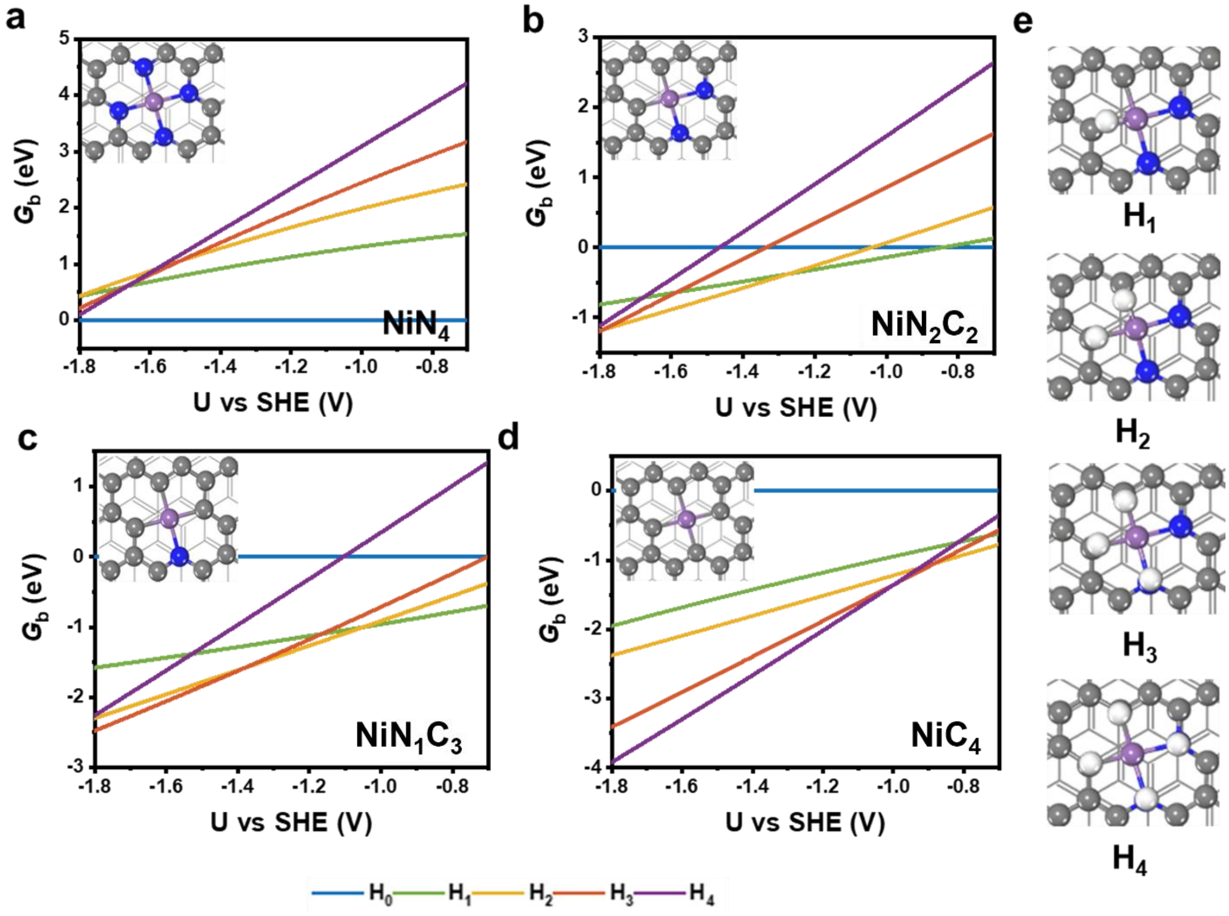

Figure S1. (a-d) Thermodynamic stability of NiN_xC_{4-x} catalyst surfaces under varying hydrogen coverages. Insets display optimized structures. (e) Representative structures illustrating different hydrogen coverages on NiC_2N_2 as an example. Color code of atoms: Ni (purple), N (blue), C (gray), and H (white).

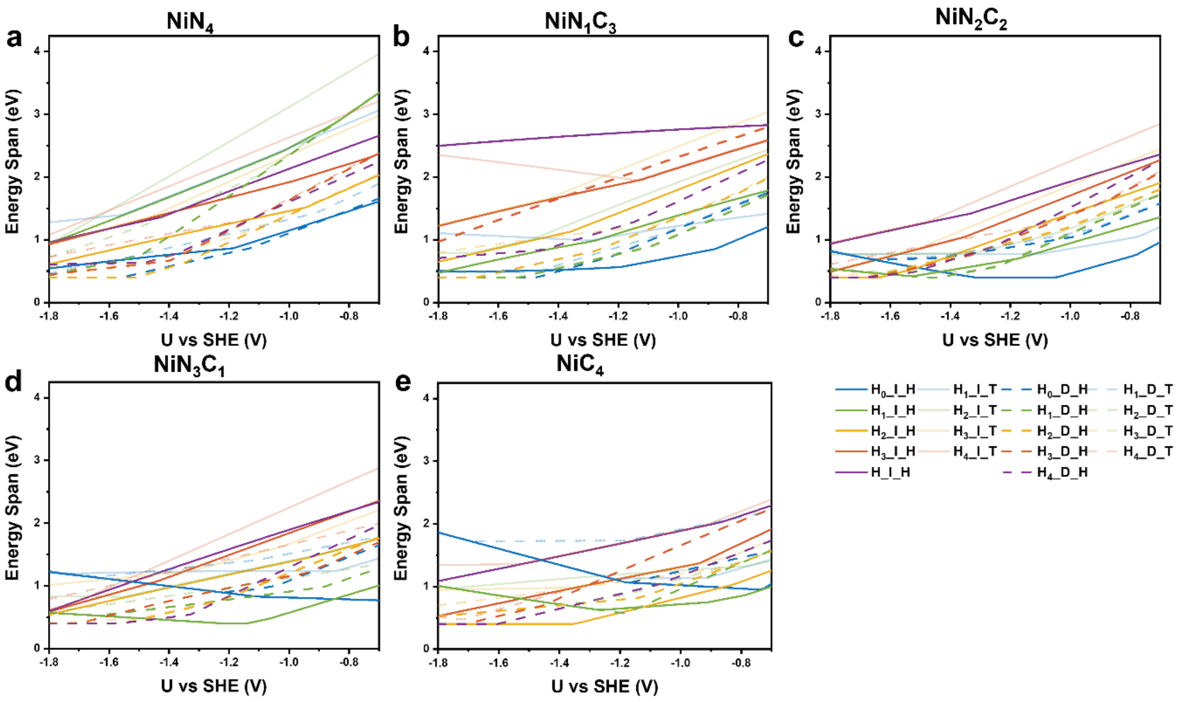

Figure S2. Calculated ES for the RCC on different NiC_xN_γ as a function of the applied potential. Hn_I_H represents the I_H reaction pathway of the catalyst with n hydrogen atoms.

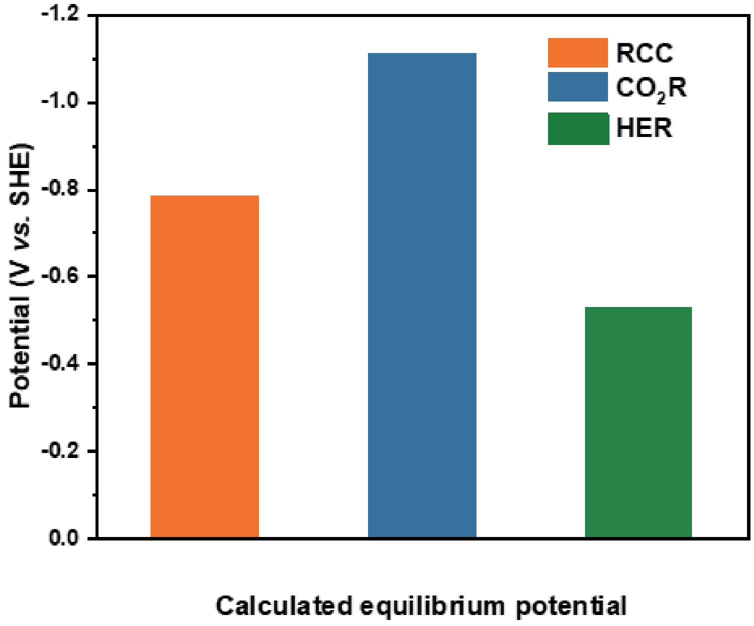

Figure S3. Calculated equilibrium potentials for RCC, CO_2R, and HER for $NaDEACO_2$ complex in DEA solvent.

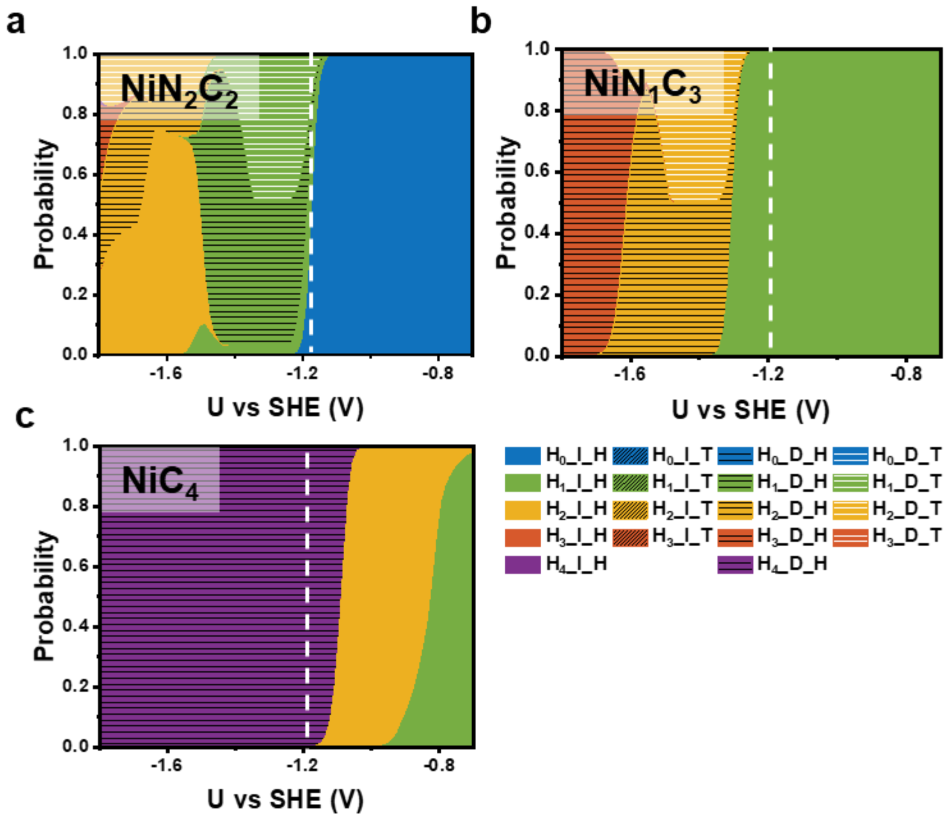

Figure S4. Probability distribution of different RCC reaction pathways for catalysts with varying hydrogen coverages, highlighting their contributions to the reaction rate.

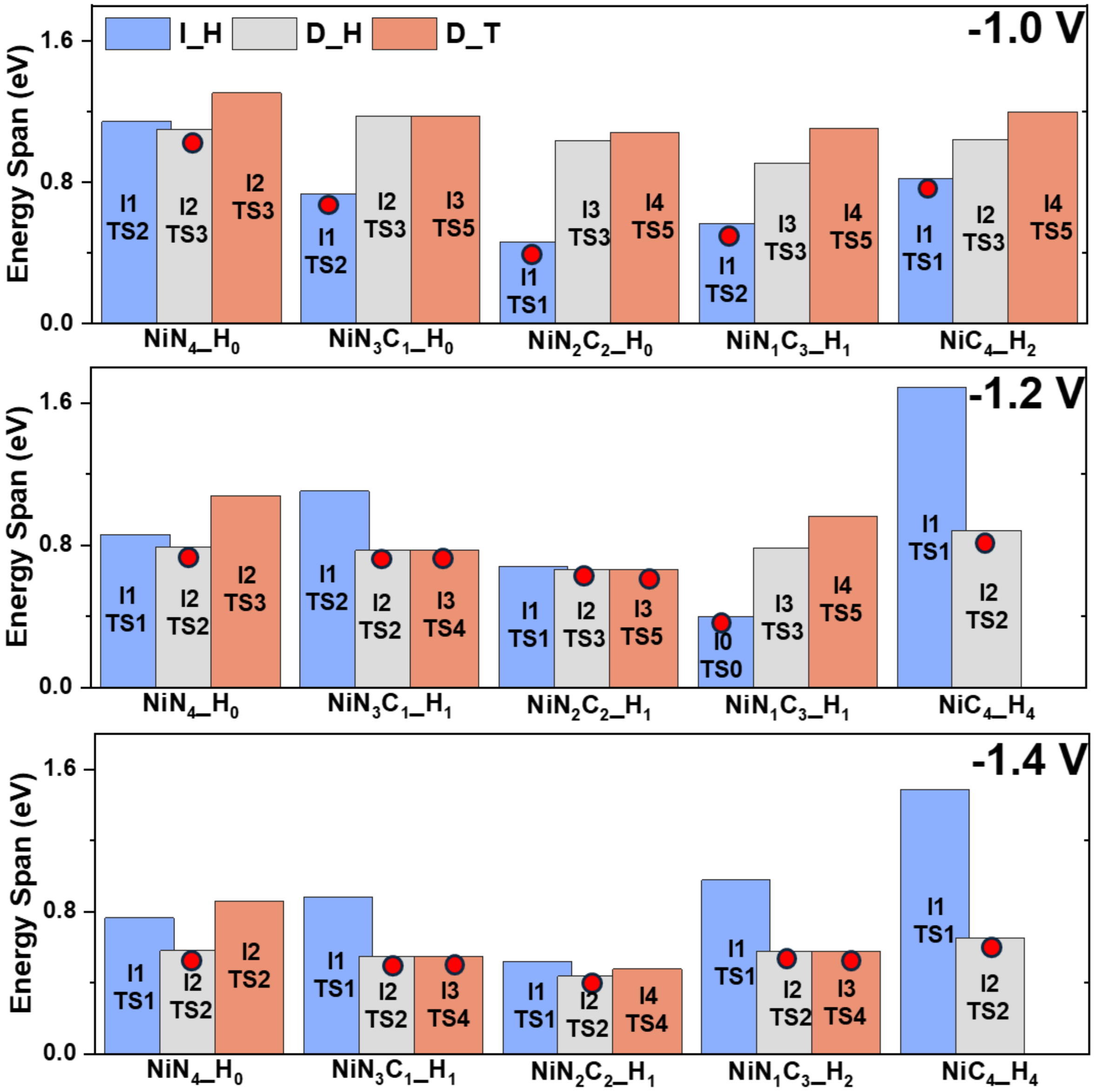

Figure S5. The rate-determining process of different reaction pathways for NiN_xC_{4-x} under varying applied voltages: -1.0 V, -1.2 V, and -1.4 V. The energy span of each reaction pathway is shown with different colored bars: I_H (blue), D_H (gray), and D_T (red). The red dots indicate the reaction progress with the highest energy contribution for each catalytic surface and voltage condition.

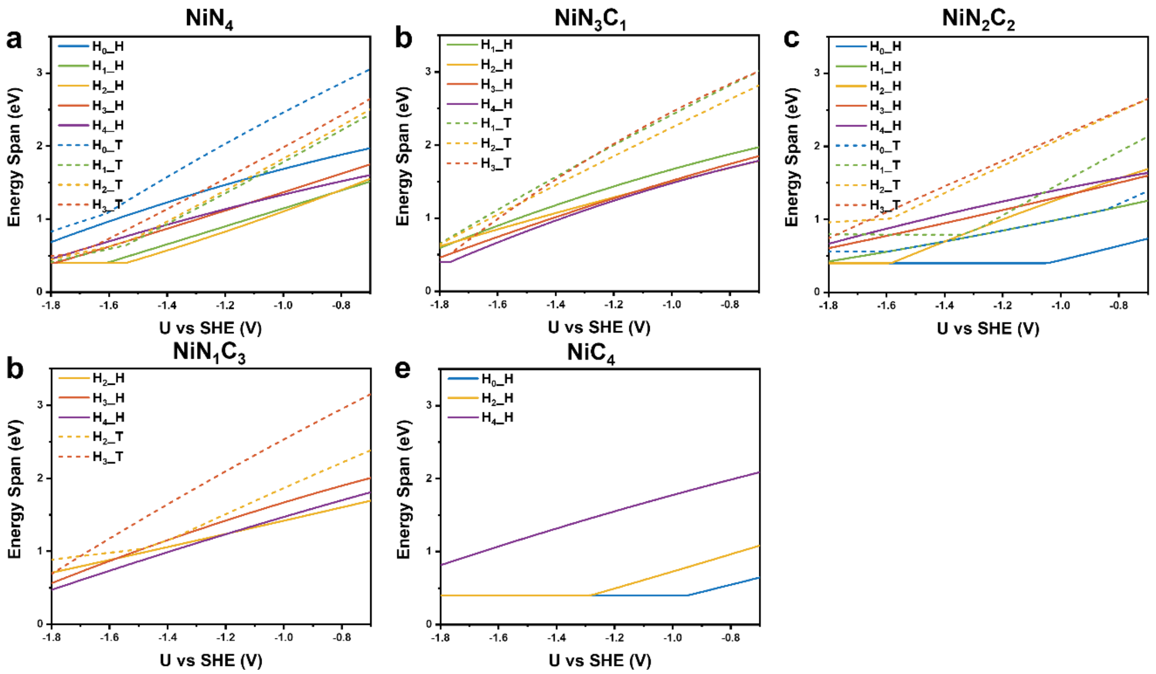

Figure S6. Calculated ES for the HER on different NiC_xN_y as a function of the applied potential.

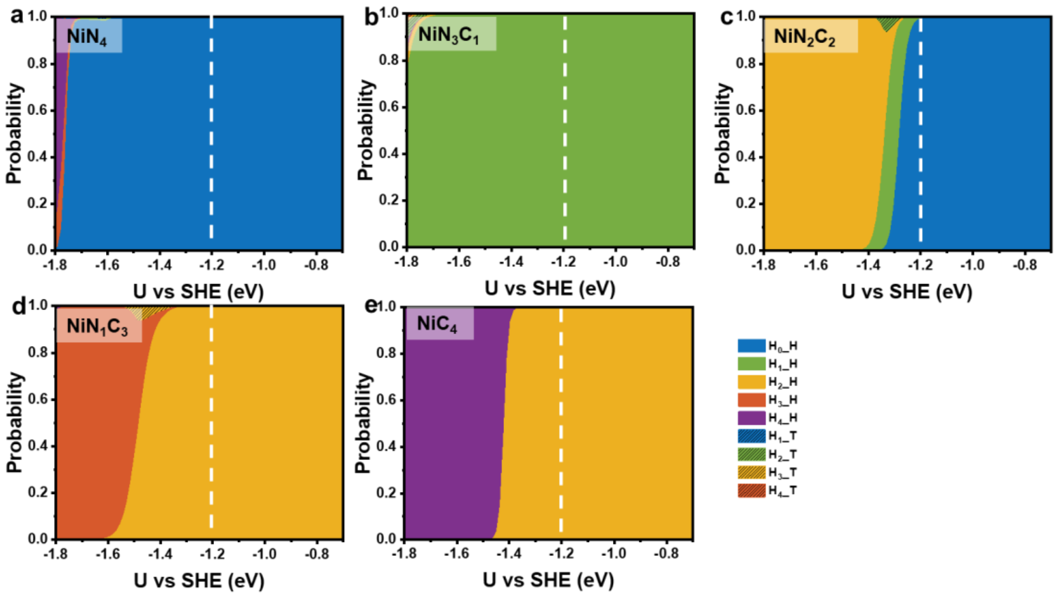

Figure S7. Probability distribution of different HER reaction pathways for catalysts with varying hydrogen coverages, highlighting their contributions to the reaction rate.

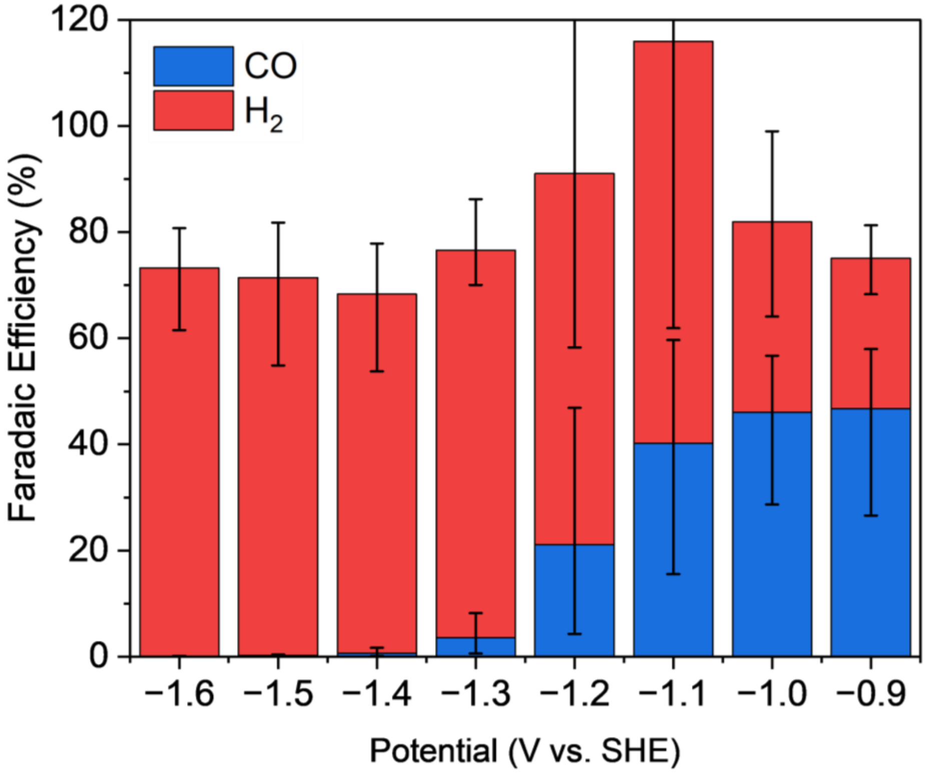

Figure S8. Faradaic efficiencies of Ni-N-C on a glassy carbon RDE measured at various potentials.

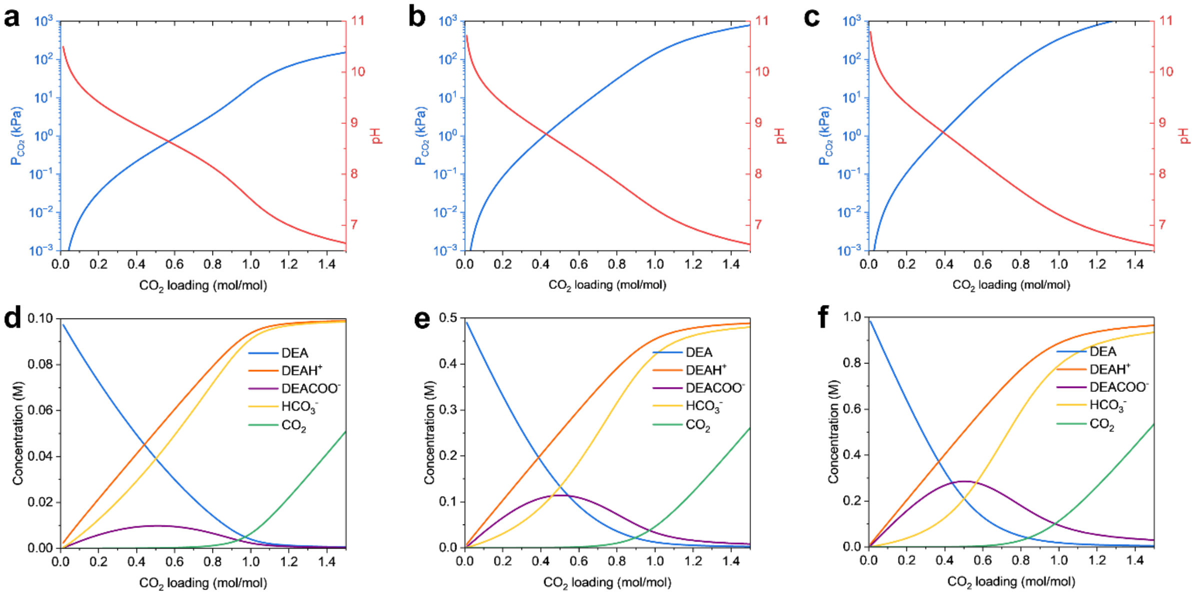

Figure S9. Vapor-liquid equilibrium models of a,d) 0.1 M DEA, b,e) 0.5 M DEA, and c,f) 1 M DEA. Details on the development of the VLE model for DEA are reported in Banerjee et al.[23]

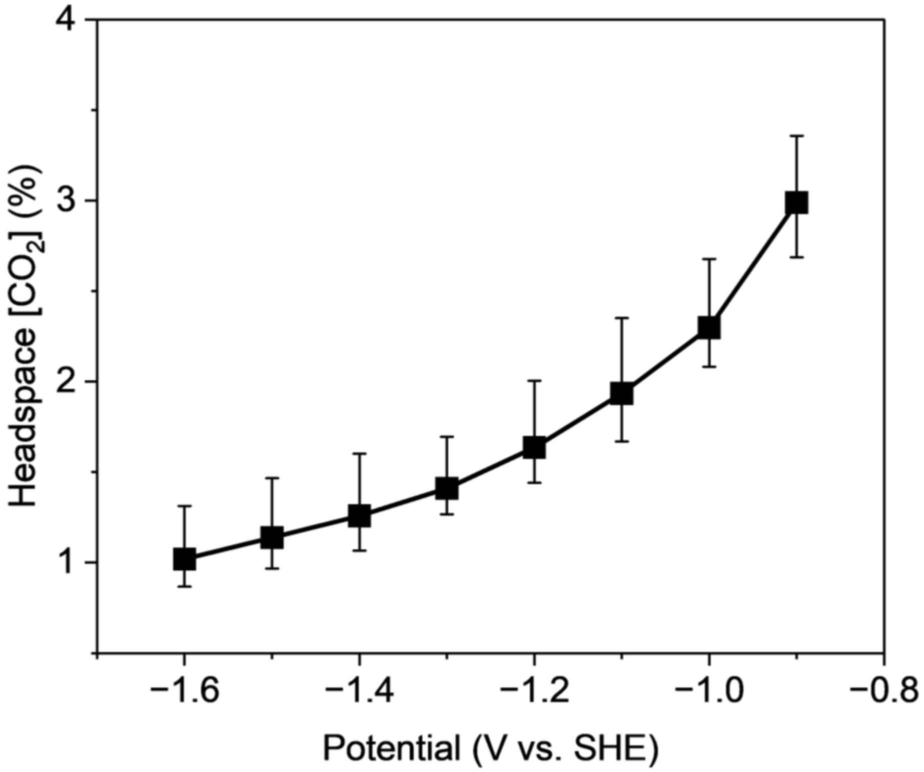

Figure S10. Headspace CO_2 concentration during potential dependent RDE tests, in which each potential was measured sequentially (for three separate parallel tests).

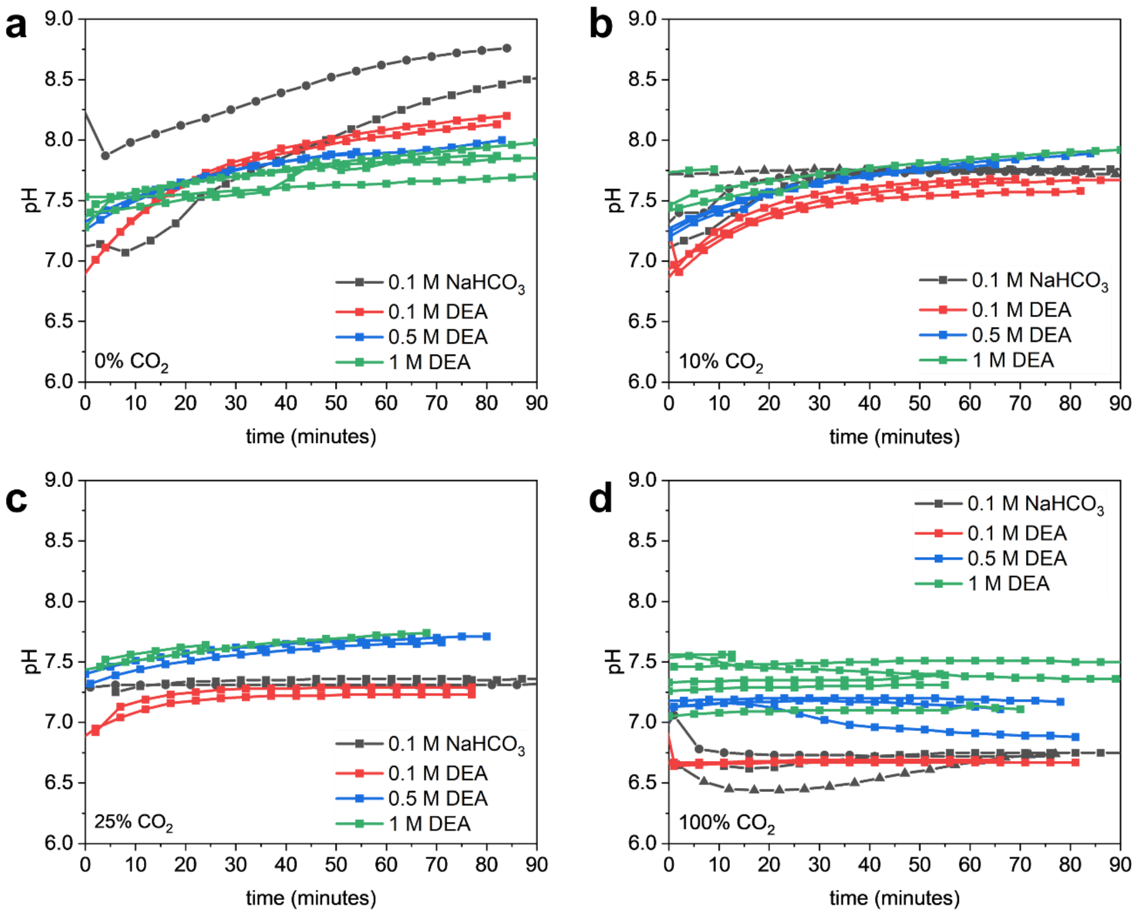

Figure S11. pH monitoring of tests (each individual line represents a separate test) from Figure 4 with a) 0% CO_2 (pure Ar), b) 10% CO_2 in Ar, c) 25% CO_2 in Ar, and d) 100% CO_2.

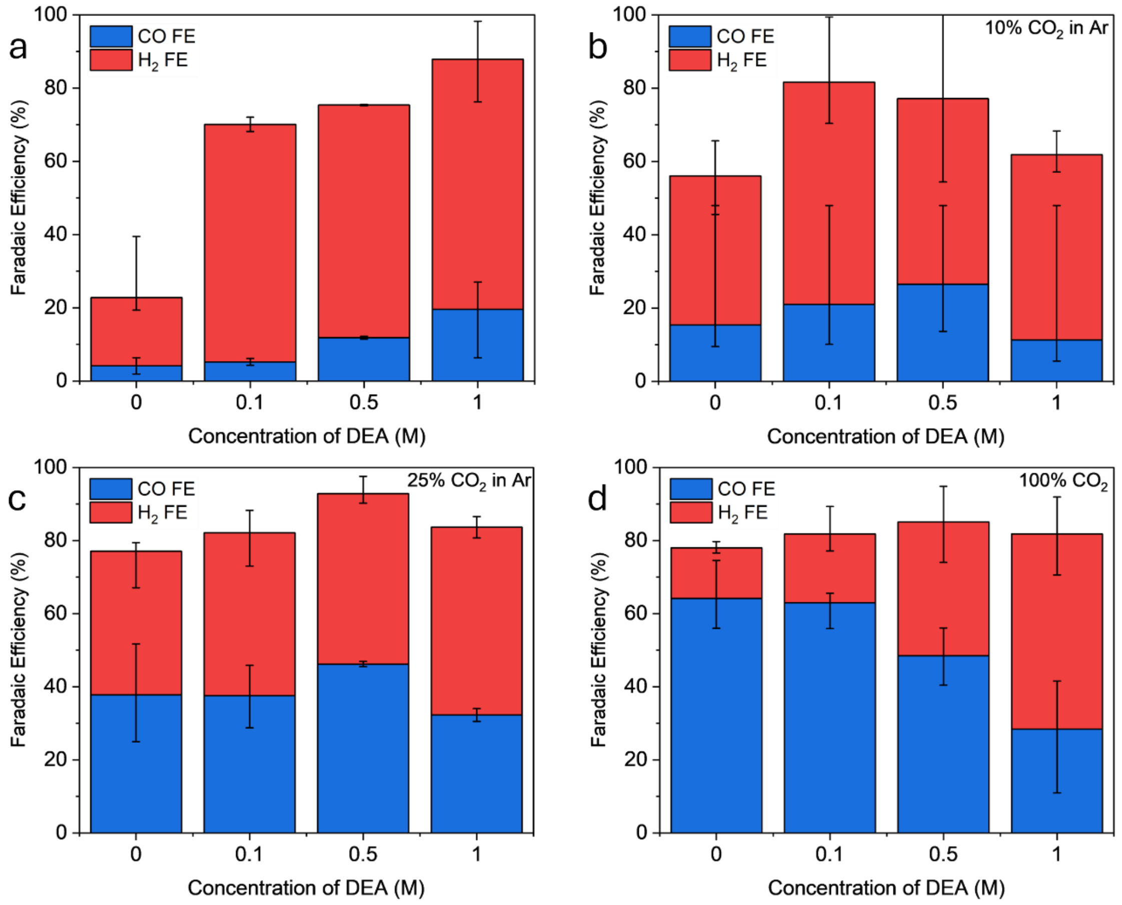

Figure S12. Faradaic efficiencies of Ni-N-C/carbon paper in a flow cell with a) pure Ar gas, b) 10% CO_2 in Ar, c) 25% CO_2 in Ar, and d) pure CO_2 gas.

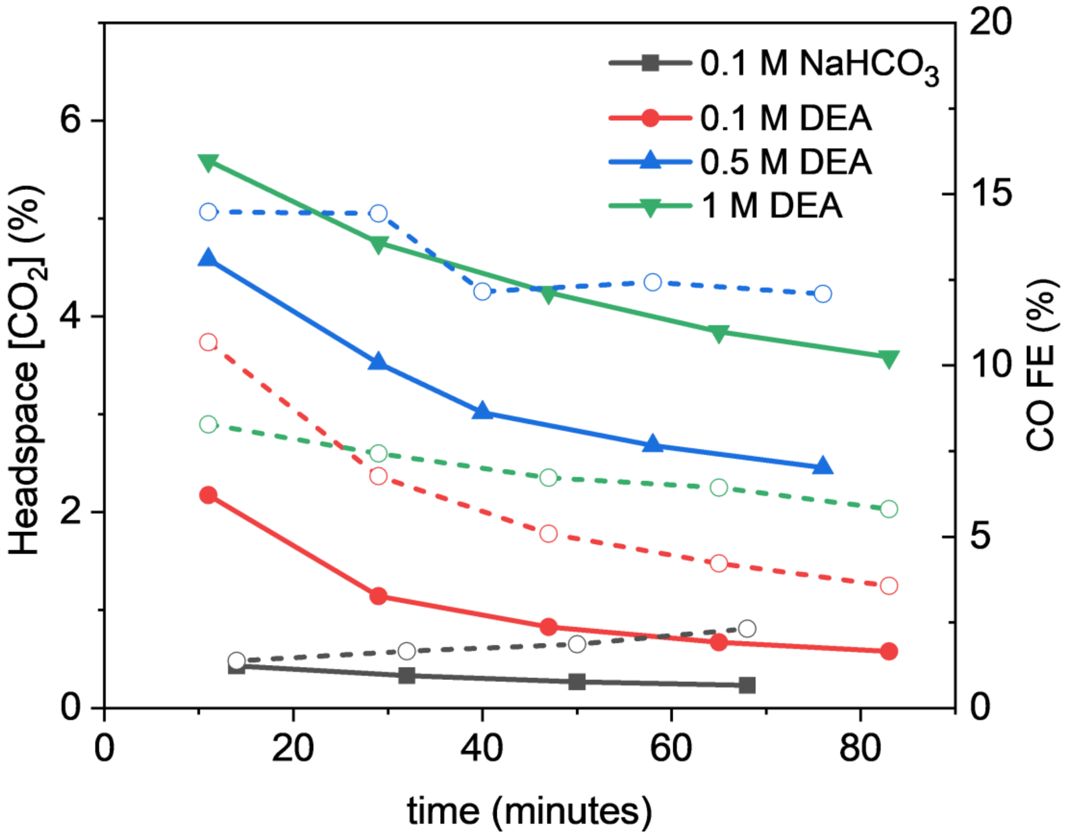

Figure S13. Representative changes in headspace CO_2 concentrations (closed shapes, left-axis) and corresponding drops in CO Faradaic efficiency (open circles, right-axis) for tests under Ar flow with different electrolytes in the flow cell.

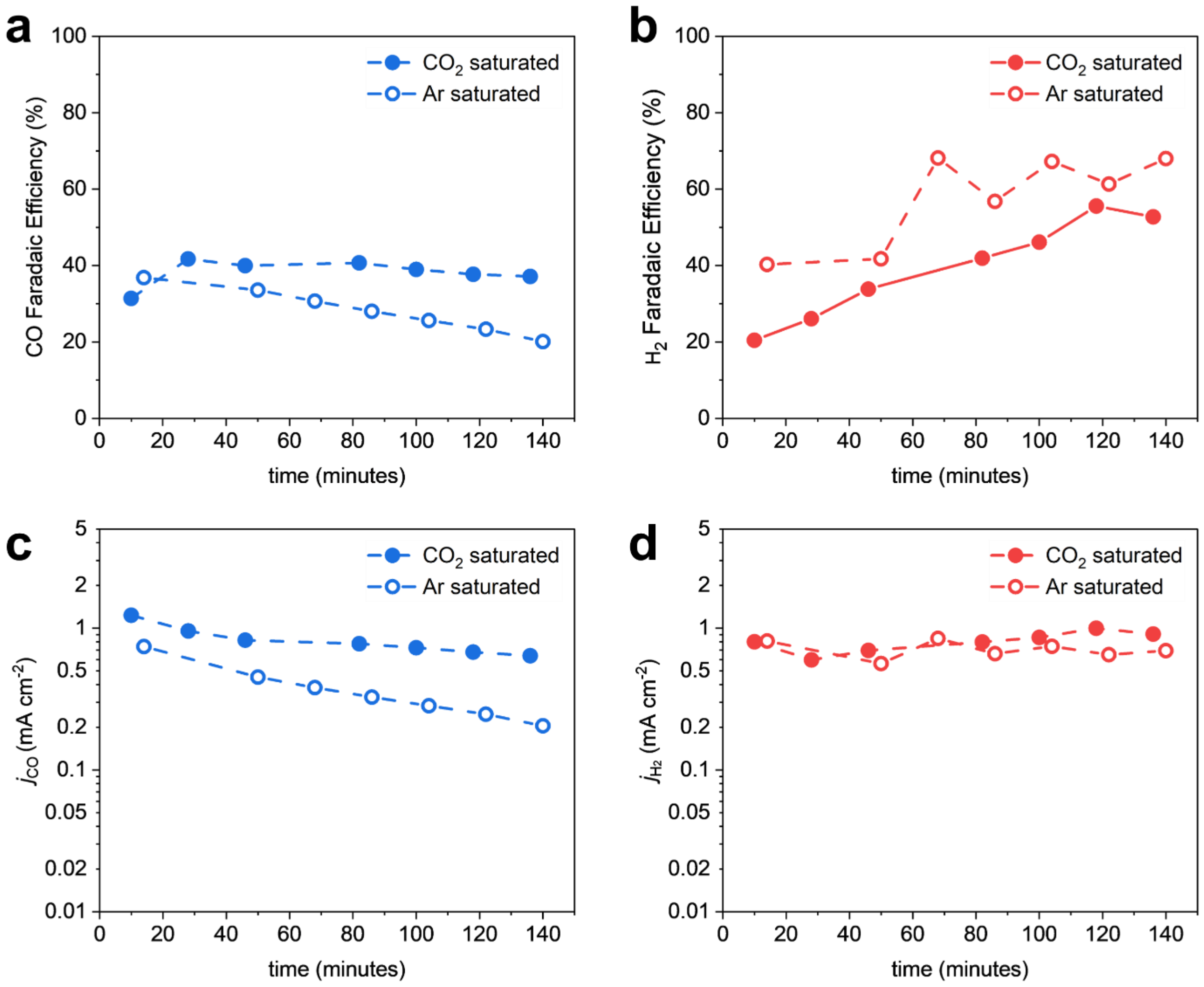

Figure S14. Representative example of time dependence of detected CO and H_2 over a >2 hour test (Ni-N-C/carbon paper, -1.1 V vs. SHE, 1 M DEA/1 M $NaClO_4$) in the flow cell under pure CO_2 or Ar flow, showing Faradaic efficiencies of a) CO, and b) H_2, and corresponding partial current densities of c) CO and d) H_2. In this test, the pH shifted from ~7.3 to ~7.8 and the CO_2 concentration in the headspace shifted from ~5% to ~3% under Ar flow.

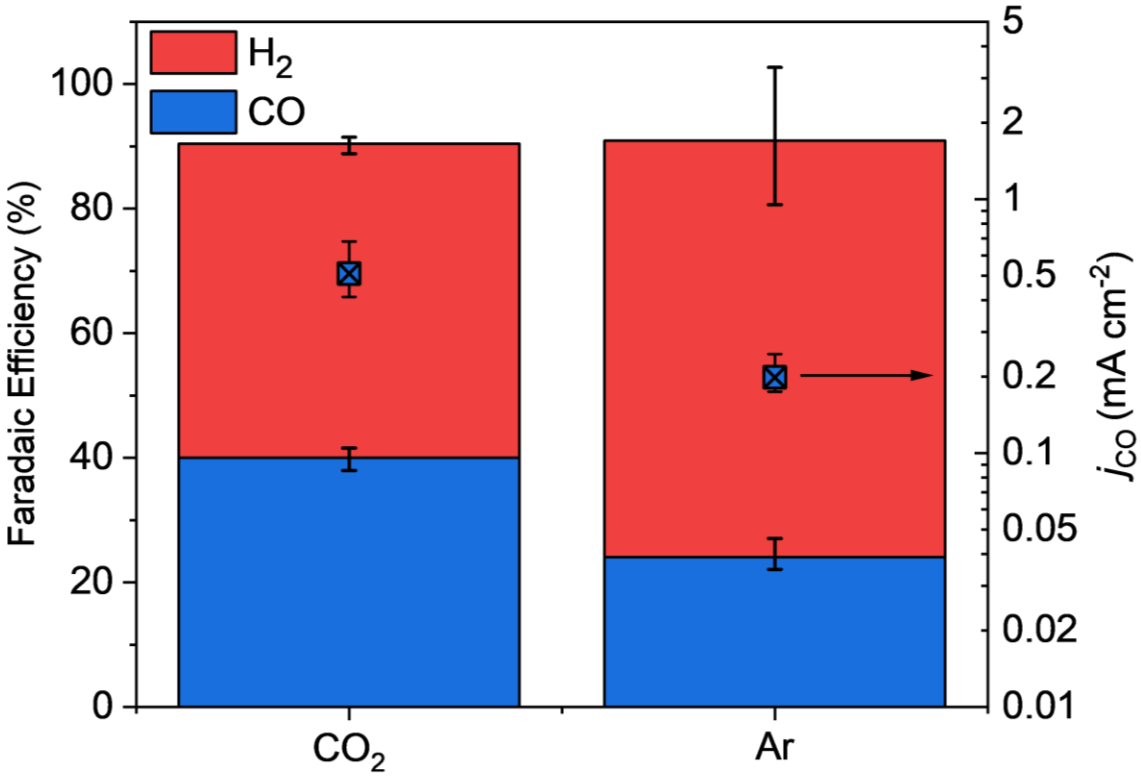

Figure S15. Average Faradaic efficiencies (bars, left-axis) and average partial current densities (squares, right-axis) of three additional parallel tests with Ni-N-C/carbon paper in a flow cell with pure CO_2 and pure Ar gas in 1 M DEA/1 M $NaClO_4$ electrolyte. All tests were held at a constant potential of -1.1 V vs. SHE and the Ar saturated tests were held for at least 2 hours. The same electrode was first measured under the pure CO_2 before switching to pure Ar.

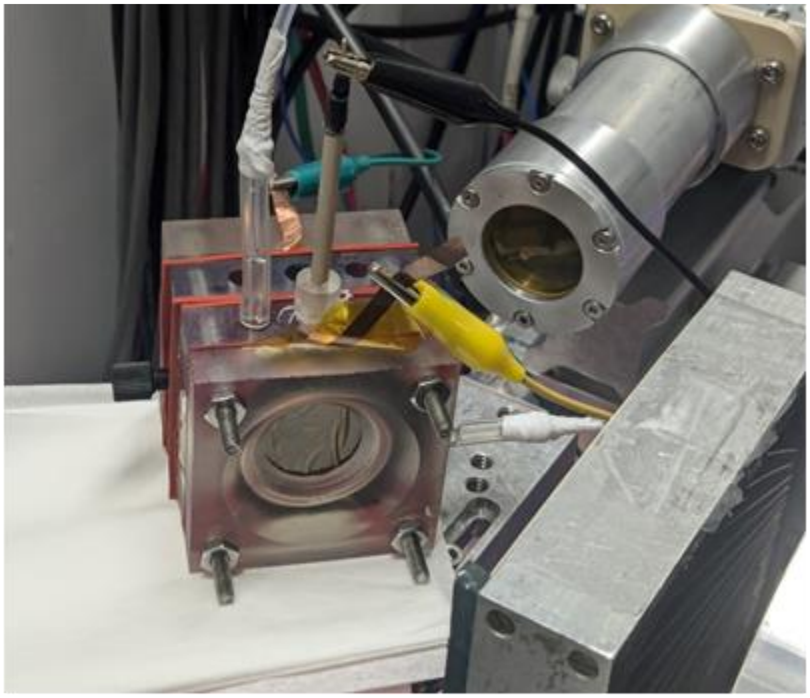

Figure S16. Photo of assembled *in situ* XAS cell at SSRL.

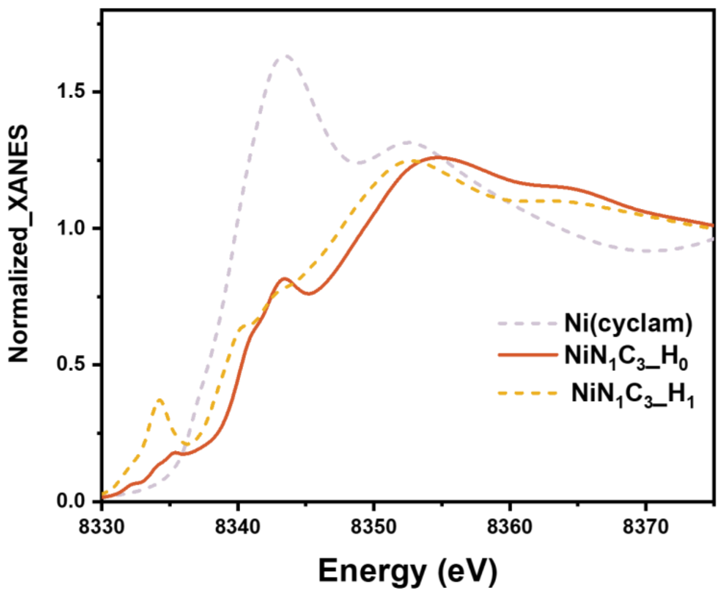

Figure S17. Simulated XANES spectra of NiN_1C_3 with ($NiN_1C_3\_H_1$) and without ($NiN_1C_3\_H_0$) absorbed hydrogen on the coordinating carbon, with the simulated spectrum of Ni(cyclam) shown for comparison.

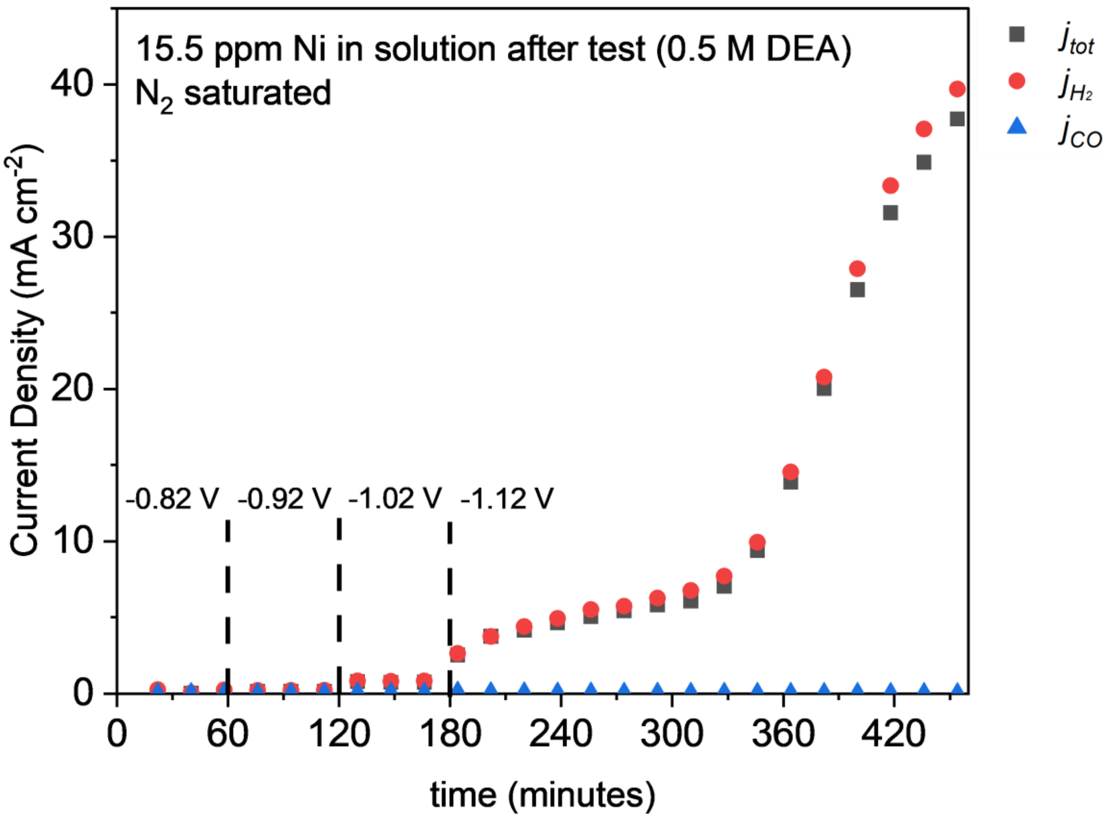

Figure S18. Representative long-term test of Ni-N-C catalyst in 0.5 M DEA/0.5 $NaClO_4$ under inert-gas saturation with resulting Ni leaching measured by ICP-MS (15.5 ppm).

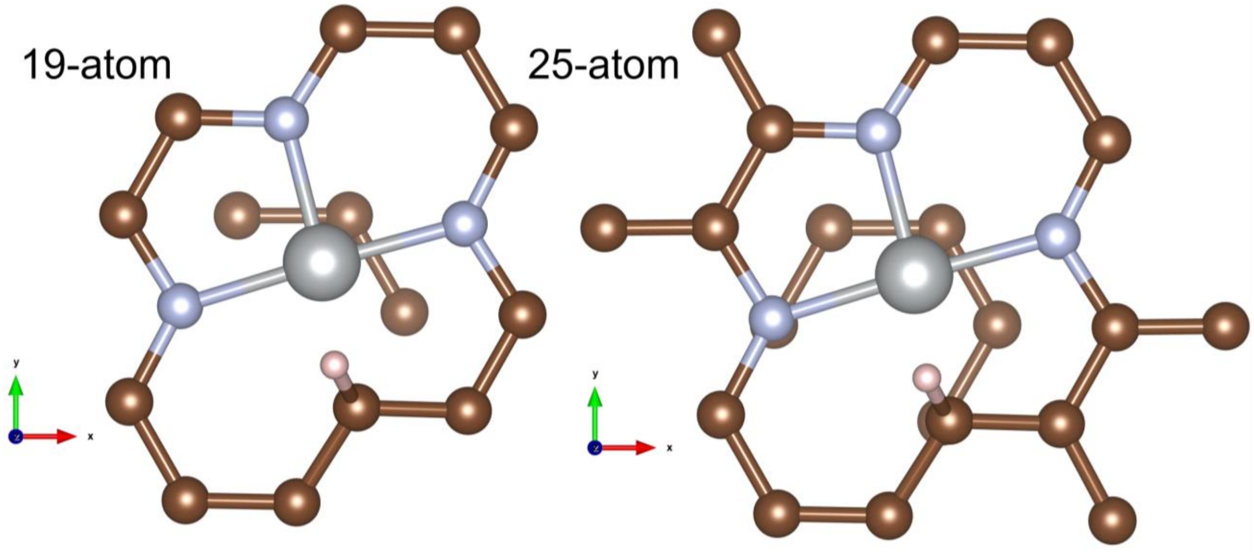

Figure S19. The two fragment sizes used for quantum embedding calculations on spin gaps to test the validity of DFT. All electrons for the 19-atom (left) and 25-atom (right) fragments were treated at the correlated post-HF levels of CASPT2(6,6) and CCSD(T), while their periodic surroundings were frozen at the mean-field level (HF). Carbon atoms are shown in brown, nitrogen in light blue, nickel in silver, and hydrogen in light pink.

Optimized POSCAR for NiN_3C_1

CNNi

1.0

12.3386201859	0.0000000000	0.0000000000
-6.1693115158	10.6855577071	0.0000000000
0.0000000000	0.0000000000	26.0551109314

C N Ni

145 3 1

Direct

0.000000000	0.000000000	0.083329998
0.533330023	0.066670001	0.083329998
0.733330011	0.066670001	0.083329998
0.933329999	0.066670001	0.083329998
0.333330005	0.066670001	0.083329998
0.133330002	0.066670001	0.083329998
0.600000024	0.200000003	0.083329998
0.800000012	0.200000003	0.083329998
0.200000003	0.200000003	0.083329998
0.400000006	0.200000003	0.083329998
0.000000000	0.200000003	0.083329998
0.133330002	0.266669989	0.083329998
0.733330011	0.266669989	0.083329998
0.933329999	0.266669989	0.083329998
0.333330005	0.266669989	0.083329998
0.533330023	0.266669989	0.083329998
0.000000000	0.400000006	0.083329998
0.600000024	0.400000006	0.083329998
0.800000012	0.400000006	0.083329998
0.200000003	0.400000006	0.083329998

0.400000006	0.400000006	0.083329998
0.133330002	0.466670007	0.083329998
0.733330011	0.466670007	0.083329998
0.933329999	0.466670007	0.083329998
0.333330005	0.466670007	0.083329998
0.533330023	0.466670007	0.083329998
0.000000000	0.600000024	0.083329998
0.400000006	0.600000024	0.083329998
0.800000012	0.600000024	0.083329998
0.600000024	0.600000024	0.083329998
0.200000003	0.600000024	0.083329998
0.133330002	0.666670024	0.083329998
0.933329999	0.666670024	0.083329998
0.533330023	0.666670024	0.083329998
0.733330011	0.666670024	0.083329998
0.333330005	0.666670024	0.083329998
0.400000006	0.800000012	0.083329998
0.600000024	0.800000012	0.083329998
0.000000000	0.800000012	0.083329998
0.800000012	0.800000012	0.083329998
0.200000003	0.800000012	0.083329998
0.133330002	0.866670012	0.083329998
0.933329999	0.866670012	0.083329998
0.533330023	0.866670012	0.083329998
0.733330011	0.866670012	0.083329998
0.333330005	0.866670012	0.083329998
0.200000003	0.000000000	0.083329998
0.400000006	0.000000000	0.083329998
0.600000024	0.000000000	0.083329998
0.800000012	0.000000000	0.083329998

0.000000000	0.000000000	0.250000000
0.200000003	0.000000000	0.250000000
0.066670001	0.133330002	0.250000000
0.266669989	0.133330002	0.250000000
0.466670007	0.133330002	0.250000000
0.666670024	0.133330002	0.250000000
0.866670012	0.133330002	0.250000000
0.600000024	0.200000003	0.250000000
0.800000012	0.200000003	0.250000000
0.200000003	0.200000003	0.250000000
0.400000006	0.200000003	0.250000000
0.000000000	0.200000003	0.250000000
0.666670024	0.333330005	0.250000000
0.866670012	0.333330005	0.250000000
0.266669989	0.333330005	0.250000000
0.466670007	0.333330005	0.250000000
0.066670001	0.333330005	0.250000000
0.000000000	0.400000006	0.250000000
0.600000024	0.400000006	0.250000000
0.800000012	0.400000006	0.250000000
0.200000003	0.400000006	0.250000000
0.400000006	0.400000006	0.250000000
0.066670001	0.533330023	0.250000000
0.666670024	0.533330023	0.250000000
0.866670012	0.533330023	0.250000000
0.266669989	0.533330023	0.250000000
0.466670007	0.533330023	0.250000000
0.400000006	0.600000024	0.250000000
0.000000000	0.600000024	0.250000000
0.600000024	0.600000024	0.250000000

0.800000012	0.600000024	0.250000000
0.200000003	0.600000024	0.250000000
0.066670001	0.733330011	0.250000000
0.866670012	0.733330011	0.250000000
0.466670007	0.733330011	0.250000000
0.666670024	0.733330011	0.250000000
0.266669989	0.733330011	0.250000000
0.400000006	0.800000012	0.250000000
0.600000024	0.800000012	0.250000000
0.000000000	0.800000012	0.250000000
0.800000012	0.800000012	0.250000000
0.200000003	0.800000012	0.250000000
0.266669989	0.933329999	0.250000000
0.066670001	0.933329999	0.250000000
0.466670007	0.933329999	0.250000000
0.666670024	0.933329999	0.250000000
0.866670012	0.933329999	0.250000000
0.400000006	0.000000000	0.250000000
0.600000024	0.000000000	0.250000000
0.800000012	0.000000000	0.250000000
0.732029974	0.852779984	0.385329992
0.205840006	0.999949992	0.384640008
0.404949993	0.000260000	0.384420007
0.735819995	0.058820002	0.385690004
0.934010029	0.058580000	0.384849995
0.137679994	0.064570002	0.384440005
0.537859976	0.064549997	0.385039985
0.339859992	0.069810003	0.384440005
0.605229974	0.198880002	0.385529995
0.204559997	0.199120000	0.383529991

0.407160014	0.204339996	0.384330004
0.339560002	0.271739990	0.383659989
0.542020023	0.270960003	0.385100007
0.612519979	0.404060006	0.385320008
0.801190019	0.786660016	0.385490000
0.596650004	0.787249982	0.384820014
0.936399996	0.860260010	0.385529995
0.534950018	0.860520005	0.384559989
0.799759984	0.988319993	0.385439992
0.602859974	0.994660020	0.385280013
0.002620000	0.994740009	0.384889990
0.001100000	0.192939997	0.383899987
0.802860022	0.193090007	0.385459989
0.935790002	0.260349989	0.384119987
0.134519994	0.261869997	0.382889986
0.738229990	0.262010008	0.385829985
0.743579984	0.464260012	0.385890007
0.332729995	0.462280005	0.382200003
0.811429977	0.598919988	0.385850012
0.399710000	0.597710013	0.382569999
0.006950000	0.798129976	0.385560006
0.402200013	0.797439992	0.383340001
0.139630005	0.866209984	0.384869993
0.337720007	0.866190016	0.383700013
0.003460000	0.394389987	0.383219987
0.807160020	0.395779997	0.385780007
0.200949997	0.395579994	0.381940007
0.135590002	0.462740004	0.381929994
0.939639986	0.462700009	0.384550005
0.006380000	0.596369982	0.384519994

0.202010006	0.596130013	0.381980002
0.942229986	0.665030003	0.385710001
0.334320009	0.663709998	0.382259995
0.138150007	0.664279997	0.383249998
0.204740003	0.798590004	0.383890003
0.400840014	0.401930004	0.383280009
0.527279973	0.658429980	0.384070009
0.742020011	0.657140017	0.385600001
0.569509983	0.527899981	0.384330004

Optimized POSCAR of $NaRCO_2$ adsorbed on NiN_3C_1

HCNONaNi

1.0

12.3386201859	0.0000000000	0.0000000000
-6.1693115158	10.6855577071	0.0000000000
0.0000000000	0.0000000000	26.0551109314

H	C	N	O	Na	Ni
10	150	4	4	1	1

Direct

0.608449996	0.372880012	0.537760019
0.800379992	0.724520028	0.548399985
0.429949999	0.369029999	0.583790004
0.645959973	0.736109972	0.609179974
0.869960010	0.884230018	0.609239995
0.703270018	0.430559993	0.593879998
0.827759981	0.637000024	0.596010029
0.522899985	0.426660001	0.640810013
0.659720004	0.628189981	0.650520027
0.563210011	0.245220006	0.633270025
0.000000000	0.000000000	0.083329998

0.133330002	0.066670001	0.083329998
0.533330023	0.066670001	0.083329998
0.733330011	0.066670001	0.083329998
0.933329999	0.066670001	0.083329998
0.333330005	0.066670001	0.083329998
0.800000012	0.200000003	0.083329998
0.200000003	0.200000003	0.083329998
0.400000006	0.200000003	0.083329998
0.000000000	0.200000003	0.083329998
0.600000024	0.200000003	0.083329998
0.133330002	0.266669989	0.083329998
0.733330011	0.266669989	0.083329998
0.933329999	0.266669989	0.083329998
0.333330005	0.266669989	0.083329998
0.533330023	0.266669989	0.083329998
0.000000000	0.400000006	0.083329998
0.600000024	0.400000006	0.083329998
0.800000012	0.400000006	0.083329998
0.200000003	0.400000006	0.083329998
0.400000006	0.400000006	0.083329998
0.133330002	0.466670007	0.083329998
0.733330011	0.466670007	0.083329998
0.933329999	0.466670007	0.083329998
0.333330005	0.466670007	0.083329998
0.533330023	0.466670007	0.083329998
0.000000000	0.600000024	0.083329998
0.400000006	0.600000024	0.083329998
0.800000012	0.600000024	0.083329998
0.600000024	0.600000024	0.083329998
0.200000003	0.600000024	0.083329998

0.133330002	0.666670024	0.083329998
0.933329999	0.666670024	0.083329998
0.533330023	0.666670024	0.083329998
0.733330011	0.666670024	0.083329998
0.333330005	0.666670024	0.083329998
0.400000006	0.800000012	0.083329998
0.600000024	0.800000012	0.083329998
0.000000000	0.800000012	0.083329998
0.800000012	0.800000012	0.083329998
0.200000003	0.800000012	0.083329998
0.133330002	0.866670012	0.083329998
0.933329999	0.866670012	0.083329998
0.533330023	0.866670012	0.083329998
0.733330011	0.866670012	0.083329998
0.333330005	0.866670012	0.083329998
0.200000003	0.000000000	0.083329998
0.400000006	0.000000000	0.083329998
0.600000024	0.000000000	0.083329998
0.800000012	0.000000000	0.083329998
0.000000000	0.000000000	0.250000000
0.066670001	0.133330002	0.250000000
0.266669989	0.133330002	0.250000000
0.466670007	0.133330002	0.250000000
0.666670024	0.133330002	0.250000000
0.866670012	0.133330002	0.250000000
0.600000024	0.200000003	0.250000000
0.800000012	0.200000003	0.250000000
0.200000003	0.200000003	0.250000000
0.400000006	0.200000003	0.250000000
0.000000000	0.200000003	0.250000000

0.666670024	0.333330005	0.250000000
0.866670012	0.333330005	0.250000000
0.266669989	0.333330005	0.250000000
0.466670007	0.333330005	0.250000000
0.066670001	0.333330005	0.250000000
0.000000000	0.400000006	0.250000000
0.600000024	0.400000006	0.250000000
0.800000012	0.400000006	0.250000000
0.200000003	0.400000006	0.250000000
0.400000006	0.400000006	0.250000000
0.066670001	0.533330023	0.250000000
0.666670024	0.533330023	0.250000000
0.866670012	0.533330023	0.250000000
0.266669989	0.533330023	0.250000000
0.466670007	0.533330023	0.250000000
0.400000006	0.600000024	0.250000000
0.000000000	0.600000024	0.250000000
0.600000024	0.600000024	0.250000000
0.800000012	0.600000024	0.250000000
0.200000003	0.600000024	0.250000000
0.066670001	0.733330011	0.250000000
0.866670012	0.733330011	0.250000000
0.466670007	0.733330011	0.250000000
0.666670024	0.733330011	0.250000000
0.266669989	0.733330011	0.250000000
0.400000006	0.800000012	0.250000000
0.600000024	0.800000012	0.250000000
0.000000000	0.800000012	0.250000000
0.800000012	0.800000012	0.250000000
0.200000003	0.800000012	0.250000000

0.266669989	0.933329999	0.250000000
0.066670001	0.933329999	0.250000000
0.466670007	0.933329999	0.250000000
0.666670024	0.933329999	0.250000000
0.866670012	0.933329999	0.250000000
0.600000024	0.000000000	0.250000000
0.200000003	0.000000000	0.250000000
0.800000012	0.000000000	0.250000000
0.400000006	0.000000000	0.250000000
0.329950005	0.670620024	0.381320000
0.999090016	0.401470006	0.381839991
0.802670002	0.402590007	0.383089989
0.131270006	0.469639987	0.381289989
0.935140014	0.469650000	0.382239997
0.395200014	0.604449987	0.381199986
0.197630003	0.603079975	0.381179988
0.001840000	0.603200018	0.381810009
0.133690000	0.671159983	0.381309986
0.397890002	0.804270029	0.381790012
0.798510015	0.199890003	0.382860005
0.931460023	0.267329991	0.382360011
0.733919978	0.268900007	0.383150011
0.196360007	0.402170002	0.381440014
0.739030004	0.470840007	0.383469999
0.200450003	0.805390000	0.381419986
0.530610025	0.867190003	0.382319987
0.333429992	0.873000026	0.381859988
0.598389983	0.001400000	0.382889986
0.400530010	0.007030000	0.382470012
0.731329978	0.065569997	0.382979989

0.533500016	0.071390003	0.382959992
0.600870013	0.205880001	0.383219987
0.328099996	0.468840003	0.381320000
0.937590003	0.671729982	0.382279992
0.201389998	0.006840000	0.382180005
0.335390002	0.076640002	0.382470012
0.996609986	0.199699998	0.382400006
0.129960001	0.268500000	0.381999999
0.537400007	0.277830005	0.383150011
0.607940018	0.410840005	0.383329988
0.806879997	0.605449975	0.383069992
0.591989994	0.793720007	0.382099986
0.002310000	0.804840028	0.382099986
0.135220006	0.873059988	0.381770015
0.795210004	0.994960010	0.382900000
0.998049974	0.001270000	0.382569999
0.929600000	0.065300003	0.382750005
0.133100003	0.071220003	0.382330000
0.199959993	0.205789998	0.382120013
0.402469993	0.211109996	0.382600009
0.727320015	0.859239995	0.382719994
0.931590021	0.866770029	0.382649988
0.334870011	0.278499991	0.382050008
0.796400011	0.793079972	0.382889986
0.499300003	0.579140007	0.542569995
0.606029987	0.373389989	0.579990029
0.525510013	0.428570002	0.598280013
0.798330009	0.706889987	0.590049982
0.664919972	0.657989979	0.610140026
0.522669971	0.665130019	0.381370008

0.396160007 0.408699989 0.381630003
0.737429976 0.663619995 0.383139998
0.568809986 0.555180013 0.580380023
0.406480014 0.484499991 0.522180021
0.537150025 0.693889976 0.532750010
0.555100024 0.245490000 0.596029997
0.888360023 0.817610025 0.616840005
0.099720001 0.773850024 0.520039976
0.564809978 0.534650028 0.382209986

Optimized POSCAR of NaRCOOH adsorbed on NiN_3C_1

NaRCOOH
1.0
12.3386001587 0.0000000000 0.0000000000
-6.1693000793 10.6855411846 0.0000000000
0.0000000000 0.0000000000 26.0550994873
H C N O Na Ni
11 150 4 4 1 1
Direct
0.616800010 0.509819984 0.460189998
0.843890011 0.742500007 0.549300015
0.535700023 0.746510029 0.552070022
0.730790019 0.520129979 0.579460025
0.353390008 0.692049980 0.594860017
0.414000005 0.529590011 0.594860017
0.804989994 0.790989995 0.607630014
0.615040004 0.803759992 0.611790001
0.691619992 0.566929996 0.637769997
0.526719987 0.589829981 0.645250022
0.925360024 0.722660005 0.649349988
0.000000000 0.000000000 0.083329998

0.333330005	0.066670001	0.083329998
0.133330002	0.066670001	0.083329998
0.533330023	0.066670001	0.083329998
0.733330011	0.066670001	0.083329998
0.933329999	0.066670001	0.083329998
0.800000012	0.200000003	0.083329998
0.200000003	0.200000003	0.083329998
0.400000006	0.200000003	0.083329998
0.000000000	0.200000003	0.083329998
0.600000024	0.200000003	0.083329998
0.133330002	0.266669989	0.083329998
0.733330011	0.266669989	0.083329998
0.933329999	0.266669989	0.083329998
0.333330005	0.266669989	0.083329998
0.533330023	0.266669989	0.083329998
0.000000000	0.400000006	0.083329998
0.600000024	0.400000006	0.083329998
0.800000012	0.400000006	0.083329998
0.200000003	0.400000006	0.083329998
0.400000006	0.400000006	0.083329998
0.133330002	0.466670007	0.083329998
0.733330011	0.466670007	0.083329998
0.933329999	0.466670007	0.083329998
0.333330005	0.466670007	0.083329998
0.533330023	0.466670007	0.083329998
0.000000000	0.600000024	0.083329998
0.400000006	0.600000024	0.083329998
0.800000012	0.600000024	0.083329998
0.600000024	0.600000024	0.083329998
0.200000003	0.600000024	0.083329998

0.133330002	0.666670024	0.083329998
0.933329999	0.666670024	0.083329998
0.533330023	0.666670024	0.083329998
0.733330011	0.666670024	0.083329998
0.333330005	0.666670024	0.083329998
0.400000006	0.800000012	0.083329998
0.600000024	0.800000012	0.083329998
0.000000000	0.800000012	0.083329998
0.800000012	0.800000012	0.083329998
0.200000003	0.800000012	0.083329998
0.133330002	0.866670012	0.083329998
0.933329999	0.866670012	0.083329998
0.533330023	0.866670012	0.083329998
0.733330011	0.866670012	0.083329998
0.333330005	0.866670012	0.083329998
0.200000003	0.000000000	0.083329998
0.400000006	0.000000000	0.083329998
0.600000024	0.000000000	0.083329998
0.800000012	0.000000000	0.083329998
0.000000000	0.000000000	0.250000000
0.200000003	0.000000000	0.250000000
0.066670001	0.133330002	0.250000000
0.266669989	0.133330002	0.250000000
0.466670007	0.133330002	0.250000000
0.666670024	0.133330002	0.250000000
0.866670012	0.133330002	0.250000000
0.600000024	0.200000003	0.250000000
0.800000012	0.200000003	0.250000000
0.200000003	0.200000003	0.250000000
0.400000006	0.200000003	0.250000000

0.000000000	0.200000003	0.250000000
0.666670024	0.333330005	0.250000000
0.866670012	0.333330005	0.250000000
0.266669989	0.333330005	0.250000000
0.466670007	0.333330005	0.250000000
0.066670001	0.333330005	0.250000000
0.000000000	0.400000006	0.250000000
0.600000024	0.400000006	0.250000000
0.800000012	0.400000006	0.250000000
0.200000003	0.400000006	0.250000000
0.400000006	0.400000006	0.250000000
0.066670001	0.533330023	0.250000000
0.666670024	0.533330023	0.250000000
0.866670012	0.533330023	0.250000000
0.266669989	0.533330023	0.250000000
0.466670007	0.533330023	0.250000000
0.400000006	0.600000024	0.250000000
0.000000000	0.600000024	0.250000000
0.600000024	0.600000024	0.250000000
0.800000012	0.600000024	0.250000000
0.200000003	0.600000024	0.250000000
0.066670001	0.733330011	0.250000000
0.866670012	0.733330011	0.250000000
0.466670007	0.733330011	0.250000000
0.666670024	0.733330011	0.250000000
0.266669989	0.733330011	0.250000000
0.400000006	0.800000012	0.250000000
0.600000024	0.800000012	0.250000000
0.000000000	0.800000012	0.250000000
0.800000012	0.800000012	0.250000000

0.200000003	0.800000012	0.250000000
0.866670012	0.933329999	0.250000000
0.266669989	0.933329999	0.250000000
0.066670001	0.933329999	0.250000000
0.466670007	0.933329999	0.250000000
0.666670024	0.933329999	0.250000000
0.600000024	0.000000000	0.250000000
0.800000012	0.000000000	0.250000000
0.400000006	0.000000000	0.250000000
0.611679971	0.415080011	0.381460011
0.743619978	0.475569993	0.380980015
0.811029971	0.609250009	0.380620003
0.604709983	0.210109994	0.381229997
0.737909973	0.273299992	0.381119996
0.541329980	0.282339990	0.381639987
0.806940019	0.407180011	0.381170005
0.941330016	0.675620019	0.381269991
0.404130012	0.010780000	0.381520003
0.537490010	0.075460002	0.380899996
0.339170009	0.080389999	0.381870002
0.939599991	0.474159986	0.381350011
0.005980000	0.607240021	0.381559998
0.800149977	0.797290027	0.380400002
0.006160000	0.808979988	0.381370008
0.204050004	0.809080005	0.381909996
0.935540020	0.870869994	0.381000012
0.139060006	0.876789987	0.381850004
0.002200000	0.005570000	0.381179988
0.602249980	0.005590000	0.380380005
0.205270007	0.010510000	0.381870002

0.735849977	0.070370004	0.380569994
0.802760005	0.204429999	0.380939990
0.406049997	0.214949995	0.381660014
0.935949981	0.272119999	0.380970001
0.003230000	0.405490011	0.381139994
0.137989998	0.675790012	0.381650001
0.337339997	0.876929998	0.381599993
0.799589992	0.999899983	0.380580008
0.934029996	0.069969997	0.380939990
0.137170002	0.075240001	0.381520003
0.001270000	0.204410002	0.380989999
0.135590002	0.474159986	0.380899996
0.201670006	0.607270002	0.381060004
0.401849985	0.808359981	0.380879998
0.731580019	0.863839984	0.380290002
0.134509996	0.272940010	0.380910009
0.334120005	0.674470007	0.380560011
0.534759998	0.871439993	0.380329996
0.204380006	0.209979996	0.381159991
0.339459985	0.282499999	0.380939990
0.201110005	0.406899989	0.380549997
0.596289992	0.797800004	0.379909992
0.332670003	0.473839998	0.379949987
0.398730010	0.607819974	0.379790008
0.562359989	0.543020010	0.523840010
0.824169993	0.720510006	0.590340018
0.528330028	0.730589986	0.593930006
0.709999979	0.589020014	0.596639991
0.510930002	0.600319982	0.604510009
0.740849972	0.667680025	0.380149990

0.401369989	0.413109988	0.379949987
0.527620018	0.669160008	0.379660010
0.595499992	0.575510025	0.573870003
0.647239983	0.526459992	0.496760011
0.464170009	0.529120028	0.504760027
0.934490025	0.728089988	0.612070024
0.428460002	0.744029999	0.615100026
0.613420010	0.038600001	0.518710017
0.569350004	0.539409995	0.380180001

Optimized POSCAR of NaRCO adsorbed on NiN_3C_1

NaRCO

1.0

12.3386201859	0.0000000000	0.0000000000
-6.1693115158	10.6855577071	0.0000000000
0.0000000000	0.0000000000	26.0551109314

H	C	N	O	Na	Ni
10	150	4	3	1	1

Direct

0.738629997	0.502860010	0.470340014
0.905470014	0.724120021	0.477310002
0.632099986	0.785279989	0.518980026
0.737089992	0.484840006	0.538030028
0.894590020	0.719070017	0.545499980
0.780210018	0.833440006	0.547890007
0.967750013	0.581170022	0.544219971
0.544030011	0.583859980	0.573029995
0.701860011	0.628260016	0.588320017
0.557969987	0.783379972	0.600549996
0.000000000	0.000000000	0.083329998
0.333330005	0.066670001	0.083329998

0.133330002	0.066670001	0.083329998
0.533330023	0.066670001	0.083329998
0.733330011	0.066670001	0.083329998
0.933329999	0.066670001	0.083329998
0.000000000	0.200000003	0.083329998
0.600000024	0.200000003	0.083329998
0.800000012	0.200000003	0.083329998
0.200000003	0.200000003	0.083329998
0.400000006	0.200000003	0.083329998
0.133330002	0.266669989	0.083329998
0.733330011	0.266669989	0.083329998
0.933329999	0.266669989	0.083329998
0.333330005	0.266669989	0.083329998
0.533330023	0.266669989	0.083329998
0.000000000	0.400000006	0.083329998
0.600000024	0.400000006	0.083329998
0.800000012	0.400000006	0.083329998
0.200000003	0.400000006	0.083329998
0.400000006	0.400000006	0.083329998
0.133330002	0.466670007	0.083329998
0.733330011	0.466670007	0.083329998
0.933329999	0.466670007	0.083329998
0.333330005	0.466670007	0.083329998
0.533330023	0.466670007	0.083329998
0.000000000	0.600000024	0.083329998
0.400000006	0.600000024	0.083329998
0.800000012	0.600000024	0.083329998
0.600000024	0.600000024	0.083329998
0.200000003	0.600000024	0.083329998
0.133330002	0.666670024	0.083329998

0.933329999	0.666670024	0.083329998
0.533330023	0.666670024	0.083329998
0.733330011	0.666670024	0.083329998
0.333330005	0.666670024	0.083329998
0.400000006	0.800000012	0.083329998
0.600000024	0.800000012	0.083329998
0.000000000	0.800000012	0.083329998
0.800000012	0.800000012	0.083329998
0.200000003	0.800000012	0.083329998
0.133330002	0.866670012	0.083329998
0.933329999	0.866670012	0.083329998
0.533330023	0.866670012	0.083329998
0.733330011	0.866670012	0.083329998
0.333330005	0.866670012	0.083329998
0.200000003	0.000000000	0.083329998
0.400000006	0.000000000	0.083329998
0.600000024	0.000000000	0.083329998
0.800000012	0.000000000	0.083329998
0.000000000	0.000000000	0.250000000
0.200000003	0.000000000	0.250000000
0.066670001	0.133330002	0.250000000
0.266669989	0.133330002	0.250000000
0.466670007	0.133330002	0.250000000
0.666670024	0.133330002	0.250000000
0.866670012	0.133330002	0.250000000
0.600000024	0.200000003	0.250000000
0.800000012	0.200000003	0.250000000
0.200000003	0.200000003	0.250000000
0.400000006	0.200000003	0.250000000
0.000000000	0.200000003	0.250000000

0.666670024	0.333330005	0.250000000
0.866670012	0.333330005	0.250000000
0.266669989	0.333330005	0.250000000
0.466670007	0.333330005	0.250000000
0.066670001	0.333330005	0.250000000
0.000000000	0.400000006	0.250000000
0.600000024	0.400000006	0.250000000
0.800000012	0.400000006	0.250000000
0.200000003	0.400000006	0.250000000
0.400000006	0.400000006	0.250000000
0.066670001	0.533330023	0.250000000
0.666670024	0.533330023	0.250000000
0.866670012	0.533330023	0.250000000
0.266669989	0.533330023	0.250000000
0.466670007	0.533330023	0.250000000
0.400000006	0.600000024	0.250000000
0.000000000	0.600000024	0.250000000
0.600000024	0.600000024	0.250000000
0.800000012	0.600000024	0.250000000
0.200000003	0.600000024	0.250000000
0.066670001	0.733330011	0.250000000
0.866670012	0.733330011	0.250000000
0.466670007	0.733330011	0.250000000
0.666670024	0.733330011	0.250000000
0.266669989	0.733330011	0.250000000
0.400000006	0.800000012	0.250000000
0.600000024	0.800000012	0.250000000
0.000000000	0.800000012	0.250000000
0.800000012	0.800000012	0.250000000
0.200000003	0.800000012	0.250000000

0.866670012	0.933329999	0.250000000
0.266669989	0.933329999	0.250000000
0.066670001	0.933329999	0.250000000
0.466670007	0.933329999	0.250000000
0.666670024	0.933329999	0.250000000
0.400000006	0.000000000	0.250000000
0.600000024	0.000000000	0.250000000
0.800000012	0.000000000	0.250000000
0.750119984	0.471120000	0.380129993
0.813600004	0.402539998	0.378390014
0.946340024	0.469509989	0.377119988
0.012970000	0.602639973	0.376870006
0.817879975	0.605009973	0.379110008
0.010160000	0.400920004	0.377539992
0.145060003	0.670949996	0.377290010
0.948599994	0.671060026	0.377499998
0.942889988	0.267520010	0.378560007
0.744960010	0.268759996	0.379610002
0.618879974	0.410039991	0.385749996
0.142719999	0.469440013	0.377629995
0.209030002	0.602569997	0.377490014
0.013360000	0.804109991	0.378589988
0.809809983	0.199910000	0.379119992
0.611790001	0.205540001	0.381249994
0.211180001	0.804270029	0.378410012
0.146249995	0.871940017	0.379009992
0.008280000	0.199729994	0.379509985
0.208360001	0.402229995	0.378520012
0.943050027	0.866400003	0.380140007
0.344529986	0.872030020	0.379469991

0.009480000	0.001050000	0.380409986
0.411390007	0.005950000	0.380789995
0.941200018	0.065420002	0.380499989
0.544659972	0.070809998	0.380780011
0.141570002	0.268249989	0.379530013
0.548219979	0.277240008	0.383980006
0.341569990	0.669730008	0.378399998
0.609480023	0.001060000	0.380320013
0.212329999	0.005650000	0.380430013
0.742919981	0.065729998	0.380070001
0.144229993	0.070469998	0.380530000
0.346179992	0.075360000	0.381700009
0.808109999	0.793179989	0.381819993
0.409099996	0.803489983	0.379379988
0.806750000	0.995369971	0.381020010
0.211160004	0.204929993	0.380710006
0.412900001	0.209810004	0.383619994
0.340229988	0.468870014	0.379559994
0.739009976	0.859549999	0.382450014
0.541999996	0.866850019	0.380389988
0.406760007	0.603340030	0.379689991
0.345950007	0.277200013	0.382299989
0.603690028	0.793420017	0.381900012
0.560249984	0.551400006	0.474539995
0.750599980	0.551580012	0.506879985
0.884150028	0.662800014	0.510909975
0.678409994	0.775370002	0.553449988
0.641669989	0.637619972	0.559629977
0.749170005	0.664040029	0.382380009
0.407350004	0.407029986	0.382250011

0.535030007 0.665199995 0.382640004
0.653680027 0.585590005 0.510580003
0.464899987 0.556190014 0.486860007
0.974420011 0.621940017 0.511349976
0.648940027 0.823989987 0.598089993
0.797510028 0.136260003 0.516369998
0.576479971 0.534300029 0.393049985

Optimized POSCAR of $NaCO_2$ adsorbed on NiN_3C_1

NaCO2
1.0
12.3386201859 0.0000000000 0.0000000000
-6.1693115158 10.6855577071 0.0000000000
0.0000000000 0.0000000000 26.0551109314
C N O Na Ni
146 3 2 1 1
Direct
0.000000000 0.000000000 0.083329998
0.533330023 0.066670001 0.083329998
0.733330011 0.066670001 0.083329998
0.933329999 0.066670001 0.083329998
0.333330005 0.066670001 0.083329998
0.133330002 0.066670001 0.083329998
0.800000012 0.200000003 0.083329998
0.200000003 0.200000003 0.083329998
0.400000006 0.200000003 0.083329998
0.000000000 0.200000003 0.083329998
0.600000024 0.200000003 0.083329998
0.133330002 0.266669989 0.083329998
0.733330011 0.266669989 0.083329998
0.933329999 0.266669989 0.083329998

0.333330005	0.266669989	0.083329998
0.533330023	0.266669989	0.083329998
0.000000000	0.400000006	0.083329998
0.600000024	0.400000006	0.083329998
0.800000012	0.400000006	0.083329998
0.200000003	0.400000006	0.083329998
0.400000006	0.400000006	0.083329998
0.133330002	0.466670007	0.083329998
0.733330011	0.466670007	0.083329998
0.933329999	0.466670007	0.083329998
0.333330005	0.466670007	0.083329998
0.533330023	0.466670007	0.083329998
0.000000000	0.600000024	0.083329998
0.400000006	0.600000024	0.083329998
0.800000012	0.600000024	0.083329998
0.600000024	0.600000024	0.083329998
0.200000003	0.600000024	0.083329998
0.133330002	0.666670024	0.083329998
0.933329999	0.666670024	0.083329998
0.533330023	0.666670024	0.083329998
0.733330011	0.666670024	0.083329998
0.333330005	0.666670024	0.083329998
0.400000006	0.800000012	0.083329998
0.600000024	0.800000012	0.083329998
0.000000000	0.800000012	0.083329998
0.800000012	0.800000012	0.083329998
0.200000003	0.800000012	0.083329998
0.133330002	0.866670012	0.083329998
0.933329999	0.866670012	0.083329998
0.533330023	0.866670012	0.083329998

0.733330011	0.866670012	0.083329998
0.333330005	0.866670012	0.083329998
0.200000003	0.000000000	0.083329998
0.400000006	0.000000000	0.083329998
0.600000024	0.000000000	0.083329998
0.800000012	0.000000000	0.083329998
0.000000000	0.000000000	0.250000000
0.066670001	0.133330002	0.250000000
0.266669989	0.133330002	0.250000000
0.466670007	0.133330002	0.250000000
0.666670024	0.133330002	0.250000000
0.866670012	0.133330002	0.250000000
0.600000024	0.200000003	0.250000000
0.800000012	0.200000003	0.250000000
0.200000003	0.200000003	0.250000000
0.400000006	0.200000003	0.250000000
0.000000000	0.200000003	0.250000000
0.666670024	0.333330005	0.250000000
0.866670012	0.333330005	0.250000000
0.266669989	0.333330005	0.250000000
0.466670007	0.333330005	0.250000000
0.066670001	0.333330005	0.250000000
0.000000000	0.400000006	0.250000000
0.600000024	0.400000006	0.250000000
0.800000012	0.400000006	0.250000000
0.200000003	0.400000006	0.250000000
0.400000006	0.400000006	0.250000000
0.066670001	0.533330023	0.250000000
0.666670024	0.533330023	0.250000000
0.866670012	0.533330023	0.250000000

0.266669989	0.533330023	0.250000000
0.466670007	0.533330023	0.250000000
0.400000006	0.600000024	0.250000000
0.000000000	0.600000024	0.250000000
0.600000024	0.600000024	0.250000000
0.800000012	0.600000024	0.250000000
0.200000003	0.600000024	0.250000000
0.066670001	0.733330011	0.250000000
0.866670012	0.733330011	0.250000000
0.466670007	0.733330011	0.250000000
0.666670024	0.733330011	0.250000000
0.266669989	0.733330011	0.250000000
0.400000006	0.800000012	0.250000000
0.600000024	0.800000012	0.250000000
0.000000000	0.800000012	0.250000000
0.800000012	0.800000012	0.250000000
0.200000003	0.800000012	0.250000000
0.266669989	0.933329999	0.250000000
0.066670001	0.933329999	0.250000000
0.466670007	0.933329999	0.250000000
0.666670024	0.933329999	0.250000000
0.866670012	0.933329999	0.250000000
0.600000024	0.000000000	0.250000000
0.800000012	0.000000000	0.250000000
0.200000003	0.000000000	0.250000000
0.400000006	0.000000000	0.250000000
0.796559989	0.790759981	0.381330013
0.592559993	0.791410029	0.381709993
0.727599978	0.856859982	0.381099999
0.795480013	0.992699981	0.381080002

0.929880023	0.062710002	0.381399989
0.731549978	0.063000001	0.381440014
0.133039996	0.068039998	0.382420003
0.533479989	0.068269998	0.382429987
0.199800000	0.202439994	0.382050008
0.600719988	0.202559993	0.382640004
0.401789993	0.207420006	0.383320004
0.536729991	0.274520010	0.383839995
0.335070014	0.274780005	0.382279992
0.607429981	0.406769991	0.384869993
0.328669995	0.466399997	0.380970001
0.739080012	0.468320012	0.382499993
0.395080000	0.600549996	0.381249994
0.806280017	0.601939976	0.381870002
0.931289971	0.863650024	0.381850004
0.530910015	0.864180028	0.381790012
0.998170018	0.998390019	0.381920010
0.598249972	0.998499990	0.381830007
0.201240003	0.003330000	0.382800013
0.400160015	0.003650000	0.382829994
0.334950000	0.072760001	0.383060008
0.996980011	0.197070003	0.381410003
0.798550010	0.197090000	0.381619990
0.931599975	0.264620006	0.381410003
0.130280003	0.265540004	0.381500006
0.733609974	0.265870005	0.382120013
0.998830020	0.398039997	0.381619990
0.196789995	0.399379998	0.381280005
0.802489996	0.399740010	0.381900012
0.934939981	0.466399997	0.381879985

0.131229997 0.466360003 0.381610006
0.329869986 0.667060018 0.381489992
0.937039971 0.668089986 0.382250011
0.397619992 0.800939977 0.382030010
0.002100000 0.801509976 0.382319987
0.199919999 0.801649988 0.382609993
0.134729996 0.869300008 0.382679999
0.333249986 0.869390011 0.382569999
0.001460000 0.599860013 0.382290006
0.197640002 0.599829972 0.381790012
0.133680001 0.668210030 0.382290006
0.569989979 0.535019994 0.469909996
0.736940026 0.660579979 0.381130010
0.396730006 0.404960006 0.381029993
0.523350000 0.661939979 0.382409990
0.470290005 0.521889985 0.486440003
0.669589996 0.552280009 0.488319993
0.763759971 0.878459990 0.517239988
0.564890027 0.531520009 0.388650000

Optimized POSCAR of NaCOOH adsorbed on NiN_3C_1

HCNONaNi
1.0
12.3386201859 0.0000000000 0.0000000000
-6.1693115158 10.6855577071 0.0000000000
0.0000000000 0.0000000000 26.0551109314
H C N O Na Ni
1 146 3 2 1 1
Direct
0.636600018 0.511219978 0.532720029
0.000000000 0.000000000 0.083329998

0.533330023	0.066670001	0.083329998
0.733330011	0.066670001	0.083329998
0.933329999	0.066670001	0.083329998
0.333330005	0.066670001	0.083329998
0.133330002	0.066670001	0.083329998
0.400000006	0.200000003	0.083329998
0.000000000	0.200000003	0.083329998
0.600000024	0.200000003	0.083329998
0.800000012	0.200000003	0.083329998
0.200000003	0.200000003	0.083329998
0.133330002	0.266669989	0.083329998
0.733330011	0.266669989	0.083329998
0.933329999	0.266669989	0.083329998
0.333330005	0.266669989	0.083329998
0.533330023	0.266669989	0.083329998
0.000000000	0.400000006	0.083329998
0.600000024	0.400000006	0.083329998
0.800000012	0.400000006	0.083329998
0.200000003	0.400000006	0.083329998
0.400000006	0.400000006	0.083329998
0.133330002	0.466670007	0.083329998
0.733330011	0.466670007	0.083329998
0.933329999	0.466670007	0.083329998
0.333330005	0.466670007	0.083329998
0.533330023	0.466670007	0.083329998
0.000000000	0.600000024	0.083329998
0.400000006	0.600000024	0.083329998
0.800000012	0.600000024	0.083329998
0.600000024	0.600000024	0.083329998
0.200000003	0.600000024	0.083329998

0.133330002	0.666670024	0.083329998
0.933329999	0.666670024	0.083329998
0.533330023	0.666670024	0.083329998
0.733330011	0.666670024	0.083329998
0.333330005	0.666670024	0.083329998
0.400000006	0.800000012	0.083329998
0.600000024	0.800000012	0.083329998
0.000000000	0.800000012	0.083329998
0.800000012	0.800000012	0.083329998
0.200000003	0.800000012	0.083329998
0.133330002	0.866670012	0.083329998
0.933329999	0.866670012	0.083329998
0.533330023	0.866670012	0.083329998
0.733330011	0.866670012	0.083329998
0.333330005	0.866670012	0.083329998
0.200000003	0.000000000	0.083329998
0.400000006	0.000000000	0.083329998
0.600000024	0.000000000	0.083329998
0.800000012	0.000000000	0.083329998
0.000000000	0.000000000	0.250000000
0.066670001	0.133330002	0.250000000
0.266669989	0.133330002	0.250000000
0.466670007	0.133330002	0.250000000
0.666670024	0.133330002	0.250000000
0.866670012	0.133330002	0.250000000
0.600000024	0.200000003	0.250000000
0.800000012	0.200000003	0.250000000
0.200000003	0.200000003	0.250000000
0.400000006	0.200000003	0.250000000
0.000000000	0.200000003	0.250000000

0.666670024	0.333330005	0.250000000
0.866670012	0.333330005	0.250000000
0.266669989	0.333330005	0.250000000
0.466670007	0.333330005	0.250000000
0.066670001	0.333330005	0.250000000
0.000000000	0.400000006	0.250000000
0.600000024	0.400000006	0.250000000
0.800000012	0.400000006	0.250000000
0.200000003	0.400000006	0.250000000
0.400000006	0.400000006	0.250000000
0.066670001	0.533330023	0.250000000
0.666670024	0.533330023	0.250000000
0.866670012	0.533330023	0.250000000
0.266669989	0.533330023	0.250000000
0.466670007	0.533330023	0.250000000
0.400000006	0.600000024	0.250000000
0.000000000	0.600000024	0.250000000
0.600000024	0.600000024	0.250000000
0.800000012	0.600000024	0.250000000
0.200000003	0.600000024	0.250000000
0.066670001	0.733330011	0.250000000
0.866670012	0.733330011	0.250000000
0.466670007	0.733330011	0.250000000
0.666670024	0.733330011	0.250000000
0.266669989	0.733330011	0.250000000
0.400000006	0.800000012	0.250000000
0.600000024	0.800000012	0.250000000
0.000000000	0.800000012	0.250000000
0.800000012	0.800000012	0.250000000
0.200000003	0.800000012	0.250000000

0.266669989	0.933329999	0.250000000
0.066670001	0.933329999	0.250000000
0.466670007	0.933329999	0.250000000
0.666670024	0.933329999	0.250000000
0.866670012	0.933329999	0.250000000
0.800000012	0.000000000	0.250000000
0.200000003	0.000000000	0.250000000
0.400000006	0.000000000	0.250000000
0.600000024	0.000000000	0.250000000
0.201550007	0.002890000	0.383300006
0.400579989	0.003270000	0.383679986
0.930180013	0.062470000	0.381960005
0.732140005	0.062909998	0.382330000
0.133300006	0.067649998	0.382869989
0.533800006	0.068080001	0.383350015
0.335379988	0.072650000	0.384030014
0.601000011	0.202779993	0.383390009
0.199900001	0.201940000	0.382459998
0.401980013	0.207039997	0.384660006
0.537240028	0.274349988	0.384860009
0.334639996	0.274190009	0.383139998
0.608120024	0.407000005	0.385749996
0.797540009	0.790579975	0.382169992
0.592809975	0.790579975	0.383089989
0.728089988	0.856660008	0.382510006
0.932330012	0.863730013	0.382160008
0.531189978	0.864080012	0.382669985
0.795840025	0.992420018	0.382090002
0.598659992	0.998189986	0.382779986
0.998589993	0.998279989	0.382340014

0.997200012	0.196830004	0.381669998
0.798900008	0.197060004	0.382169992
0.931879997	0.264620006	0.381669998
0.130349994	0.265269995	0.381599993
0.734179974	0.266030014	0.382569999
0.328759998	0.465869993	0.381480008
0.739499986	0.468450010	0.382589996
0.395339996	0.600210011	0.382349998
0.807309985	0.602330029	0.381599993
0.398240000	0.800710022	0.382710010
0.002680000	0.801400006	0.382070005
0.333790004	0.869279981	0.383120000
0.135560006	0.869300008	0.382679999
0.999180019	0.398030013	0.381570011
0.196980000	0.399170011	0.381220013
0.802850008	0.399710000	0.381909996
0.935509980	0.466690004	0.381550014
0.131630003	0.466529995	0.381460011
0.002250000	0.599839985	0.381729990
0.198029995	0.599680007	0.381770015
0.330469996	0.666880012	0.382050008
0.134279996	0.668190002	0.382099986
0.938059986	0.668410003	0.381619990
0.200430006	0.801549971	0.382699996
0.559390008	0.524030030	0.470479995
0.395680010	0.403659999	0.382230014
0.738709986	0.661589980	0.382140011
0.523880005	0.662180007	0.384299994
0.653219998	0.514630020	0.495469987
0.476819992	0.525349975	0.496850014

0.756609976 0.855459988 0.519699991

0.565439999 0.530960023 0.392589986

Optimized POSCAR of NaCO adsorbed on NiN_3C_1

CNONaNi

1.0

12.3386201859	0.0000000000	0.0000000000
-6.1693115158	10.6855577071	0.0000000000
0.0000000000	0.0000000000	26.0551109314

C	N	O	Na	Ni
146	3	1	1	1

Direct

0.000000000	0.000000000	0.083329998
0.533330023	0.066670001	0.083329998
0.733330011	0.066670001	0.083329998
0.933329999	0.066670001	0.083329998
0.333330005	0.066670001	0.083329998
0.133330002	0.066670001	0.083329998
0.400000006	0.200000003	0.083329998
0.000000000	0.200000003	0.083329998
0.600000024	0.200000003	0.083329998
0.800000012	0.200000003	0.083329998
0.200000003	0.200000003	0.083329998
0.133330002	0.266669989	0.083329998
0.733330011	0.266669989	0.083329998
0.933329999	0.266669989	0.083329998
0.333330005	0.266669989	0.083329998
0.533330023	0.266669989	0.083329998
0.000000000	0.400000006	0.083329998
0.600000024	0.400000006	0.083329998
0.800000012	0.400000006	0.083329998

0.200000003	0.400000006	0.083329998
0.400000006	0.400000006	0.083329998
0.133330002	0.466670007	0.083329998
0.733330011	0.466670007	0.083329998
0.933329999	0.466670007	0.083329998
0.333330005	0.466670007	0.083329998
0.533330023	0.466670007	0.083329998
0.000000000	0.600000024	0.083329998
0.400000006	0.600000024	0.083329998
0.800000012	0.600000024	0.083329998
0.600000024	0.600000024	0.083329998
0.200000003	0.600000024	0.083329998
0.133330002	0.666670024	0.083329998
0.933329999	0.666670024	0.083329998
0.533330023	0.666670024	0.083329998
0.733330011	0.666670024	0.083329998
0.333330005	0.666670024	0.083329998
0.400000006	0.800000012	0.083329998
0.600000024	0.800000012	0.083329998
0.000000000	0.800000012	0.083329998
0.800000012	0.800000012	0.083329998
0.200000003	0.800000012	0.083329998
0.133330002	0.866670012	0.083329998
0.933329999	0.866670012	0.083329998
0.533330023	0.866670012	0.083329998
0.733330011	0.866670012	0.083329998
0.333330005	0.866670012	0.083329998
0.200000003	0.000000000	0.083329998
0.400000006	0.000000000	0.083329998
0.600000024	0.000000000	0.083329998

0.800000012	0.000000000	0.083329998
0.000000000	0.000000000	0.250000000
0.200000003	0.000000000	0.250000000
0.066670001	0.133330002	0.250000000
0.266669989	0.133330002	0.250000000
0.466670007	0.133330002	0.250000000
0.666670024	0.133330002	0.250000000
0.866670012	0.133330002	0.250000000
0.600000024	0.200000003	0.250000000
0.800000012	0.200000003	0.250000000
0.200000003	0.200000003	0.250000000
0.400000006	0.200000003	0.250000000
0.000000000	0.200000003	0.250000000
0.666670024	0.333330005	0.250000000
0.866670012	0.333330005	0.250000000
0.266669989	0.333330005	0.250000000
0.466670007	0.333330005	0.250000000
0.066670001	0.333330005	0.250000000
0.000000000	0.400000006	0.250000000
0.600000024	0.400000006	0.250000000
0.800000012	0.400000006	0.250000000
0.200000003	0.400000006	0.250000000
0.400000006	0.400000006	0.250000000
0.066670001	0.533330023	0.250000000
0.666670024	0.533330023	0.250000000
0.866670012	0.533330023	0.250000000
0.266669989	0.533330023	0.250000000
0.466670007	0.533330023	0.250000000
0.400000006	0.600000024	0.250000000
0.000000000	0.600000024	0.250000000

0.600000024	0.600000024	0.250000000
0.800000012	0.600000024	0.250000000
0.200000003	0.600000024	0.250000000
0.066670001	0.733330011	0.250000000
0.866670012	0.733330011	0.250000000
0.466670007	0.733330011	0.250000000
0.666670024	0.733330011	0.250000000
0.266669989	0.733330011	0.250000000
0.400000006	0.800000012	0.250000000
0.600000024	0.800000012	0.250000000
0.000000000	0.800000012	0.250000000
0.800000012	0.800000012	0.250000000
0.200000003	0.800000012	0.250000000
0.266669989	0.933329999	0.250000000
0.066670001	0.933329999	0.250000000
0.466670007	0.933329999	0.250000000
0.666670024	0.933329999	0.250000000
0.866670012	0.933329999	0.250000000
0.800000012	0.000000000	0.250000000
0.400000006	0.000000000	0.250000000
0.600000024	0.000000000	0.250000000
0.201350003	0.003070000	0.382889986
0.400350004	0.003340000	0.382910013
0.930090010	0.062490001	0.381900012
0.732029974	0.062870003	0.382010013
0.133220002	0.067790002	0.382169992
0.533729970	0.068089999	0.382230014
0.335269988	0.072910003	0.382880002
0.600960016	0.202710003	0.382180005
0.200310007	0.202419996	0.381870002

0.402040005	0.207369998	0.382319987
0.537419975	0.274780005	0.382110000
0.335319996	0.274809986	0.381509990
0.607800007	0.407059997	0.381929994
0.796440005	0.789940000	0.380989999
0.592419982	0.790489972	0.380769998
0.727769971	0.856469989	0.380699992
0.931729972	0.863439977	0.381700009
0.530910015	0.864139974	0.381579995
0.795669973	0.992410004	0.381440014
0.598429978	0.998210013	0.381839991
0.998260021	0.998099983	0.381799996
0.997259974	0.196850002	0.382580012
0.798850000	0.196830004	0.382759988
0.932039976	0.264640003	0.383089989
0.130449995	0.265390009	0.382299989
0.734139979	0.265830010	0.382649988
0.328740001	0.466300011	0.381410003
0.739530027	0.468080014	0.381989986
0.394950002	0.600489974	0.381460011
0.807030022	0.601849973	0.381940007
0.398009986	0.800979972	0.382580012
0.002240000	0.801460028	0.382770002
0.200189993	0.801699996	0.383920014
0.333579987	0.869589984	0.383399993
0.135220006	0.869390011	0.383430004
0.999189973	0.397929996	0.383690000
0.197129995	0.399370015	0.382519990
0.802980006	0.399659991	0.382820010
0.935590029	0.466650009	0.383729994

0.131630003	0.466650009	0.383540004
0.197789997	0.599829972	0.383679986
0.001990000	0.599749982	0.383899987
0.330190003	0.667119980	0.382600009
0.134110004	0.668420017	0.384110004
0.937420011	0.668160021	0.382990003
0.561420023	0.527440012	0.475259990
0.396780014	0.405030012	0.380519986
0.737380028	0.660600007	0.381320000
0.523720026	0.662220001	0.380789995
0.647379994	0.533949971	0.496780008
0.762109995	0.862389982	0.518400013
0.565140009	0.531610012	0.383870006

Optimized POSCAR of CO adsorbed on NiN_3C_1

CNONi

1.0

12.3386201859	0.0000000000	0.0000000000
-6.1693115158	10.6855577071	0.0000000000
0.0000000000	0.0000000000	26.0551109314

C N O Ni

146 3 1 1

Direct

0.000000000	0.000000000	0.083329998
0.533330023	0.066670001	0.083329998
0.733330011	0.066670001	0.083329998
0.933329999	0.066670001	0.083329998
0.333330005	0.066670001	0.083329998
0.133330002	0.066670001	0.083329998
0.400000006	0.200000003	0.083329998
0.000000000	0.200000003	0.083329998

0.600000024	0.200000003	0.083329998
0.800000012	0.200000003	0.083329998
0.200000003	0.200000003	0.083329998
0.133330002	0.266669989	0.083329998
0.733330011	0.266669989	0.083329998
0.933329999	0.266669989	0.083329998
0.333330005	0.266669989	0.083329998
0.533330023	0.266669989	0.083329998
0.000000000	0.400000006	0.083329998
0.600000024	0.400000006	0.083329998
0.800000012	0.400000006	0.083329998
0.200000003	0.400000006	0.083329998
0.400000006	0.400000006	0.083329998
0.133330002	0.466670007	0.083329998
0.733330011	0.466670007	0.083329998
0.933329999	0.466670007	0.083329998
0.333330005	0.466670007	0.083329998
0.533330023	0.466670007	0.083329998
0.000000000	0.600000024	0.083329998
0.400000006	0.600000024	0.083329998
0.800000012	0.600000024	0.083329998
0.600000024	0.600000024	0.083329998
0.200000003	0.600000024	0.083329998
0.133330002	0.666670024	0.083329998
0.933329999	0.666670024	0.083329998
0.533330023	0.666670024	0.083329998
0.733330011	0.666670024	0.083329998
0.333330005	0.666670024	0.083329998
0.400000006	0.800000012	0.083329998
0.600000024	0.800000012	0.083329998

0.000000000	0.800000012	0.083329998
0.800000012	0.800000012	0.083329998
0.200000003	0.800000012	0.083329998
0.133330002	0.866670012	0.083329998
0.933329999	0.866670012	0.083329998
0.533330023	0.866670012	0.083329998
0.733330011	0.866670012	0.083329998
0.333330005	0.866670012	0.083329998
0.200000003	0.000000000	0.083329998
0.400000006	0.000000000	0.083329998
0.600000024	0.000000000	0.083329998
0.800000012	0.000000000	0.083329998
0.000000000	0.000000000	0.250000000
0.200000003	0.000000000	0.250000000
0.066670001	0.133330002	0.250000000
0.266669989	0.133330002	0.250000000
0.466670007	0.133330002	0.250000000
0.666670024	0.133330002	0.250000000
0.866670012	0.133330002	0.250000000
0.600000024	0.200000003	0.250000000
0.800000012	0.200000003	0.250000000
0.200000003	0.200000003	0.250000000
0.400000006	0.200000003	0.250000000
0.000000000	0.200000003	0.250000000
0.666670024	0.333330005	0.250000000
0.866670012	0.333330005	0.250000000
0.266669989	0.333330005	0.250000000
0.466670007	0.333330005	0.250000000
0.066670001	0.333330005	0.250000000
0.000000000	0.400000006	0.250000000

0.600000024	0.400000006	0.250000000
0.800000012	0.400000006	0.250000000
0.200000003	0.400000006	0.250000000
0.400000006	0.400000006	0.250000000
0.066670001	0.533330023	0.250000000
0.666670024	0.533330023	0.250000000
0.866670012	0.533330023	0.250000000
0.266669989	0.533330023	0.250000000
0.466670007	0.533330023	0.250000000
0.400000006	0.600000024	0.250000000
0.000000000	0.600000024	0.250000000
0.600000024	0.600000024	0.250000000
0.800000012	0.600000024	0.250000000
0.200000003	0.600000024	0.250000000
0.066670001	0.733330011	0.250000000
0.866670012	0.733330011	0.250000000
0.466670007	0.733330011	0.250000000
0.666670024	0.733330011	0.250000000
0.266669989	0.733330011	0.250000000
0.400000006	0.800000012	0.250000000
0.600000024	0.800000012	0.250000000
0.000000000	0.800000012	0.250000000
0.800000012	0.800000012	0.250000000
0.200000003	0.800000012	0.250000000
0.266669989	0.933329999	0.250000000
0.066670001	0.933329999	0.250000000
0.466670007	0.933329999	0.250000000
0.666670024	0.933329999	0.250000000
0.866670012	0.933329999	0.250000000
0.800000012	0.000000000	0.250000000

0.400000006	0.000000000	0.250000000
0.600000024	0.000000000	0.250000000
0.201399997	0.003570000	0.382640004
0.400440007	0.003810000	0.382669985
0.929759979	0.062369999	0.382490009
0.731459975	0.062689997	0.382369995
0.133029997	0.067979999	0.382380009
0.533500016	0.068379998	0.382360011
0.335299999	0.073229998	0.383069992
0.600839972	0.202790007	0.382510006
0.199560001	0.202260002	0.382110000
0.402150005	0.207650006	0.383509994
0.537079990	0.274549991	0.383579999
0.334390014	0.274599999	0.382640004
0.608319998	0.407070011	0.384409994
0.796809971	0.790489972	0.382900000
0.592220008	0.791100025	0.382340014
0.727569997	0.856509984	0.383040011
0.931909978	0.863849998	0.382360011
0.530579984	0.864390016	0.381790012
0.795440018	0.992200017	0.382730007
0.598309994	0.998459995	0.382090002
0.998199999	0.998260021	0.382360011
0.996710002	0.196710005	0.382220000
0.798539996	0.196899995	0.382279992
0.931519985	0.264349997	0.382200003
0.130030006	0.265410006	0.381909996
0.733870029	0.265929997	0.382290006
0.328339994	0.465860009	0.381229997
0.739359975	0.468309999	0.382290006

0.395300001	0.601109982	0.381209999
0.807089984	0.602460027	0.382120013
0.397870004	0.801110029	0.381850004
0.002440000	0.801739991	0.382340014
0.200420007	0.802200019	0.382490009
0.333400011	0.869830012	0.382400006
0.135350004	0.869899988	0.382519990
0.999029994	0.398240000	0.382039994
0.196400002	0.399120003	0.381469995
0.802730024	0.399569988	0.381940007
0.935209990	0.466600001	0.381960005
0.131300002	0.466580003	0.381729990
0.197740003	0.599990010	0.381740004
0.001960000	0.600120008	0.382070005
0.329990000	0.667490005	0.381399989
0.133870006	0.668240011	0.382239997
0.937940001	0.668760002	0.382079989
0.577329993	0.533370018	0.468989998
0.395110011	0.404150009	0.382169992
0.738430023	0.661289990	0.383139998
0.522930026	0.662880003	0.382470012
0.642920017	0.527050018	0.499410003
0.564979970	0.531470001	0.391799986

References Cited in the Supporting Information

(1) Zhang, Z.; Gee, W.; Sautet, P.; Alexandrova, A. N. H and CO Co-Induced Roughening of Cu Surface in CO_2 Electroreduction Conditions. *J. Am. Chem. Soc.* **2024**, *146* (23), 16119–16127. https://doi.org/10.1021/jacs.4c03515.

(2) Choi, J.; Chiu, S.; Banerjee, A.; Sacci, R. L.; Veith, G. M.; Stieber, C.; Hahn, C.; Alexandrova, A. N.; Morales-Guio, C. G. Corrosion and Enhanced Hydrogen Evolution in Electrochemical Reduction of Ammonium Carbamate on Transition Metal Surfaces. *J. Phys. Chem. Lett.* **2024**, 8007–8017. https://doi.org/10.1021/acs.jpclett.4c01638.

(3) Zhang, Z.; Hermans, I.; Alexandrova, A. N. Off-Stoichiometric Restructuring and Sliding Dynamics of Hexagonal Boron Nitride Edges in Conditions of Oxidative Dehydrogenation of Propane. *J. Am. Chem. Soc.* **2023**, *145* (31), 17265–17273. https://doi.org/10.1021/jacs.3c04613.

(4) Steinmann, S. N.; Michel, C.; Schwiedernoch, R.; Sautet, P. Impacts of Electrode Potentials and Solvents on the Electroreduction of CO_2: A Comparison of Theoretical Approaches. *Phys. Chem. Chem. Phys.* **2015**, *17* (21), 13949–13963. https://doi.org/10.1039/C5CP00946D.

(5) Kowalski, R. M.; Banerjee, A.; Yue, C.; Gracia, S. G.; Cheng, D.; Morales-Guio, C. G.; Sautet, P. Electroreduction of Captured CO_2 on Silver Catalysts: Influence of the Capture Agent and Proton Source. *J. Am. Chem. Soc.* **2024**, *146* (30), 20728–20741. https://doi.org/10.1021/jacs.4c03915.

(6) Mathew, K.; Kolluru, V. S. C.; Mula, S.; Steinmann, S. N.; Hennig, R. G. Implicit Self-Consistent Electrolyte Model in Plane-Wave Density-Functional Theory. *The Journal of Chemical Physics* **2019**, *151* (23), 234101. https://doi.org/10.1063/1.5132354.

(7) Vilela Oliveira, D.; Laun, J.; Peintinger, M. F.; Bredow, T. BSSE-Correction Scheme for Consistent Gaussian Basis Sets of Double- and Triple-Zeta Valence with Polarization Quality for Solid-State Calculations. *Journal of Computational Chemistry* **2019**, *40* (27), 2364–2376. https://doi.org/10.1002/jcc.26013.

(8) Erba, A.; Desmarais, J. K.; Casassa, S.; Civalleri, B.; Donà, L.; Bush, I. J.; Searle, B.; Maschio, L.; Edith-Daga, L.; Cossard, A.; Ribaldone, C.; Ascrizzi, E.; Marana, N. L.; Flament, J.-P.; Kirtman, B. CRYSTAL23: A Program for Computational Solid State Physics and Chemistry. *J. Chem. Theory Comput.* **2023**, *19* (20), 6891–6932. https://doi.org/10.1021/acs.jctc.2c00958.

(9) Pisani, C.; Schütz, M.; Casassa, S.; Usvyat, D.; Maschio, L.; Lorenz, M.; Erba, A. CRYSCOR: A Program for the Post-Hartree–Fock Treatment of Periodic Systems. *Phys. Chem. Chem. Phys.* **2012**, *14* (21), 7615–7628. https://doi.org/10.1039/C2CP23927B.

(10) Weigend, F.; Köhn, A.; Hättig, C. Efficient Use of the Correlation Consistent Basis Sets in Resolution of the Identity MP2 Calculations. *The Journal of Chemical Physics* **2002**, *116* (8), 3175–3183. https://doi.org/10.1063/1.1445115.

(11) Knowles, P. J.; Handy, N. C. A Determinant Based Full Configuration Interaction Program. *Computer Physics Communications* **1989**, *54* (1), 75–83. https://doi.org/10.1016/0010-4655(89)90033-7.

(12) Sun, Q.; Zhang, X.; Banerjee, S.; Bao, P.; Barbry, M.; Blunt, N. S.; Bogdanov, N. A.; Booth, G. H.; Chen, J.; Cui, Z.-H.; Eriksen, J. J.; Gao, Y.; Guo, S.; Hermann, J.; Hermes, M. R.; Koh, K.; Koval, P.; Lehtola, S.; Li, Z.; Liu, J.; Mardirossian, N.; McClain, J. D.; Motta, M.; Mussard, B.; Pham, H. Q.; Pulkin, A.; Purwanto, W.; Robinson, P. J.; Ronca, E.;

Sayfutyarova, E. R.; Scheurer, M.; Schurkus, H. F.; Smith, J. E. T.; Sun, C.; Sun, S.-N.; Upadhyay, S.; Wagner, L. K.; Wang, X.; White, A.; Whitfield, J. D.; Williamson, M. J.; Wouters, S.; Yang, J.; Yu, J. M.; Zhu, T.; Berkelbach, T. C.; Sharma, S.; Sokolov, A. Yu.; Chan, G. K.-L. Recent Developments in the PySCF Program Package. *The Journal of Chemical Physics* **2020**, *153* (2), 024109. https://doi.org/10.1063/5.0006074.
(13) Celani, P.; Werner, H.-J. Multireference Perturbation Theory for Large Restricted and Selected Active Space Reference Wave Functions. *The Journal of Chemical Physics* **2000**, *112* (13), 5546–5557. https://doi.org/10.1063/1.481132.
(14) Werner, H.-J.; Knowles, P. J.; Manby, F. R.; Black, J. A.; Doll, K.; Heßelmann, A.; Kats, D.; Köhn, A.; Korona, T.; Kreplin, D. A.; Ma, Q.; Miller, T. F., III; Mitrushchenkov, A.; Peterson, K. A.; Polyak, I.; Rauhut, G.; Sibaev, M. The Molpro Quantum Chemistry Package. *The Journal of Chemical Physics* **2020**, *152* (14), 144107. https://doi.org/10.1063/5.0005081.
(15) Radoń, M. Spin-State Energetics of Heme-Related Models from DFT and Coupled Cluster Calculations. *J. Chem. Theory Comput.* **2014**, *10* (6), 2306–2321. https://doi.org/10.1021/ct500103h.
(16) Lee, T. J.; Taylor, P. R. A Diagnostic for Determining the Quality of Single-Reference Electron Correlation Methods. *International Journal of Quantum Chemistry* **1989**, *36* (S23), 199–207. https://doi.org/10.1002/qua.560360824.
(17) Lee, T. J.; Rice, J. E.; Scuseria, G. E.; Schaefer, H. F. Theoretical Investigations of Molecules Composed Only of Fluorine, Oxygen and Nitrogen: Determination of the Equilibrium Structures of FOOF, (NO)2 and FNNF and the Transition State Structure for FNNF Cis-Trans Isomerization. *Theoret. Chim. Acta* **1989**, *75* (2), 81–98. https://doi.org/10.1007/BF00527711.
(18) Ali, Md. E.; Sanyal, B.; Oppeneer, P. M. Electronic Structure, Spin-States, and Spin-Crossover Reaction of Heme-Related Fe-Porphyrins: A Theoretical Perspective. *J. Phys. Chem. B* **2012**, *116* (20), 5849–5859. https://doi.org/10.1021/jp3021563.
(19) Kozuch, S.; Shaik, S. A Combined Kinetic−Quantum Mechanical Model for Assessment of Catalytic Cycles: Application to Cross-Coupling and Heck Reactions. *J. Am. Chem. Soc.* **2006**, *128* (10), 3355–3365. https://doi.org/10.1021/ja0559146.
(20) Kozuch, S.; Shaik, S. How to Conceptualize Catalytic Cycles? The Energetic Span Model. *Acc. Chem. Res.* **2011**, *44* (2), 101–110. https://doi.org/10.1021/ar1000956.
(21) Joly, Y. X-Ray Absorption near-Edge Structure Calculations beyond the Muffin-Tin Approximation. *Phys. Rev. B* **2001**, *63* (12), 125120. https://doi.org/10.1103/PhysRevB.63.125120.
(22) Joly, Y.; Bunău, O.; Lorenzo, J. E.; Galéra, R. M.; Grenier, S.; Thompson, B. Self-Consistency, Spin-Orbit and Other Advances in the FDMNES Code to Simulate XANES and RXD Experiments. *J. Phys.: Conf. Ser.* **2009**, *190*, 012007. https://doi.org/10.1088/1742-6596/190/1/012007.
(23) Banerjee, A.; Yue, C.; Choi, J.; Morales-Guio, C. G. Rotating Cylinder Electrode in Reactive CO2 Capture: Identifying Active C Species via Transport, VLE Models and Kinetics. *AIChE Journal 70*, e18560. https://doi.org/10.1002/aic.18560.

Acknowledgements

Work by R.D.R., H.J., A.P. and C.H. was performed under the auspices of the U.S. Department of Energy by Lawrence Livermore National Laboratory (LLNL) under Contract DE-AC52-07NA27344. LLNL release number: LLNL-JRNL-2004639.
